# Molecular pathways in patients with systemic lupus erythematosus revealed by gene-centred DNA sequencing

**DOI:** 10.1136/annrheumdis-2020-218636

**Published:** 2020-10-09

**Authors:** Johanna K Sandling, Pascal Pucholt, Lina Hultin Rosenberg, Fabiana H G Farias, Sergey V Kozyrev, Maija-Leena Eloranta, Andrei Alexsson, Matteo Bianchi, Leonid Padyukov, Christine Bengtsson, Roland Jonsson, Roald Omdal, Benedicte A Lie, Laura Massarenti, Rudi Steffensen, Marianne A Jakobsen, Søren T Lillevang, on behalf of the ImmunoArray Development Consortium and DISSECT consortium, Karoline Lerang, Øyvind Molberg, Anne Voss, Anne Troldborg, Søren Jacobsen, Ann-Christine Syvänen, Andreas Jönsen, Iva Gunnarsson, Elisabet Svenungsson, Solbritt Rantapää-Dahlqvist, Anders A Bengtsson, Christopher Sjöwall, Dag Leonard, Kerstin Lindblad-Toh, Lars Rönnblom

**Affiliations:** 1 Department of Medical Sciences, Rheumatology, Uppsala University, Uppsala, Sweden; 2 Science for Life Laboratory, Department of Medical Biochemistry and Microbiology, Uppsala University, Uppsala, Sweden; 3 Department of Psychiatry, Washington University, St. Louis, Missouri, USA; 4 Division of Rheumatology, Department of Medicine, Karolinska Institutet and Karolinska University Hospital, Stockholm, Sweden; 5 Department of Public Health and Clinical Medicine/Rheumatology, Umeå University, Umeå, Sweden; 6 Broegelmann Research Laboratory, Department of Clinical Science, University of Bergen, Bergen, Norway; 7 Clinical Immunology unit, Department of Internal Medicine, Stavanger University Hospital, Stavanger, Norway; 8 Department of Medical Genetics, University of Oslo, Oslo, Norway; 9 Institute for Inflammation Research, Center for Rheumatology and Spine Diseases, Copenhagen University Hospital Rigshospitalet, Copenhagen, Denmark; 10 Department of Clinical Immunology, Aalborg University, Aalborg, Denmark; 11 Department of Clinical Immunology, Odense University Hospital, Odense, Denmark; 12 Department of Rheumatology, Oslo University Hospital, Oslo, Norway; 13 Institute of Clinical Medicine, University of Oslo, Oslo, Norway; 14 Department of Rheumatology, Odense University Hospital, Odense, Denmark; 15 Department of Rheumatology, Aarhus University Hospital, Aarhus, Denmark; 16 Institute of Clinical Medicine, Aarhus University, Aarhus, Denmark; 17 Center for Rheumatology and Spine Diseases, Copenhagen University Hospital Rigshospitalet, Copenhagen, Denmark; 18 Institute of Clinical Medicine, University of Copenhagen, Copenhagen, Denmark; 19 Department of Medical Sciences, Molecular Medicine and Science for Life Laboratory, Uppsala University, Uppsala, Sweden; 20 Department of Clinical Sciences Lund, Rheumatology, Lund University, Skane University Hospital, Lund, Sweden; 21 Department of Biomedical and Clinical Sciences, Division of Inflammation and Infection, Linköping University, Linköping, Sweden; 22 Broad Institute of MIT and Harvard, Cambridge, Massachusetts, USA

**Keywords:** lupus erythematosus, systemic, polymorphism, genetic, autoimmunity

## Abstract

**Objectives:**

Systemic lupus erythematosus (SLE) is an autoimmune disease with extensive heterogeneity in disease presentation between patients, which is likely due to an underlying molecular diversity. Here, we aimed at elucidating the genetic aetiology of SLE from the immunity pathway level to the single variant level, and stratify patients with SLE into distinguishable molecular subgroups, which could inform treatment choices in SLE.

**Methods:**

We undertook a pathway-centred approach, using sequencing of immunological pathway genes. Altogether 1832 candidate genes were analysed in 958 Swedish patients with SLE and 1026 healthy individuals. Aggregate and single variant association testing was performed, and we generated pathway polygenic risk scores (PRS).

**Results:**

We identified two main independent pathways involved in SLE susceptibility: T lymphocyte differentiation and innate immunity, characterised by HLA and interferon, respectively. Pathway PRS defined pathways in individual patients, who on average were positive for seven pathways. We found that SLE organ damage was more pronounced in patients positive for the T or B cell receptor signalling pathways. Further, pathway PRS-based clustering allowed stratification of patients into four groups with different risk score profiles. Studying sets of genes with priors for involvement in SLE, we observed an aggregate common variant contribution to SLE at genes previously reported for monogenic SLE as well as at interferonopathy genes.

**Conclusions:**

Our results show that pathway risk scores have the potential to stratify patients with SLE beyond clinical manifestations into molecular subsets, which may have implications for clinical follow-up and therapy selection.

**Key messages**

**What is already known about this subject?**

- The clinical heterogeneity in systemic lupus erythematosus (SLE) is likely due to an underlying molecular diversity that could have implications for therapy.
- In recent years, gene expression, autoantibody profiles and cytokine levels have been used to identify groups of patients with SLE with distinct molecular disease mechanisms.

**What does this study add?**

- We have presented a novel strategy to genetically stratify SLE patients according to involved molecular pathways.
- Using genetic information to stratify patients would have the advantages of providing stable molecular markers for early classification.

**How might this impact on clinical practice or future developments?**

- Our results show that pathway risk scores have the potential to stratify SLE patients beyond clinical manifestations into molecular subsets, which may have implications for clinical follow-up and therapy selection.

## Introduction

Systemic lupus erythematosus (SLE) is characterised by the production of autoantibodies targeting nucleic acids and associated proteins, immune complex formation and inflammation in multiple organs. There is a wide spectrum of clinical manifestations in SLE and extensive heterogeneity in disease presentation between patients; in addition, the treatment response is often unpredictable.^1^ The pathogenesis of SLE has partially been clarified during the last years, and important features are increased expression of type I interferon (IFN) regulated genes, defects in the apoptotic process and activated autoreactive B cells.^1 2^ The reasons behind these abnormalities are both environmental and genetic, and today around 100 SLE susceptibility loci have been identified.^3 4^ Monogenic forms of SLE exist, but for a majority of patients the environment and the cumulative number of susceptibility alleles will influence the risk of developing the disease.^4 5^

To date, the contribution of rare genetic variants and the impact of regulatory variants have not been widely explored in SLE. DNA sequencing has the potential to discover novel SLE associated variants not captured by genotyping arrays. Due to the high cost, whole genome sequencing studies (WGS) in SLE have so far mainly focused on families or smaller samples, as have exome sequencing studies (WES).^6–9^ Today it is feasible to perform targeted sequencing in larger cohorts; however, the number of such studies focusing on SLE is still limited.^10^ Additionally, association analysis for rare variants discovered through sequencing is hampered by low statistical power. Aggregating variants on the gene level or by molecular pathway information is one approach to increase power and gain biological insight from rare variants.^11^

The clinical heterogeneity in SLE is likely due to an underlying molecular diversity that could have implications for therapy. In recent years this has started to be addressed, mainly using gene expression, autoantibody profiles and cytokines to identify groups of patients with SLE with distinct molecular disease mechanisms.^12–14^ Using genetic information to stratify patients would have the advantage of providing stable molecular markers for early classification.

Here, we performed targeted sequencing of regulatory and coding regions in a Swedish SLE case–control cohort. We aimed at elucidating the genetic aetiology of SLE from the immunity pathway level to the single variant level, and stratify patients with SLE into molecular subgroups. Altogether around 9% of all genes in the human genome were analysed based on their role in immune-mediated diseases. Gene regions were extended to include promoters and other potentially regulatory elements based on mammalian conservation.^15^

## Methods

For full details on methods see online supplemental methods.

10.1136/annrheumdis-2020-218636.supp1 Supplementary data

### Subjects and DNA samples

The Swedish SLE cohorts included patients recruited at five rheumatology clinics and the controls were healthy blood donors and population controls. The quality-controlled dataset comprised 958 patients with SLE and 1026 control individuals. Patients with SLE fulfilled at least four of the classification criteria for SLE as defined by the American College of Rheumatology (ACR).^16 17^ Clinical characteristics of the patients are available in online supplemental tables S1A and B.

10.1136/annrheumdis-2020-218636.supp2 Supplementary data

### Targeted DNA sequencing analysis

Targeted DNA sequencing was performed in the Swedish SLE case–control cohorts. A SeqCap EZ Choice XL sequence capture panel was designed, libraries were prepared as described elsewhere^18^ and sequenced on an Illumina HiSeq 2500. An overview of the variant discovery and quality control steps can be found in online supplemental figure S1. Study subjects falling outside of the European subpopulation of the Human Genome Diversity Project (HGDP) reference set were excluded (online supplemental figure S7).^19^ The quality-controlled dataset contained 287 354 single-nucleotide variants (SNVs) and covered 1832 of the targeted gene regions.

10.1136/annrheumdis-2020-218636.supp3 Supplementary data

### Genetic association analyses

Several variant sets were generated for aggregate association testing: (1) 1832 individual gene variant sets; (2) 35 pathway variant sets based on the Kyoto Encyclopaedia of Genes and Genomes (KEGG)^20^; (3) five literature review gene sets: the type I interferon pathway,^21^ interferonopathy genes,^22 23^ SLE Genome-Wide Association Study (GWAS) genes,^3 4^ the complement subset of KEGG hsa04610 and genes causing monogenic SLE or lupus-like disease.^24^ Aggregate association testing was performed using Sequence Kernel Association Optimal Test (SKAT-O) or GenePy.^25 26^ Single variant association analyses were performed in PLINK. SLE case-only variants were identified by removing all SNVs present in our Swedish control dataset, the SweGen project or the Genome Aggregation Database European non-Finnish controls.^27 28^

### Risk scores and cluster analysis

Cumulative pathway SLE polygenic risk scores (pathway PRSs) were assigned to each individual based on SNVs associated with SLE at nominal significance. For each independent SNV the natural logarithm of the OR for SLE susceptibility was multiplied by the number of minor alleles in each individual. The sum of all products of all genes in each of the 35 KEGG pathways for each patient was defined as the individual pathway PRS. Hierarchical cluster analysis of pathway PRSs was used to identify groups of patients with SLE.

### Replication study and meta-analysis

Replication genotyping in individuals from Norway and Denmark was performed using the MassARRAY system. The Swedish SLE case–control study was expanded to include an additional 1000 control individuals.^27^ The Scandinavian meta-analysis included 1794 patients with SLE and 3241 control individuals.

## Results

We performed a DNA sequencing study in SLE to study immunity pathways, an overview of analyses can be found in online supplemental figure S2.

### T lymphocyte differentiation and innate immunity pathways are associated with SLE

The sequencing data analysis focused on 1832 genes with relevance for immune-mediated diseases. These genes mainly belong to 35 molecular signalling pathways as defined by the KEGG database (online supplemental table S2).^20^ Using an aggregate test for all variants in the genes belonging to each pathway, we found that 21 of the tested pathways were associated with SLE (false discovery rate (FDR) <0.05, table 1 and online supplemental table S3). The most significantly associated pathways included T helper cell differentiation pathways, with Th1 and Th2 cell differentiation as the top result (FDR_Th1-2_=2.2×10^-9^; FDR_Th17_=1.5×10^-8^), followed by antigen processing and presentation (FDR=3.1×10^-9^).

**Table 1:** SLE case–control pathway based aggregate association analysis

Pathway	Genes in pathway	Genes in test	SNVs in test	P value*	FDR†
Th1 and Th2 cell differentiation (hsa04658)	92	78	14 362	6.3E-11	2.2E-09
Antigen processing and presentation (hsa04612)	77	40	8017	1.8E-10	3.1E-09
Hematopoietic cell lineage (hsa04640)	97	71	13 013	3.8E-10	4.5E-09
Th17 cell differentiation (hsa04659)	107	96	19 347	1.7E-09	1.5E-08
Intestinal immune network for IgA production (hsa04672)	49	39	7909	3.4E-08	2.4E-07
Natural killer cell-mediated cytotoxicity (hsa04650)	131	100	15 821	4.7E-06	2.8E-05
TNF signalling pathway (hsa04668)	112	88	12 639	1.9E-05	9.4E-05
JAK-STAT signalling pathway (hsa04630)	162	133	18 003	7.4E-05	0.00032
RIG-I-like receptor signalling pathway (hsa04622)	70	63	8459	0.00021	0.00080
NOD-like receptor signalling pathway (hsa04621)	178	109	15 729	0.00031	0.0011
Complement and coagulation cascades (hsa04610)	79	50	7112	0.00041	0.0013
Toll-like receptor signalling pathway (hsa04620)	104	96	12 178	0.00080	0.0022
Cytokine-cytokine receptor interaction (hsa04060)	294	221	26 771	0.00083	0.0022
C-type lectin receptor signalling pathway (hsa04625)	104	75	12 986	0.0020	0.0050
IL-17 signalling pathway (hsa04657)	93	68	9358	0.0043	0.0100
Fc epsilon RI signalling pathway (hsa04664)	68	51	8514	0.0052	0.011
Viral protein interaction with cytokine and receptor (hsa04061)	100	75	8435	0.0062	0.013
NF-kappa B signalling pathway (hsa04064)	102	88	14 349	0.0078	0.015
Osteoclast differentiation (hsa04380)	128	101	18 602	0.013	0.023
T cell receptor signalling pathway (hsa04660)	103	85	14 268	0.014	0.025
Cytosolic DNA-sensing pathway (hsa04623)	63	40	4993	0.015	0.025

Pathways with FDR <0.05 in the association analysis including all genes are presented.

*SKAT-O SLE case-control association p value.

†SKAT-O SLE case–control association FDR.

FDR, false discovery rate; IL-17, interleukin 17; NF, nuclear factor; NOD, nucleotide-binding oligomerisation domain; RIG, retinoic acid-inducible gene; SKAT-O, sequence kernel association optimal test; SLE, systemic lupus erythematosus; SNV, single-nucleotide polymorphism; TNF, tumour necrosis factor.

We next explored a sequential elimination strategy to identify independent pathway associations. First, removing all Th1 and Th2 pathway genes in the pathway aggregate association test resulted in the antigen processing and presentation pathway as the top result (FDR=4.8×10^-6^). Second, antigen processing and presentation as well as Th1 and Th2 pathway genes were removed, which resulted in Complement and coagulation cascades as the top result (FDR=0.0091). Third, also genes in this pathway were removed, and the janus kinase-signal transducers and activators of transcription (JAK-STAT) pathway became the top result (FDR=0.014). Lastly, when removing genes in all these four pathways no significant pathways remained. Thus, our data point to two main routes with genetic evidence of association to SLE: T cell differentiation and innate immunity.

**Figure 1:**
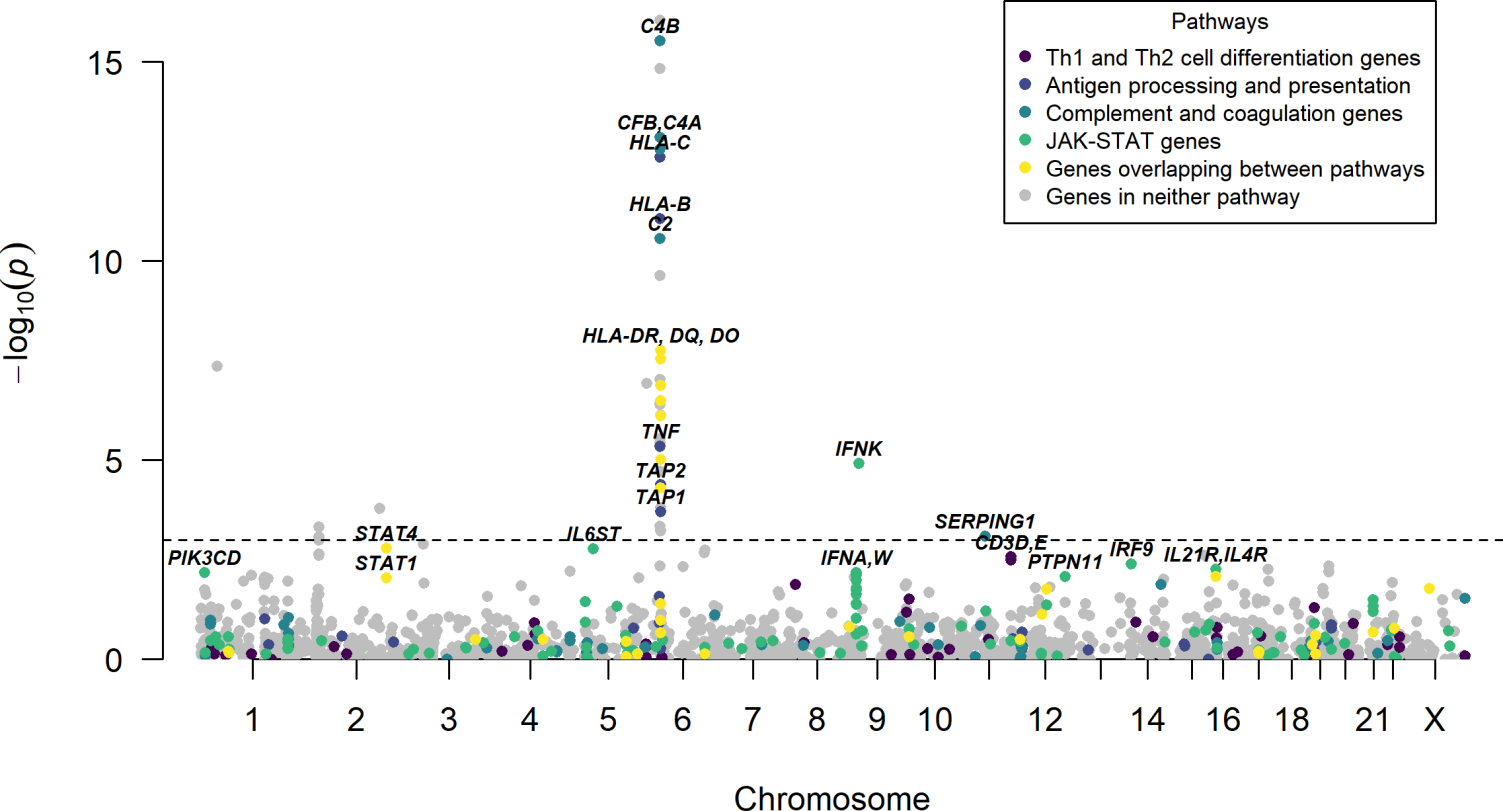
Results of SLE case–control gene-based association analyses. P values for association plotted against chromosomal location, where each point represents a gene region. The line indicates a false discovery rate of 5%. The y-axis has been cut at p=1×10^-15^. Genes belonging to the T-cell differentiation (Th1 and Th2), antigen processing and presentation, complement and coagulation or JAK-STAT signalling pathways are highlighted, and their most significant genes or gene regions are indicated by name. IFNK, interferon kappa; IL21, interleukin 21; SLE, systemic lupus erythematosus.

To identify the genes that underlie the association signals in the T-cell differentiation, antigen processing and presentation, Complement and coagulation and JAK-STAT pathways, gene-based association testing was performed (figure 1). The top association for the JAK-STAT pathway originated from the IFN kappa (IFNK) gene region. SLE-associated genes in the T cell differentiation and antigen processing and presentation pathways were dominated by genes in the HLA region, and for the complement and coagulation cascade pathway, complement genes located in the HLA region were highly significantly associated with SLE.

### Pathway PRS define subsets of patients with SLE

**Figure 2:**
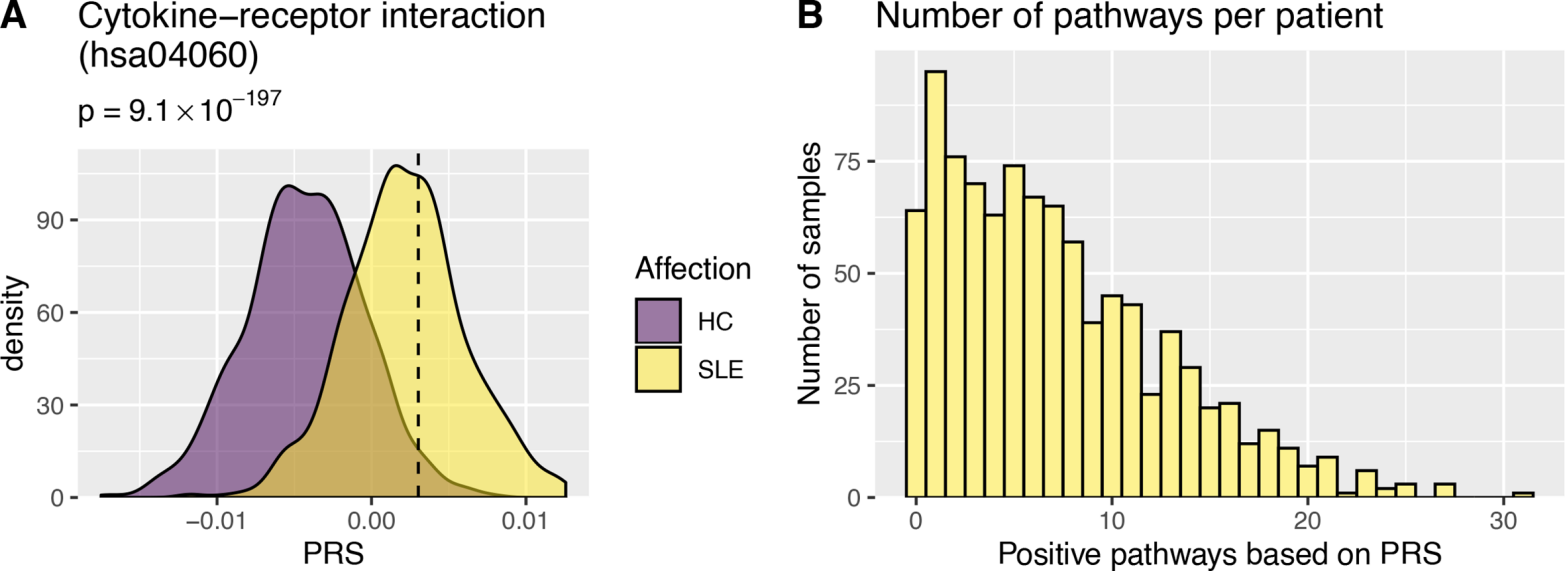
Pathway SLE polygenic risk scores. (A) Illustrates pathway Polygenic Risk Scores (PRS) for the Cytokine–cytokine receptor interaction pathway. P values represent differences in PRS between patients with SLE (SLE) and healthy control individuals (HC). The dashed line indicates the PRS 97.5 percentile in control individuals. (B) The number of pathways each individual patient with SLE tested positive for using the pathway PRS. On average patients were positive for 7.2 pathways. SLE, systemic lupus erythematosus.

**Figure 3:**
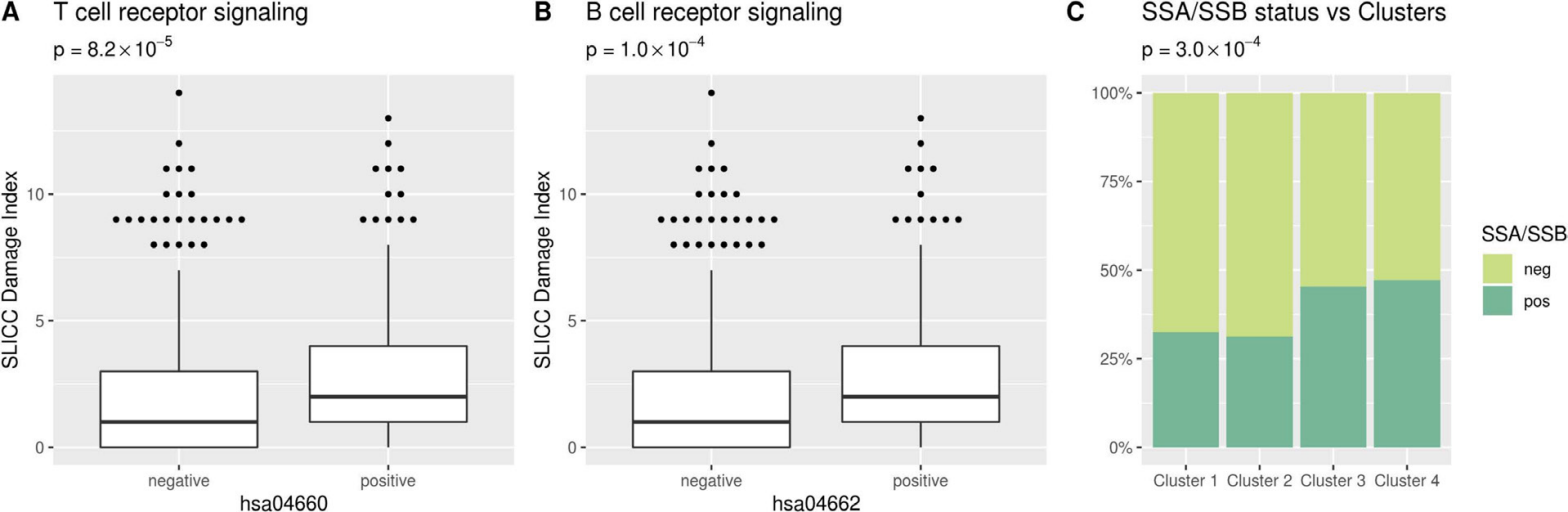
Pathway SLE polygenic risk scores grouping and clustering. (A, B) The Systemic Lupus Erythematosus International Collaborating Clinics (SLICC) damage index for patients with SLE positive and negative for the T cell receptor and B cell receptor signalling pathways. P values represent differences in Damage Index between pathway positive and negative patients, uncorrected p values are presented (Bonferroni corrected threshold p=0.00143). (C) Prevalence of Sjögren’s syndrome (SSA and/or SSB) autoantibodies in SLE patients in the four clusters. P value represent difference in SSA/SSB autoantibody status between clusters of SLE patients, uncorrected p value is presented (Bonferroni corrected threshold p=0.002).

Having identified pathways with genetic association with SLE, we hypothesised that different patients with SLE could have distinct pathways affected. We constructed pathway PRS for each individual and each of the pathways, by combining the burden of common SLE associated alleles from our sequencing data. Individuals with a pathway PRS higher than that observed for the 97.5th percentile of control individuals were classified as positive for that pathway (online supplemental figure S3). The largest proportion of positive SLE patients was observed for the Cytokine-cytokine receptor interaction pathway (41%, figure 2A, and online supplemental table S4). For the Th1 and Th2 cell differentiation, antigen processing and presentation, Complement and coagulation cascades and JAK-STAT signalling pathways 18%, 16%, 21% and 29% of patients with SLE were positive, respectively. On average each SLE patient tested positive for the pathway PRS for seven pathways (figure 2B). As we had previously observed that a high SLE genetic risk score was associated with organ damage in SLE, we investigated whether this could be observed for specific pathways.^5^ We found that the SLE International Collaborating Clinics Damage Index was significantly higher in the SLE patients positive for the T cell or B cell receptor signalling pathways (figure 3A, B). No other pathways were associated with clinical manifestations of SLE or survival.

**Figure 4:**
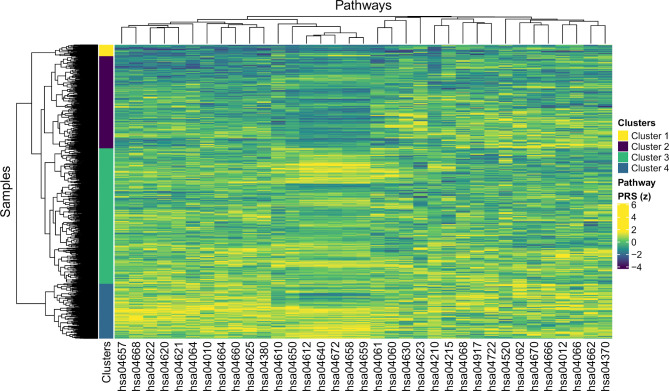
Clustering of patients with SLE based on pathway Polygenic Risk Scores (PRS). Heat map with pathways on the x-axis (KEGG IDs) and individuals on the y-axis based on normalised PRS. Hierarchical cluster analysis was performed based on the PRS per pathway for each individual. The colour bar on the left indicates the four main clusters of individuals identified. KEGG, Kyoto Encyclopaedia of Genes and Genomes; SLE, systemic lupus erythematosus.

We then performed a hierarchical cluster analysis on the pathway PRSs in SLE, to identify groups of patients with similar molecular aetiology. Four clusters of patients were identified (figure 4). The pathway with the most significant difference in PRS between clusters was the antigen processing and presentation pathway, followed by Th17 cell differentiation (online supplemental figure S4). Next, we investigated whether the molecular stratification of patients with SLE also mirrored differences in clinical presentation between groups. We found that the presence of autoantibodies against Sjögren’s syndrome-related antigens SSA and/or SSB was more common among patients in clusters 3 and 4 (figure 3C). We did not observe any significant difference in other clinical features, including survival, between the four patient clusters.

### Common variants contribute risk at monogenic risk loci in SLE

We then focused our analysis on gene-sets with prior evidence for involvement in SLE, but which were not defined in KEGG, to investigate the impact of both rare and common variants for these groups of genes. We found that interferon system, interferonopathy, SLE GWAS, complement system and monogenic SLE and lupus-like disease genes in aggregate were associated with SLE when analysing variants of all minor allele frequencies (MAF) (table 2 and online supplemental table S5). Only the monogenic SLE and lupus-like disease gene-set was significantly associated with SLE when separately analysing the rarer variant (MAF <0.01) contribution (table 2). There was a clear common variant (MAF >0.05) contribution to associations for the interferonopathy, SLE GWAS, complement system and monogenic SLE and lupus-like disease gene-sets (table 2).

**Table 2:** Gene-set analyses of SLE-associated genes and involved pathways

Set name	Genes tested	No of SNVs all/common/rare	FDR_ALL_	FDR_COMMON_	FDR_RARE_
Interferon (ref 21)	33	4204/849/2866	0.0018	0.66	0.65
Interferonopathy (ref 22,23)	11	2034/463/1271	0.0028	4.1E-07	0.24
SLE GWAS (ref 3,4)	88	18790/5326/11465	1.5E-12	2.0E-15	0.18
Complement*	32	4712/1094/3086	0.00071	2.8E-07	0.20
Monogenic SLE (ref 24)	24	3745/930/2371	2.9E-07	2.9E-11	0.020

All: including all MAFs; Common: MAF >0.05; Rare: MAF <0.01.

*The complement part of KEGG pathway hsa04610.

FDR, false discovery rate; GWAS, genome-wide association study; KEGG, kyoto encyclopedia of genes and genomes; MAF, minor allele frequency; SLE, systemic lupus erythematosus; SNV, single-nucleotide variant.

### Potentially novel SLE risk loci

Next, we asked whether we could detect novel SLE risk loci, regardless of pathway or gene-set membership. Two potentially novel gene regions passed a Bonferroni corrected threshold in the gene-based SLE case–control association analyses: *PABPC4* (p=4.3×10^-8^) and *IFNK* (p=1.2×10^-5^, online supplemental figure 5A, tables S6 and S7). In single variant association analyses, we observed SNV associations at three potentially novel SLE risk loci, *CAPN13*, *MOB3B/IFNK* and *HAL*, at a suggestive significance threshold (p<1×10^-4^, online supplemental figure 5B–E, table S8). As the association signals at *CAPN13*, *MOB3B/IFNK* and *HAL* had not been reported in SLE GWAS in other ancestries, we attempted to replicate these findings in additional Scandinavian SLE cases and controls (online supplemental table S1A). However, we did not find additional support for a role of SNVs at these novel loci in SLE (online supplemental table S9).

### Patients with SLE carry unique coding variants

We next investigated whether there was an increased rare coding mutational burden for patients with SLE at the 1832 genes. We observed that all individuals carried rare non-synonymous variants, with an average number of around 32 variants per individual for both patients with SLE and control individuals (online supplemental figure S6). None of the patients with SLE were homozygous carriers of rare non-synonymous alleles in genes for monogenic SLE and lupus-like diseases (online supplemental table S10). Next, we hypothesised that protein coding variants observed exclusively in patients with SLE could be causal candidates. A total of 1475 case-only nonsynonymous variants were identified in the 958 patients with SLE (online supplemental table S11). These were variants that were observed in at least one patient with SLE, but not in control individuals of similar ancestry.^27 28^ The most frequent of these SNVs was found in the *MUC5B* gene which encodes mucin 5B, the major gel-forming mucin in mucus (table 3). Five patients with SLE carried the same deleterious *MUC5B* missense mutation (rs773068050, p.Thr2724Pro). *MUC5B* gene variants have previously been associated with interstitial lung disease (ILD), a condition affecting around 3% of Swedish patients with SLE.^29–31^ However, there was no evidence of ILD in these five patients, but two of them had suffered from pleuritis (online supplemental table S12). In conclusion, we did not find evidence for SLE patients carrying a generally increased burden of rare coding variants at these genes. However, our analysis identified a number of coding variants observed exclusively in patients with SLE. This catalogue of variants could serve as a resource for future studies investigating the role of case-only SNVs in SLE.

**Table 3:** SLE case-only recurrent non-synonymous SNVs

CHR	BP	SNV	Ref allele	Alt allele	Count SLE	Gene	Consequence	Amino acid change	SIFT
11	1 266 280	rs773068050	A	C	5	*MUC5B*	Missense variant	p.Thr2727Pro	Deleterious(0.05)
1	186 363 103	rs1292231132	C	A	4	*C1orf27*	Missense variant	p.Gln246Lys	Tolerated(0.21)
1	151 342 270	rs772030489	G	T	2	*SELENBP1*	Missense variant	p.Pro36Thr	Deleterious low confidence(0.01)
2	27 455 971	rs776014297	T	A	2	*CAD*	Missense variant	p.Met922Lys	Deleterious(0.04)
2	179 698 928	rs892049188	G	A	2	*CCDC141*	Missense variant	p.Ser1522Phe	Tolerated(0.08)
9	16 431 447	chr9:16 431 447	G	A	2	*BNC2*	Missense variant	p.His307Tyr	–
9	21 166 175	rs779242420	T	C	2	*IFNA21*	Missense variant	p.Tyr146Cys	Deleterious(0.01)
10	75 583 821	chr10:75 583 821	G	T	2	*CAMK2G*	Missense variant	p.His370Asn	Deleterious low confidence(0.03)
12	6 458 353	rs775543049	G	A	2	*SCNN1A*	Stop gained	p.Arg551*	–
12	48 482 728	rs750735162	T	C	2	*SENP1*	Missense variant	p.Thr155Ala	Deleterious low confidence(0)
12	56 350 882	rs1425141530	G	T	2	*PMEL*	Missense variant	p.Pro402His	Deleterious(0.02)
12	129 190 793	rs1386045604	C	G	2	*TMEM132C*	Missense variant	p.Pro1094Ala	Tolerated(0.21)
14	23 057 866	chr14:23 057 866	A	T	2	*DAD1*	Missense variant	p.Ser66Arg	Deleterious(0.04)
15	91 030 272	rs181919733	G	A	2	*IQGAP1*	Missense variant	p.Val1371Met	Tolerated(0.07)
17	41 143 320	rs1456586259	G	A	2	*RUNDC1*	Missense variant	p.Val477Ile	Tolerated(0.12)
19	4 891 395	rs139019426	T	C	2	*ARRDC5*	Missense variant	p.Gln231Arg	Tolerated(0.86)
19	18 273 781	rs777121279	G	A	2	*PIK3R2*	Missense variant	p.Gly372Ser	Deleterious(0)
19	55 240 959	rs764066889	G	A	2	*KIR3DL3*	Missense variant, splice region variant	p.Gly219Asp	Deleterious(0.02)

SIFT (Sorting Intolerant From Tolerant) prediction whether the amino acid substitution affects protein function.

BP, base pair; CHR, chromosome; SLE, systemic lupus erythematosus; SNV, single-nucleotide variant.

## Discussion

We here suggest a novel pathway-based approach to stratify patients with SLE beyond clinical manifestations. Further, we characterise genetic pathway associations and investigate rare variant contributions to the pathogenesis of SLE, all using targeted sequencing of immunity genes.

Using case–control association testing for immunological pathways, we identified two main axes of SLE association: T cell differentiation and innate immunity pathways. T cells have a fundamental role in loss of tolerance, autoimmunity and inflammatory reactions. In SLE, a number of different T cell disturbances have been described, which can contribute to the generation of autoreactive T cells, aberrant cytokine production and impaired T regulatory cell function.^32^ Besides the direct involvement of pathways connected to Th1 and Th2 cells, we noticed association signals from two pathways related to interleukin 17 (IL-17). A proportion of patients with SLE display raised serum levels of IL-17, elevated numbers of circulating IL-17-producing T cells and increased IL-17 production by lymphocytes, suggesting dysregulation of T regulatory cells.^33^ Our findings strengthen the recent suggestions that IL-17 inhibition could be a therapeutic option in a subset of patients with SLE.^34^ Conversely, low-dose IL-2 treatment in SLE to stimulate T regulatory cells has recently shown promising results.^35^

We observed that the T cell differentiation pathway associations were influenced by genetic associations to HLA, which is not surprising given the essential role of HLA in the immune response. This was further demonstrated by the antigen processing and presentation pathway association dominated by HLA genes. Complement pathway associations are also possibly confounded by the HLA SLE association, since early complement component genes are located in the HLA class III locus on chromosome 6.^36^ The JAK-STAT pathway was associated with SLE, it is the main route to initiate gene expression and protein synthesis for over 50 cytokines, many of which are involved in the SLE disease process.^37 38^ Variants of a number of genes in the JAK-STAT pathway have been associated with an increased risk for SLE, for instance *STAT4-STAT1* and *TYK2*.^3 4^

Our study highlights the importance of the interferon system in SLE. Previous studies have shown genetic associations at a number of genes in the IFN signalling pathway in SLE.^2 3^ Here, we show that, in aggregate, genetic variation at interferonopathy genes also contribute to risk for SLE. In addition to interferonopathy genes, we also observed an aggregate genetic association for monogenic SLE and lupus-like disease genes with both a rare and a common variant contribution. This supports the hypothesis of a shared genetic basis and consequently disease mechanisms between monogenic and complex forms of disease, where also common non-coding variants can affect the regulation of Mendelian disease genes resulting in clinically similar traits.^39^

We have previously demonstrated that an SLE genetic risk score was associated with disease severity in SLE.^5^ We here generated a pathway-centred SLE PRS and found that there was a large variation in the number of affected pathways among the patients, which underscores the heterogeneity of SLE. We observed higher SLE damage indexes in patients with SLE positive for the B or T cell receptor signalling pathways, thus, pathways in the adaptive immune system seem important for the long-term severity of the disease. This is in accordance with previous findings that SLE disease activity correlates with abnormal B lymphocyte activity and T cell abnormalities, as well as the connection between disease activity and accumulation of organ damage.^40 41^

We attempted to cluster patients into subsets with shared genetic pathway profiles, which suggested four subgroups of patients with SLE. Beside the SSA/SSB antibody profile, these clusters were not connected to clinical disease manifestations such as nephritis or survival. This observation may indicate that the PRS reflects part of the central autoimmune process, which is not translated into specific organ manifestations. Whether the PRS in individual patients with SLE, or the different clusters, contribute to treatment response is an interesting possibility, but could not be assessed in this study. This is one limitation of our study, together with the fact that our conclusions apply specifically to this set of candidate genes.

WGS or WES studies will be required to fully elucidate the role of rare variants and pathways in SLE. As previously shown by us and others, WGS and WES in selected patients can provide information on ultrarare and de novo SNVs in SLE.^6 7 42^ However, larger sample sizes than those reported to date will be required to paint a complete picture of the genetic aetiology of SLE. We did not find support in additional Scandinavian cohorts for a role in SLE for the novel loci identified in the Swedish cohorts. Possible explanations include overestimated effect sizes in the discovery cohort, differences in genetic background within Scandinavia, or differences in clinical manifestations or characterisation of patients. Lastly, our study identified a large number of case-only coding variants. Variants uniquely identified in patients could be causal candidates in SLE, but their statistical significance is difficult to evaluate.

In summary, we have suggested a novel strategy to genetically stratify patients with SLE according to involved molecular pathways. T cell pathways displayed the strongest association, which highlights the importance of the adaptive immune system in the disease. The strong connection to the JAK-STAT pathway, including the IFN system, is perhaps not surprising given the promising clinical trials of JAK and type I interferon receptor inhibition as treatments for SLE.^38 43 44^ However, not all patients in these studies respond to treatment, and dissecting affected molecular pathways in responders and non-responders could increase the understanding of treatment outcome. This approach has not been tested clinically, but the future of precision medicine for SLE lies in identifying robust methods to perform molecular stratification of patients.
