# Supplementary material for: Molecular pathways in patients with systemic lupus erythematosus revealed by gene-centred DNA sequencing
Source: Ann Rheum Dis. 2020 Oct 9;80(1):109–17. doi: 10.1136/annrheumdis-2020-218636 (PMC7788061; doi:10.1136/annrheumdis-2020-218636)
Supplement: Supplementary data [file annrheumdis-2020-218636supp001.pdf]

Molecular Pathways in Patients with Systemic Lupus Erythematosus Revealed by Gene Centred DNA Sequencing

Sandling et al.

Contents:

Supplementary Materials and Methods

Supplementary Materials and Methods

Subjects and DNA samples

The Swedish SLE cohorts included 1,167 SLE patients recruited at the Rheumatology clinics at the Uppsala, Karolinska (Solna), Umeå, Lund and Linköping University Hospitals. Blood samples and clinical information originated from time of diagnosis, study inclusion or follow-up visits, and clinical information was compiled at the end of follow-up. An extended follow-up was performed specifically for death as outcome. The controls were healthy blood donors or population controls from Uppsala Bioresource and Västerbotten biobank in Sweden (n=1,101). Genomic DNA extracted from blood samples was available for genetic analysis. DNA samples for sequencing were selected based on DNA amount and quality if multiple DNA samples were available for the same individual. The quality-controlled dataset used in subsequent analyses contained 958 SLE patients and 1,026 control individuals. All 958 SLE patients fulfilled at least four of the classification criteria for SLE as defined by the American College of Rheumatology (ACR).(1, 2) Renal biopsies were classified according to the WHO or the ISN/RPS 2003 classification systems.(3) Clinical characteristics of the patients are available in online supplementary Tables S1a and S1b. All subjects provided informed consent to participate in the study, and the study was approved by the regional ethics board in Uppsala (Dnr 2015/450 and 2016/155).

Targeted DNA sequencing

Targeted DNA sequencing was performed in the Swedish SLE case-control cohorts. The design of the sequence capture panel and the library preparation has been described elsewhere.(4) In brief, a custom SeqCap EZ Choice XL library (Roche NimbleGen, Basel, Switzerland) was designed to target 1,853 genes, selected based on their known or suspected roles in immunological or autoimmune diseases in humans or model organisms.(4) The genomic intervals for all alternative transcripts were retrieved from NCBI36/hg18. Besides the coding exons, 5’ and 3’ UTRs, potential promoter regions (±2 kb from transcription start sites) and splice sites (±20 bp of intronic sequences adjacent to exons)

were also included, as well as regions of mammalian conservation within 100kb up- and downstream of the genes.(5) In total, the designed probes covered 32.3 Mbp. Sequencing libraries were prepared by ultrasonication of up to 1 µg of high molecular weight DNA into around 400 bp fragments (Covaris E220, Woburn, MA, USA), that were then barcoded (NEXTflex-96 DNA barcode adapters, Bioo Scientific, Austin, TX, USA). Samples were pooled in batches of eight, hybridized (Roche NimbleGen) and sequenced with 100-bp paired-end reads using Illumina HiSeq 2500 version 3 or 4 chemistry (Illumina Inc, San Diego, CA, USA). An average sequencing depth of 35× per sample was achieved.

Alignment and variant calling

A pipeline based on GATK "best practices" was used for variant discovery.(6) Raw reads were mapped to the hg19 human reference genome using the Burrows-Wheeler aligner 0.7.12 (7) and duplicate reads marked by Picard 1.92. GATK 3.3.0 was applied for realignment around indels, base quality score recalibration, SNP and indel discovery and joint genotyping. Prior to genotyping, alignment quality was evaluated by Samtools flagstat (7) and Picard tools CalculateHSMetrics and samples with mean target coverage less than 10x were excluded. From this point on, only bi-allelic single nucleotide variants (SNVs) were considered. SNV quality scores were recalibrated using GATK 3.3.0 VariantRecalibrator and filtered at tranche level 99.0. Using VCFtools,(8) genotype calls with depth less than 8 reads and genotype Phred quality score less than 20 were excluded.(9)

Sample and variant level quality control

Study population genetic structure was analysed by the LASER software using default parameters and the Human Genome Diversity Project (HGDP) as reference population (online supplementary Figure S7a).(10, 11) Population outliers were defined using the following criteria: 1) study subjects falling more than five standard deviations outside of the mean of the European sub-population of the HGDP reference set were excluded, 2) mean and standard deviation were calculated for the remaining study subjects and any additional subjects falling more than five standard deviations outside of the

study mean were excluded, 3) step 2 was repeated until no additional subjects were excluded. Relatedness among study subjects was determined using the KING software, applying default thresholds for duplicate and first degree relationships.(12) Extreme sample outliers were identified based on several quality control (QC) measures, as suggested by Do et al.(13) These QC parameters included rate of missing data, heterozygosity ratio, transition-transversion ratio and singleton counts. Further, samples were excluded if they exhibited discordance between reported sex and that inferred from sequence data or if they exhibited discordance between genotypes inferred from sequence data and a genotype dataset from a previous study.(14) Lastly, it was required that samples had a minimum call rate of 80%.

A number of filters were applied to exclude low quality variants. Heterozygous calls were included only if their allelic balance across all samples was between 0.2 and 0.8. Positions deviating from Hardy-Weinberg equilibrium ($P < 1x10^{-6}$, calculated on controls) were excluded as well as monomorphic sites. Finally, a minimum of 90% variant call rate was required. The remaining variant positions were investigated for differential missingness between cases and controls using PLINK (15), and significantly different variants were excluded ($P < 0.05$ Bonferroni corrected). An overview of the QC steps can be found in online supplementary Figure S1. The quality-controlled dataset used in subsequent analyses contained 958 Swedish SLE patients, 1,026 control individuals, 287,354 SNVs and covered 1,832 of the targeted gene regions. The average individual call rate was 98.2% and the average variant call rate 98.2%. Genotypes from targeted sequencing were validated using an independent genotype array dataset (Illumina ImmunoChip) on an overlapping set of 1,693 Swedish individuals and 8,483 SNVs after QC.(14) SNV genotype concordance was on average 99.8% (online supplementary Figure S8).

Variant level annotation

Variant annotation was performed using SnpEff v4.2.(16) Non-synonymous variants were defined as SNVs annotated as missense or nonsense variants. Non-coding SNVs were defined as SNVs annotated

to upstream, downstream, intergenic regions or regions overlapping transcription factor binding sites, but not as missense or nonsense SNVs. The extended HLA region spanning a region of 7.9 Mbp was defined as from the gene *SCGN* to *SYNGAP1* on hg19 chr6:25,652,429-33,560,852.(17) Evolutionarily constrained positions were defined as having a Genomic Evolutionary Rate Profiling (GERP) rejected substitutions (RS) score >2.(18) In analyses of rare SNVs, variants with MAFs <0.01 were included, and for common SNVs variants with MAFs ≥0.05 were included.

Single variant analyses

Principal components for population stratification were generated in EIGENSOFT (19) after excluding long-range linkage disequilibrium (LD) regions (20), SNVs with MAF<0.05 and SNVs in LD r^2>0.2 (online supplementary Figure S7b-d). Single variant association analyses for variants with MAF≥0.01 were performed in PLINK using a logistic regression model, in which the three first principal components were added as regression covariates. Two levels of significance were applied, an experiment-wide P-value threshold of 1.8 $x10^{-6}$ (P < 0.05 Bonferroni corrected, limiting LD to r^2<0.2 which resulted in 27,195 variants used for multiple testing correction) and a suggestive threshold of P<$1x10^{-4}$. LD was measured by r^2 calculated in PLINK. Manhattan and QQ plots were generated in R using the package qqman.(21, 22) Regional plots of associations were generated using R. Conditional analysis, using the top SNP from the previous model as covariate, was performed until there were no residual association signals below the suggestive threshold (P <$1x10^{-4}$). Differences in variant load between SLE patients and controls were assessed by the Mann-Whitney U test. SLE case-only variants were identified by removing all SNVs present in our control dataset of 1,026 individuals, in the SweGen project 1,000 individuals version September 4th, 2017 generated by Science for Life Laboratory(23) or in the Genome Aggregation Database (gnomAD) European non-Finnish controls v2.1.1.(24)

Aggregate association testing

Variant-sets were generated for aggregate association testing using three different strategies. 1) Gene variant-sets for gene-based association testing: The RefSeq annotation of the hg19 human genome assembly was used to assign genomic positions to each target gene.(25) Aggregate spaces were generated such that the minimal transcript start site and the maximal transcript end site of any transcript for each gene was recorded. The spaces were then extended by 100kb on each end to include regulatory regions, except in analyses focusing on rare coding variants only. Variants falling within the same aggregate gene space were assembled into a gene variant set. 2) Pathway variant-sets for pathway-based association testing: Pathway-wide aggregate spaces were generated by utilising information from the Kyoto Encyclopedia of Genes and Genomes (KEGG) on membership of genes in pathways.(26) Pathway spaces were defined as the union of gene spaces of genes annotated to be part of each pathway. Association testing was performed only for pathways that were represented by at least five genes in the sequencing data, and where at least 50% of the genes in the pathway were targeted. Additionally, the Human Diseases class of pathways were excluded. This resulted in 35 KEGG pathway variant-sets for association testing. 3) Literature review gene sets for gene set-based association testing: the type I interferon pathway (27), interferonopathy genes (28, 29), gene variant sets for SLE GWAS genes (14, 30), the complement subset of the Complement and coagulation cascades pathway (KEGG hsa04610) and genes causing monogenic SLE or lupus-like disease (31) were grouped into separate gene sets.

Aggregate association testing was performed separately for each variant-set using SKAT-O with the inclusion of the first three principal components generated in EIGENSOFT as covariates.(32) We employed a weighted linear kernel using the default weights as calculated internally by the beta distribution with parameters a=1 and b=25, giving higher weight to rare variants. To ensure reproducible outcomes we set the random number seed value in R to 1,337 before running SKAT-O. For P-value calculation we used the “hybrid” approach that selects the optimal method based on the

total minor allele count (MAC), the number of individuals with minor alleles (m), and the degree of case-control imbalance. This corrects for conservative type I errors when using a small sample size. FDR were controlled separately for the pathway, gene-set and gene-based SKAT-O aggregate association analyses.

Gene-based aggregate association testing including variant deleteriousness metrics was performed with GenePy.(33) Region annotation and the gene space was dictated by Annovar.(34) GenePy was run with default parameters, using as reference allele frequencies those in the non-Finnish European gnomAD v2.1.1 125,748 exomes dataset. The gene annotation was based on RefSeq (RefSeq gene body + 1Kb upstream and 1Kb downstream). The gene score P-value was obtained by comparing the distribution of gene scores in cases vs controls using a Mann-Whitney U test. Genes were considered statistically significant if their P-value was below the permutation P-value. The permutation threshold was the P-value corresponding to the 5% right tail of the distribution of the lowest P-values obtained by shuffling the phenotypes (disease status) 1,000 times and running a Mann-Whitney U test. Results using the REVEL (prediction of the pathogenicity of rare missense variants) and CADD v1.3 (63 annotations, including conservation metrics, functional genomic data, transcript information and protein level scores) annotations were presented.(35, 36)

Risk scores and cluster analysis

Cumulative pathway SLE polygenic risk scores (pathway PRSs) were assigned to each individual based on SNVs associated with SLE at nominal significance (P <0.05) in the SLE case-control single variant association study. The Plink function “clump” was used to remove SNV in high LD (r^2 > 0.2) within 250 kbp and to only retain those variants with the highest phenotype association. 1,296 SLE associated SNVs were retained. Then, for each SNV, the natural logarithm of the OR for SLE susceptibility was multiplied by the number of minor alleles in each individual. The sum of all products of all genes in each of the 35 KEGG pathways for each patient was defined as the individual pathway PRS.

Hierarchical cluster analysis with complete linkage on the Euclidean distance between scaled individual level pathway PRS was used to identify clusters of SLE patients. The NBClust R package was used to determine the optimal number of clusters by majority voting and four clusters were determined to be optimal.(37) A heatmap of scaled values of pathway specific PRS was plotted using the R package ComplexHeatmap.(38) A Chi² test was used to determine if the clusters differed in composition for case/control status or dichotomous sub phenotypes in SLE patients, while a Mann-Whitney U test was used to determine if quantitative traits differ between the SLE patients in both clusters, or if the pathway PRS values differed between SLE cases and healthy controls. Kruskal–Wallis one-way analysis of variance was used to determine if pathway PRS values differ between clusters.

Replication study and meta-analysis

The replication study included Norwegian and Danish SLE cohorts recruited at the Oslo University Hospital, Rigshospitalet in Copenhagen, Odense University Hospital and Aarhus University Hospital. Only SLE patients fulfilling the ACR SLE classification criteria and of self-reported European ancestry were included in association analyses. Norwegian and Danish control individuals from the University Hospitals in Stavanger, Bergen, Odense, Aalborg and Rigshospitalet in Copenhagen were also included. All subjects provided informed consent to participate in the study, and the study was approved by the regional ethics boards. 20 SNVs representing association signals at three loci (*CAPN13*, *IFNK/MOB3B*, *HAL*) or their proxies (LD $r^2 \geq 0.99$) were either genotyped or extracted/imputed from existing sequencing or GWAS array data.

Genotyping was performed using the iPLEX chemistry on a MassARRAY system (Agena Bioscience, San Diego, CA, USA). QC included a minimum per sample call rate of 90% and a per variant call rate of 90%. Variants with differential missingness between cases and controls (P <0.01) or Hardy-Weinberg equilibrium (P <0.01, in controls) were excluded. 836 Norwegian and Danish SLE patients and 782 Danish healthy control individuals passed QC. Quality-controlled genotype data for 143 Norwegian

healthy control individuals was extracted from targeted sequencing data.(39) Replication variants not called in the sequencing data were imputed with the Sanger imputation service using the Haplotype reference consortium r1.1 reference dataset and the "pre-phase with EAGLE2 and impute" pipeline.(40) Imputed genotype calls with genotype probabilities below 0.9 were set to missing and SNVs with a MAF below 0.01, a Hardy-Weinberg equilibrium P<0.0001, a call rate below 95% or an imputation probability score below 0.8 were removed, as were individuals with a call rate below 95%.

124 Norwegian control individuals had been genotyped on the Illumina 550 K BeadChip and 298 individuals on the Affymetrix Genome-Wide Human SNP Array 6.0. Hg19 genome assembly genomic position of variants were assigned based on the rs IDs using the dbSNP version 152 for Illumina or using the annotation file for Affymetrix. Prior to imputation the datasets were filtered for 95% call rate both on the variant and individual level, a minimum MAF of 0.05 and a HWE $P>1\times10^{-4}$. Variants were strand flipped to match the reference allele and variants that could not be resolved were removed. The resulting datasets were imputed and filtered in the same way as the sequencing-based dataset described above. After QC the replication dataset included 15 SNVs, 836 SLE patients and 1,211 control individuals.

The Swedish SLE case-control study was expanded to include genotypes from an additional 1,000 control individuals from the SweGen project version September 4th, 2017 generated by Science for Life Laboratory.(23) Genotypes for proxy variants that were part of the replication study genotyping, but which were not directly called in the targeted sequencing data, were imputed and quality-controlled as above. Single variant association analyses were performed separately for the expanded Swedish, the Norwegian and the Danish case-control studies in PLINK using a logistic regression model. Meta-analysis of the three association studies results was performed in PLINK assuming a random effects model. The meta-analysis included a total of 1,794 Scandinavian SLE patients and 3,241 control individuals.

References

1. Tan EM, Cohen AS, Fries JF, et al. The 1982 revised criteria for the classification of systemic lupus erythematosus. Arthritis and rheumatism. 1982;25(11):1271-7.
2. Hochberg MC. Updating the American College of Rheumatology revised criteria for the classification of systemic lupus erythematosus. Arthritis and rheumatism. 1997;40(9):1725.
3. Weening JJ, D'Agati VD, Schwartz MM, et al. The classification of glomerulonephritis in systemic lupus erythematosus revisited. J Am Soc Nephrol. 2004;15(2):241-50.
4. Eriksson D, Bianchi M, Landegren N, et al. Extended exome sequencing identifies BACH2 as a novel major risk locus for Addison's disease. J Intern Med. 2016;280(6):595-608.
5. Lindblad-Toh K, Garber M, Zuk O, et al. A high-resolution map of human evolutionary constraint using 29 mammals. Nature. 2011;478(7370):476-82.
6. DePristo MA, Banks E, Poplin R, et al. A framework for variation discovery and genotyping using next-generation DNA sequencing data. Nature genetics. 2011;43(5):491-8.
7. Li H, Durbin R. Fast and accurate short read alignment with Burrows-Wheeler transform. Bioinformatics. 2009;25(14):1754-60.
8. Danecek P, Auton A, Abecasis G, et al. The variant call format and VCFtools. Bioinformatics. 2011;27(15):2156-8.
9. Carson AR, Smith EN, Matsui H, et al. Effective filtering strategies to improve data quality from population-based whole exome sequencing studies. BMC Bioinformatics. 2014;15:125.
10. Wang C, Zhan X, Bragg-Gresham J, et al. Ancestry estimation and control of population stratification for sequence-based association studies. Nature genetics. 2014;46(4):409-15.
11. Wang C, Zhan X, Liang L, et al. Improved ancestry estimation for both genotyping and sequencing data using projection procrustes analysis and genotype imputation. Am J Hum Genet. 2015;96(6):926-37.
12. Manichaikul A, Mychaleckyj JC, Rich SS, et al. Robust relationship inference in genome-wide association studies. Bioinformatics. 2010;26(22):2867-73.
13. Do R, Stitziel NO, Won HH, et al. Exome sequencing identifies rare LDLR and APOA5 alleles conferring risk for myocardial infarction. Nature. 2015;518(7537):102-6.
14. Langefeld CD, Ainsworth HC, Cunninghame Graham DS, et al. Transancestral mapping and genetic load in systemic lupus erythematosus. Nature communications. 2017;8:16021.
15. Purcell S, Neale B, Todd-Brown K, et al. PLINK: a tool set for whole-genome association and population-based linkage analyses. Am J Hum Genet. 2007;81(3):559-75.
16. Cingolani P, Platts A, Wang le L, et al. A program for annotating and predicting the effects of single nucleotide polymorphisms, SnpEff: SNPs in the genome of Drosophila melanogaster strain w1118; iso-2; iso-3. Fly. 2012;6(2):80-92.
17. Horton R, Wilming L, Rand V, et al. Gene map of the extended human MHC. Nature reviews Genetics. 2004;5(12):889-99.
18. Cooper GM, Stone EA, Asimenos G, et al. Distribution and intensity of constraint in mammalian genomic sequence. Genome Res. 2005;15(7):901-13.
19. Price AL, Patterson NJ, Plenge RM, et al. Principal components analysis corrects for stratification in genome-wide association studies. Nature genetics. 2006;38(8):904-9.
20. Price AL, Weale ME, Patterson N, et al. Long-range LD can confound genome scans in admixed populations. Am J Hum Genet. 2008;83(1):132-5; author reply 5-9.
21. RCoreTeam. R: A language and environment for statistical computing. R Foundation for Statistical Computing, Vienna, Austria. 2013.
22. Turner SD. qqman: an R package for visualizing GWAS results using Q-Q and manhattan plots. J Open Source Software. 2018;3(25):731.

23. Ameur A, Dahlberg J, Olason P, et al. SweGen: a whole-genome data resource of genetic variability in a cross-section of the Swedish population. Eur J Hum Genet. 2017;25(11):1253-60.
24. Karczewski KJ, Francioli LC, Tiao G, et al. Variation across 141,456 human exomes and genomes reveals the spectrum of loss-of-function intolerance across human protein-coding genes. bioRxiv. 2019:531210.
25. O'Leary NA, Wright MW, Brister JR, et al. Reference sequence (RefSeq) database at NCBI: current status, taxonomic expansion, and functional annotation. Nucleic Acids Res. 2016;44(D1):D733-45.
26. Kanehisa M, Goto S. KEGG: kyoto encyclopedia of genes and genomes. Nucleic Acids Res. 2000;28(1):27-30.
27. Rönnblom L, Leonard D. Interferon pathway in SLE: one key to unlocking the mystery of the disease. Lupus Sci Med. 2019;6(1):e000270.
28. Rodero MP, Crow YJ. Type I interferon-mediated monogenic autoinflammation: The type I interferonopathies, a conceptual overview. The Journal of experimental medicine. 2016;213(12):2527-38.
29. Davidson S, Steiner A, Harapas CR, et al. An Update on Autoinflammatory Diseases: Interferonopathies. Current rheumatology reports. 2018;20(7):38.
30. Chen L, Morris DL, Vyse TJ. Genetic advances in systemic lupus erythematosus: an update. Curr Opin Rheumatol. 2017;29(5):423-33.
31. Tsokos GC, Lo MS, Costa Reis P, et al. New insights into the immunopathogenesis of systemic lupus erythematosus. Nat Rev Rheumatol. 2016;12(12):716-30.
32. Lee S, Emond MJ, Bamshad MJ, et al. Optimal unified approach for rare-variant association testing with application to small-sample case-control whole-exome sequencing studies. Am J Hum Genet. 2012;91(2):224-37.
33. Mossotto E, Ashton JJ, O'Gorman L, et al. GenePy - a score for estimating gene pathogenicity in individuals using next-generation sequencing data. BMC Bioinformatics. 2019;20(1):254.
34. Wang K, Li M, Hakonarson H. ANNOVAR: functional annotation of genetic variants from high-throughput sequencing data. Nucleic Acids Res. 2010;38(16):e164.
35. Ioannidis NM, Rothstein JH, Pejaver V, et al. REVEL: An Ensemble Method for Predicting the Pathogenicity of Rare Missense Variants. Am J Hum Genet. 2016;99(4):877-85.
36. Rentzsch P, Witten D, Cooper GM, et al. CADD: predicting the deleteriousness of variants throughout the human genome. Nucleic Acids Res. 2019;47(D1):D886-D94.
37. Charrad M. GN, Boiteau V., Niknafs A. NbClust: An R Package for Determining the Relevant Number of Clusters in a Data Set. J of Statistical Software. 2014;61(6):1-36.
38. Gu Z, Eils R, Schlesner M. Complex heatmaps reveal patterns and correlations in multidimensional genomic data. Bioinformatics. 2016;32(18):2847-9.
39. Thorlacius GE, Hultin-Rosenberg L, Sandling JK, et al. Genetic and clinical basis for two distinct subtypes of primary Sjogren's syndrome. Rheumatology (Oxford). 2020.
40. McCarthy S, Das S, Kretzschmar W, et al. A reference panel of 64,976 haplotypes for genotype imputation. Nature genetics. 2016;48(10):1279-83.
