# Supplementary material for: Molecular pathways in patients with systemic lupus erythematosus revealed by gene-centred DNA sequencing
Source: Ann Rheum Dis. 2020 Oct 9;80(1):109–17. doi: 10.1136/annrheumdis-2020-218636 (PMC7788061; doi:10.1136/annrheumdis-2020-218636)
Supplement: Supplementary data [file annrheumdis-2020-218636supp002.pdf]

Supplementary Table S1a. Patient characteristics Scandinavian SLE cohorts.

	Sweden	Norway	Denmark	P†	$P_{bonferroni}$‡
Number of patients	958	289	547		
Female	826 (86.2%)	258 (89.3%)	484 (89.6%)	0.036	0.54
Age at diagnosis	36 (3-85)	32 (9-73)	33 (3-80)	9.5E-07	1.4E-05
Age at follow-up	52 (18-94)	52 (23-90)	47 (18-80)	8.4E-06	0.00013
Deceased (all causes)*	74 (7.7%)	-	-	-	-
Deceased age	68 (27-91)	-	-	-	-
≥4 ACR criteria (Tan et al., 1982; Hochberg et al., 1997)	958 (100%)	289 (100%)	547 (100%)	1	1
Number of ACR criteria fulfilled	5.7 (4-10)	5.9 (4-9)	5.9 (4-11)	0.00094	0.014
ACR criteria:					
1) Malar rash	543 (56.7%)	171 (59.4%)	323 (59.0%)	0.29	1
2) Discoid rash	235 (24.5%)	36 (12.5%)	51 (9.3%)	3.9E-15	5.9E-14
3) Photosensitivity	648 (67.6%)	208 (73.5%)	307 (56.1%)	0.015	0.22
4) Oral Ulcers	241 (25.2%)	95 (33.8%)	160 (29.3%)	0.0081	0.12
5) Arthritis	754 (78.7%)	224 (77.8%)	459 (83.9%)	0.11	1
6) Serositis	409 (42.7%)	113 (39.1%)	224 (41.0%)	0.31	1
7) Renal disorder	338 (35.3%)	115 (40.2%)	239 (43.7%)	0.0019	0.028
8) Neurologic disorder	99 (10.3%)	23 (8.0%)	65 (11.9%)	0.94	1
9) Hematologic disorder	615 (64.2%)	206 (75.2%)	398 (72.8%)	2.1E-05	3.2E-04
10) Immunologic disorder	653 (68.2%)	227 (80.8%)	491 (89.9%)	< 2.2E-16	3.3E-15
11) Positive ANA	942 (98.3%)	288 (100%)	537 (98.2%)	0.44	1
Autoantibodies:					
Anti-DNA	449 (61.8%)	-	-	-	-
Anti-Sm	99 (13.6%)	-	-	-	-
Anti-RNP	188 (26.1%)	-	-	-	-
Anti-SSA	351 (37.3%)	-	-	-	-
Anti-SSB	206 (21.8%)	-	-	-	-
Anti-cardiolipin IgG	243 (25.9%)	-	-	-	-
Anti-beta2-glycoprotein I IgG	179 (19.8%)	-	-	-	-
Rheumatoid factor (RF)	130 (23.9%)	-	-	-	-
Lupus anticoagulant (LAC)	137 (22.6%)	-	-	-	-
SLICC Damage Index (Gladman et al., 1996)	2.2 (0-14)	-	-	-	-

Data are number (%) or mean (range) excluding patients with missing data.
ACR: the American College of Rheumatology
SLICC: The Systemic Lupus Erythematosus International Collaborating Clinics
*An extended follow-up point was also added for death at which a total of 157 (16.4%) patients were recorded as deceased at an mean age of 68 (27-96) years.
†Categorical variables were compared using a Fisher's exact test and continuous variables by Student's unpaired t-test. The Swedish patients were compared to the combined Norwegian and Danish cohort.
‡Bonferroni corrected P-value, corrected for 15 tests.

Supplementary Table S1b. Clinical information on lupus nephritis Swedish SLE cohort.

	Sweden (number of patients)
Renal disorder	338
Renal biopsy data	257
Biopsy confirmed nephritis*	245
Class I-II	33
Class III-IV	153
Class V	41
Other	18
Dialysis or transplantation (ESRD)	35

*Biopsies were classified according to the WHO or the ISN/RPS 2003 classification systems.
ESRD: End-Stage Renal Disease

Supplementary Table S2. 35 analysed KEGG pathways and their genes. Each pathway included at least five genes and half or more of genes needed to be included in the targeted sequencing effort. Only sequenced genes are listed.

Pathway (KEGG ID)	Genes (RefSeq)
Adherens junction (hsa04520)	*ACTN1, ACTN4, AFDN, CDC42, CDH1, CREBBP, CTNNA1, CTNNB1, CTNND1, EGFR, EP300, ERBB2, FER, FYN, INSR, IQGAP1, LMO7, MAP3K7, MAPK1, MAPK3, MET, NECTIN2, PTPN1, PTPN6, PTPRM, RAC1, RAC2, RAC3, RHOA, SMAD3, SMAD4, SNAI1, SORBS1, SRC, TCF7, TGFBR1, TGFBR2, VCL, WAS, WASL, YES1*
Antigen processing and presentation (hsa04612)	*B2M, CALR, CANX, CD4, CD74, CD8A, CD8B, CIITA, CREB1, HLA-A, HLA-B, HLA-C, HLA-DOA, HLA-DOB, HLA-DPA1, HLA-DPB1, HLA-DQA1, HLA-DQA2, HLA-DQB1, HLA-DRA, HLA-DRB1, HLA-DRB5, HLA-E, HSPA6, HSPA8, IFNG, KIR2DL1, KIR2DL3, KLRC1, KLRC2, KLRC3, KLRD1, PDIA3, RFX5, RFXANK, RFXAP, TAP1, TAP2, TAPBP, TNF*
Apoptosis - multiple species (hsa04215)	*APAF1, BAK1, BAX, BCL2, BCL2L1, BCL2L11, BID, BIRC2, BIRC3, BIRC6, CASP3, CASP8, CASP9, CYCS, FADD, MAPK10, MAPK8, MAPK9, NGFR, TNFRSF1A*
Apoptosis (hsa04210)	*AIFM1, AKT1, APAF1, ATM, BAD, BAK1, BAX, BCL2, BCL2A1, BCL2L1, BCL2L11, BID, BIRC2, BIRC3, CAPN1, CAPN2, CASP10, CASP2, CASP3, CASP8, CASP9, CFLAR, CHUK, CSF2RB, CTSH, CTSK, CYCS, DAXX, DDIT3, DFFA, EIF2S1, ENDOG, FADD, FAS, FASLG, FOS, GADD45B, GADD45G, GZMB, HRAS, IKBKB, IKBKG, IL3, ITPR1, JUN, KRAS, MAP2K1, MAP2K2, MAP3K14, MAP3K5, MAPK1, MAPK10, MAPK3, MAPK8, MAPK9, NFKB1, NFKBIA, NGF, NRAS, NTRK1, PARP1, PIK3CA, PIK3CB, PIK3CD, PRF1, RAF1, RELA, RIPK1, TNF, TNFRSF1A, TNFSF10, TP53, TRADD, TRAF1, TRAF2*
B cell receptor signaling pathway (hsa04662)	*AKT1, BCL10, BLNK, BTK, CARD11, CD19, CD22, CD79A, CD79B, CD81, CHUK, CR2, DAPP1, FCGR2B, FOS, GRB2, GSK3B, HRAS, IFITM1, IKBKB, IKBKG, INPP5D, JUN, KRAS, LILRB1, LILRB4, LYN, MALT1, MAP2K1, MAP2K2, MAPK1, MAPK3, NFATC1, NFATC2, NFATC3, NFKB1, NFKBIA, NFKBIB, NRAS, PIK3CA, PIK3CB, PIK3CD, PLCG2, PPP3CA, PPP3CB, PPP3CC, PPP3R1, PPP3R2, PRKCB, PTPN6, RAC1, RAC2, RAC3, RAF1, RASGRP3, RELA, SOS1, SYK, VAV1, VAV2*
Chemokine signaling pathway (hsa04062)	*ADCY2, ADCY3, AKT1, BAD, BRAF, CCL1, CCL11, CCL13, CCL19, CCL2, CCL21, CCL24, CCL25, CCL26, CCL3, CCL3L3, CCL4, CCL5, CCL7, CCR1, CCR10, CCR2, CCR3, CCR4, CCR5, CCR6, CCR7, CCR8, CCR9, CDC42, CHUK, CRK, CRKL, CX3CL1, CX3CR1, CXCL1, CXCL10, CXCL11, CXCL12, CXCL13, CXCL14, CXCL16, CXCL2, CXCL3, CXCL5, CXCL8, CXCL9, CXCR1, CXCR2, CXCR3, CXCR4, CXCR5, CXCR6, ELMO1, FGR, FOXO3, GRB2, GSK3B, HCK, HRAS, IKBKB, IKBKG, ITK, JAK2, JAK3, KRAS, LYN, MAP2K1, MAPK1, MAPK3, NCF1, NFKB1, NFKBIA, NFKBIB, NRAS, PAK1, PF4, PIK3CA, PIK3CB, PIK3CD, PIK3CG, PLCB2, PPBP, PREX1, PRKCB, PRKCD, PRKCZ, PTK2, PTK2B, PXN, RAC1, RAC2, RAC3, RAF1, RELA, RHOA, ROCK1, ROCK2, SHC1, SOS1, SRC, STAT1, STAT2, STAT3, STAT5B, VAV1, VAV2, WAS, WASL, XCR1*
Complement and coagulation cascades (hsa04610)	*A2M, C1QA, C1QB, C1QC, C1R, C1S, C2, C3, C3AR1, C4A, C4B, C4BPA, C5, C5AR1, C6, C7, C9, CD46, CD55, CD59, CFB, CFD, CFH, CFI, CR1, CR2, F11, F12, F2, F2R, F8, FGA, FGB, FGG, ITGAM, ITGAX, ITGB2, KLKB1, MASP1, MASP2, MBL2, PLAT, PLAU, PLG, PROS1, SERPINA1, SERPIND1, SERPINE1, SERPING1, VWF*
C-type lectin receptor signaling pathway (hsa04625)	*AKT1, ARHGEF12, BCL10, BCL3, CARD9, CASP1, CASP8, CBLB, CD209, CHUK, CLEC7A, CYLD, FCER1G, HRAS, IKBKB, IKBKE, IKBKG, IL10, IL12A, IL12B, IL1B, IL2, IL23A, IL6, IRF1, IRF9, ITPR1, JUN, KRAS, LSP1, MALT1, MAP3K14, MAPK1, MAPK10, MAPK11, MAPK12, MAPK13, MAPK14, MAPK3, MAPK8, MAPK9, MAPKAPK2, MDM2, NFATC1, NFATC2, NFATC3, NFATC4, NFKB1, NFKB2, NFKBIA, NLRP3, NRAS, PAK1, PIK3CA, PIK3CB, PIK3CD, PLCG2, PPP3CA, PPP3CB, PPP3CC, PPP3R1, PPP3R2, PRKCD, PTGS2, PTPN11, PYCARD, RAF1, RELA, RELB, RHOA, SRC, STAT1, STAT2, SYK, TNF*
Cytokine-cytokine receptor interaction (hsa04060)	*ACKR3, ACVR1, ACVR1B, ACVR2A, ACVR2B, AMH, AMHR2, BMPR1A, BMPR1B, BMPR2, CCL1, CCL11, CCL13, CCL19, CCL2, CCL21, CCL24, CCL25, CCL26, CCL3, CCL3L3, CCL4, CCL5, CCL7, CCR1, CCR10, CCR2, CCR3, CCR4, CCR5, CCR6, CCR7, CCR8, CCR9, CD27, CD4, CD40, CD40LG, CD70, CLCF1, CNTF, CNTFR, CSF1, CSF1R, CSF2, CSF2RB, CSF3, CSF3R, CTF1, CX3CL1, CX3CR1, CXCL1, CXCL10, CXCL11, CXCL12, CXCL13, CXCL14, CXCL16, CXCL2, CXCL3, CXCL5, CXCL8, CXCL9, CXCR1, CXCR2, CXCR3, CXCR4, CXCR5, CXCR6, EDA, EDA2R, EDAR, EPO, EPOR, FAS, FASLG, GH1, GHR, IFNA1, IFNA10, IFNA13, IFNA14, IFNA16, IFNA17, IFNA2, IFNA21, IFNA4, IFNA5, IFNA6, IFNA7, IFNA8, IFNAR1, IFNAR2, IFNB1, IFNE, IFNG, IFNGR1, IFNGR2, IFNK, IFNL1, IFNLR1, IFNW1, IL10, IL10RA, IL10RB, IL11, IL11RA, IL12A, IL12B, IL12RB1, IL12RB2, IL13, IL13RA1, IL13RA2, IL15, IL15RA, IL17A, IL17B, IL17F, IL17RA, IL17RB, IL17RC, IL18, IL18R1, IL18RAP, IL19, IL1A, IL1B, IL1R1, IL1R2, IL1RAP, IL1RL1, IL1RL2, IL1RN, IL2, IL20, IL20RA, IL20RB, IL21, IL21R, IL22, IL22RA1, IL23A, IL23R, IL24, IL25, IL26, IL27, IL27RA, IL2RA, IL2RB, IL2RG, IL3, IL32, IL4, IL4R, IL5, IL5RA, IL6, IL6R, IL6ST, IL7, IL7R, IL9, LEP, LEPR, LIF, LIFR, LTA, LTB, LTBR, MPL, NGF, NGFR, OSM, OSMR, PF4, PPBP, PRL, PRLR, RELT, TGFB1, TGFB2, TGFB3, TGFBR1, TGFBR2, TNF, TNFRSF11A, TNFRSF11B, TNFRSF12A, TNFRSF13B, TNFRSF13C, TNFRSF14, TNFRSF17, TNFRSF18, TNFRSF19, TNFRSF1A, TNFRSF1B, TNFRSF21, TNFRSF25, TNFRSF4, TNFRSF6B, TNFRSF8, TNFRSF9, TNFSF10, TNFSF11, TNFSF12, TNFSF13, TNFSF13B, TNFSF14, TNFSF15, TNFSF18, TNFSF4, TNFSF8, TNFSF9, TSLP, XCR1*

Cytosolic DNA-sensing pathway (hsa04623)	*ADAR, AIM2, CASP1, CCL4, CCL5, CHUK, CXCL10, DDX58, IFNA1, IFNA10, IFNA13, IFNA14, IFNA16, IFNA17, IFNA2, IFNA21, IFNA4, IFNA5, IFNA6, IFNA7, IFNA8, IFNB1, IKBKB, IKBKE, IKBKG, IL18, IL1B, IL6, IRF3, IRF7, MAVS, NFKB1, NFKBIA, NFKBIB, PYCARD, RELA, RIPK1, TBK1, TREX1, ZBP1*
ErbB signaling pathway (hsa04012)	*ABL1, AKT1, BAD, BRAF, CBL, CBLB, CDKN1B, CRK, CRKL, EGF, EGFR, EIF4EBP1, ELK1, ERBB2, ERBB3, GAB1, GRB2, GSK3B, HBEGF, HRAS, JUN, KRAS, MAP2K1, MAP2K2, MAP2K4, MAP2K7, MAPK1, MAPK10, MAPK3, MAPK8, MAPK9, MTOR, MYC, NCK1, NRAS, NRG1, PAK1, PAK2, PIK3CA, PIK3CB, PIK3CD, PLCG1, PLCG2, PRKCA, PRKCB, PTK2, RAF1, RPS6KB1, SHC1, SOS1, SRC, STAT5A, STAT5B, TGFA*
Fc epsilon RI signaling pathway (hsa04664)	*AKT1, BTK, CSF2, FCER1A, FCER1G, FYN, GAB2, GRB2, HRAS, IL13, IL3, IL4, IL5, INPP5D, KRAS, LAT, LCP2, LYN, MAP2K1, MAP2K2, MAP2K3, MAP2K4, MAP2K6, MAP2K7, MAPK1, MAPK10, MAPK11, MAPK12, MAPK13, MAPK14, MAPK3, MAPK8, MAPK9, MS4A2, NRAS, PIK3CA, PIK3CB, PIK3CD, PLCG1, PLCG2, PRKCA, RAC1, RAC2, RAC3, RAF1, SOS1, SYK, TNF, VAV1, VAV2*
Fc gamma R-mediated phagocytosis (hsa04666)	*AKT1, AMPH, ARF6, ASAP1, ASAP2, CDC42, CFL1, CRK, CRKL, DNM2, FCGR1A, FCGR2A, FCGR2B, FCGR3B, GAB2, GSN, HCK, INPP5D, LAT, LYN, MAP2K1, MAPK1, MAPK3, NCF1, PAK1, PIK3CA, PIK3CB, PIK3CD, PIP5K1A, PIP5K1C, PLA2G6, PLCG1, PLCG2, PRKCA, PRKCB, PRKCD, PRKCE, PTPRC, RAC1, RAC2, RAF1, RPS6KB1, SYK, VASP, VAV1, VAV2, WAS, WASL*
FoxO signaling pathway (hsa04068)	*AKT1, ATM, BCL2L11, BCL6, BRAF, CAT, CCND1, CDK2, CDKN1B, CDKN2B, CHUK, CREBBP, EGF, EGFR, EP300, FASLG, FOXG1, FOXO1, FOXO3, GADD45B, GADD45G, GRB2, HOMER2, HOMER3, HRAS, IGF1, IKBKB, IL10, IL6, IL7R, INS, INSR, IRS1, KRAS, MAP2K1, MAP2K2, MAPK1, MAPK10, MAPK11, MAPK12, MAPK13, MAPK14, MAPK3, MAPK8, MAPK9, MDM2, NRAS, PIK3CA, PIK3CB, PIK3CD, PTEN, RAF1, RAG1, RAG2, SMAD3, SMAD4, SOD2, SOS1, STAT3, TGFB1, TGFB2, TGFB3, TGFBR1, TGFBR2, TNFSF10*
Hematopoietic cell lineage (hsa04640)	*CD14, CD19, CD1A, CD1B, CD1C, CD1D, CD22, CD36, CD38, CD3D, CD3E, CD4, CD44, CD5, CD55, CD59, CD8A, CD8B, CR1, CR2, CSF1, CSF1R, CSF2, CSF3, CSF3R, DNTT, EPO, EPOR, FCER2, FCGR1A, FLT3, FLT3LG, GYPA, HLA-DOA, HLA-DOB, HLA-DPA1, HLA-DPB1, HLA-DQA1, HLA-DQA2, HLA-DQB1, HLA-DRA, HLA-DRB1, HLA-DRB5, IL11, IL11RA, IL1A, IL1B, IL1R1, IL1R2, IL2RA, IL3, IL4, IL4R, IL5, IL5RA, IL6, IL6R, IL7, IL7R, ITGA2, ITGA3, ITGA4, ITGA5, ITGAM, KIT, KITLG, TFRC, TNF*
HIF-1 signaling pathway (hsa04066)	*AKT1, ANGPT1, ANGPT2, ARNT, BCL2, CDKN1B, CREBBP, CYBB, EGF, EGFR, EIF4E, EIF4EBP1, ENO1, ENO2, ENO3, EP300, EPO, ERBB2, FLT1, HIF1A, HMOX1, IFNG, IFNGR1, IFNGR2, IGF1, IL6, IL6R, INS, INSR, LTBR, MAP2K1, MAP2K2, MAPK1, MAPK3, MTOR, NFKB1, NOS2, PDHB, PDK1, PFKL, PFKM, PGK1, PIK3CA, PIK3CB, PIK3CD, PLCG1, PLCG2, PRKCA, PRKCB, RELA, RPS6KB1, SERPINE1, SLC2A1, STAT3, TEK, TF, TFRC, TIMP1, TLR4, VEGFA*
IL-17 signaling pathway (hsa04657)	*CASP3, CASP8, CCL11, CCL2, CCL7, CEBPB, CHUK, CSF2, CSF3, CXCL1, CXCL10, CXCL2, CXCL3, CXCL5, CXCL8, DEFB4A, FADD, FOS, GSK3B, IFNG, IKBKB, IKBKE, IKBKG, IL13, IL17A, IL17B, IL17F, IL17RA, IL17RB, IL17RC, IL1B, IL25, IL4, IL5, IL6, JUN, JUND, MAP3K7, MAPK1, MAPK10, MAPK11, MAPK12, MAPK13, MAPK14, MAPK3, MAPK7, MAPK8, MAPK9, MMP1, MMP13, MMP3, MMP9, MUC5B, NFKB1, NFKBIA, PTGS2, RELA, TAB2, TAB3, TBK1, TNF, TNFAIP3, TRADD, TRAF2, TRAF3, TRAF3IP2, TRAF5, TRAF6*
Intestinal immune network for IgA production (hsa04672)	*AICDA, CCL25, CCR10, CCR9, CD28, CD40, CD40LG, CD80, CD86, CXCL12, CXCR4, HLA-DOA, HLA-DOB, HLA-DPA1, HLA-DPB1, HLA-DQA1, HLA-DQA2, HLA-DQB1, HLA-DRA, HLA-DRB1, HLA-DRB5, ICOS, ICOSLG, IL10, IL15, IL15RA, IL2, IL4, IL5, IL6, ITGA4, LTBR, MAP3K14, TGFB1, TNFRSF13B, TNFRSF13C, TNFRSF17, TNFSF13, TNFSF13B*
JAK-STAT signaling pathway (hsa04630)	*AKT1, BCL2, BCL2L1, CCND1, CISH, CNTF, CNTFR, CREBBP, CSF2, CSF2RB, CSF3, CSF3R, CTF1, EGF, EGFR, EP300, EPO, EPOR, GH1, GHR, GRB2, HRAS, IFNA1, IFNA10, IFNA13, IFNA14, IFNA16, IFNA17, IFNA2, IFNA21, IFNA4, IFNA5, IFNA6, IFNA7, IFNA8, IFNAR1, IFNAR2, IFNB1, IFNE, IFNG, IFNGR1, IFNGR2, IFNK, IFNL1, IFNLR1, IFNW1, IL10, IL10RA, IL10RB, IL11, IL11RA, IL12A, IL12B, IL12RB1, IL12RB2, IL13, IL13RA1, IL13RA2, IL15, IL15RA, IL19, IL2, IL20, IL20RA, IL20RB, IL21, IL21R, IL22, IL22RA1, IL22RA2, IL23A, IL23R, IL24, IL27RA, IL2RA, IL2RB, IL2RG, IL3, IL4, IL4R, IL5, IL5RA, IL6, IL6R, IL6ST, IL7, IL7R, IL9, IRF9, JAK1, JAK2, JAK3, LEP, LEPR, LIF, LIFR, MPL, MTOR, MYC, OSM, OSMR, PDGFRA, PDGFRB, PIAS1, PIK3CA, PIK3CB, PIK3CD, PIM1, PRL, PRLR, PTPN11, PTPN2, PTPN6, RAF1, SOCS1, SOCS2, SOCS3, SOCS6, SOS1, STAM, STAT1, STAT2, STAT3, STAT4, STAT5A, STAT5B, STAT6, TSLP, TYK2*
Leukocyte transendothelial migration (hsa04670)	*ACTN1, ACTN4, AFDN, CDC42, CDH5, CLDN1, CLDN19, CLDN5, CTNNA1, CTNNB1, CTNND1, CXCL12, CXCR4, CYBA, CYBB, EZR, F11R, ICAM1, ITGA4, ITGAL, ITGAM, ITGB1, ITGB2, ITK, JAM2, JAM3, MAPK11, MAPK12, MAPK13, MAPK14, MMP2, MMP9, NCF1, NCF2, NCF4, PECAM1, PIK3CA, PIK3CB, PIK3CD, PLCG1, PLCG2, PRKCA, PRKCB, PTK2, PTK2B, PTPN11, PXN, RAC1, RAC2, RASSF5, RHOA, RHOH, ROCK1, ROCK2, SIPA1, THY1, VASP, VAV1, VAV2, VCAM1, VCL*

MAPK signaling pathway (hsa04010)	*AKT1, ANGPT1, ANGPT2, ATF2, BRAF, CACNA1H, CACNA1S, CACNA2D2, CASP3, CD14, CDC42, CHUK, CRK, CRKL, CSF1, CSF1R, DAXX, DDIT3, DUSP1, ECSIT, EGF, EGFR, ELK1, ERBB2, ERBB3, FAS, FASLG, FGF10, FGF7, FLNB, FLT1, FLT3, FLT3LG, FLT4, FOS, GADD45B, GADD45G, GNA12, GRB2, HGF, HRAS, HSPA6, HSPA8, IGF1, IKBKB, IKBKG, IL1A, IL1B, IL1R1, IL1RAP, INS, INSR, IRAK1, IRAK4, JUNE, JUND, KDR, KIT, KITLG, KRAS, LAMTOR3, MAP2K1, MAP2K2, MAP2K3, MAP2K4, MAP2K5, MAP2K6, MAP2K7, MAP3K1, MAP3K14, MAP3K2, MAP3K3, MAP3K4, MAP3K5, MAP3K6, MAP3K7, MAP3K8, MAP4K1, MAP4K3, MAPK1, MAPK10, MAPK11, MAPK12, MAPK13, MAPK14, MAPK3, MAPK7, MAPK8, MAPK9, MAPKAPK2, MAPKAPK5, MAX, MET, MYC, MYD88, NFATC1, NFATC3, NFKB1, NFKB2, NGF, NGFR, NR4A1, NRAS, NTF3, NTRK1, PAK1, PAK2, PDGFRA, PDGFRB, PPP3CA, PPP3CB, PPP3CC, PPP3R1, PPP3R2, PRKCA, PRKCB, PTPN7, RAC1, RAC2, RAC3, RAF1, RASA1, RASGRP1, RASGRP3, RELA, RELB, RPS6KA1, RPS6KA4, SOS1, SRF, TAB1, TAB2, TEK, TGFA, TGFB1, TGFB2, TGFB3, TGFBR1, TGFBR2, TNF, TNFRSF1A, TP53, TRADD, TRAF2, TRAF6, VEGFA, VEGFB, VEGFC, VEGFD*
Natural killer cell mediated cytotoxicity (hsa04650)	*BID, BRAF, CASP3, CD244, CD247, CD48, CSF2, FAS, FASLG, FCER1G, FCGR3B, FYN, GRB2, GZMB, HCST, HLA-A, HLA-B, HLA-C, HLA-E, HRAS, ICAM1, IFNA1, IFNA10, IFNA13, IFNA14, IFNA16, IFNA17, IFNA2, IFNA21, IFNA4, IFNA5, IFNA6, IFNA7, IFNA8, IFNAR1, IFNAR2, IFNB1, IFNG, IFNGR1, IFNGR2, ITGAL, ITGB2, KIR2DL1, KIR2DL3, KLRC1, KLRC2, KLRC3, KLRD1, KLRK1, KRAS, LAT, LCK, LCP2, MAP2K1, MAP2K2, MAPK1, MAPK3, MICA, MICB, NCR1, NCR2, NCR3, NFATC1, NFATC2, NRAS, PAK1, PIK3CA, PIK3CB, PIK3CD, PLCG1, PLCG2, PPP3CA, PPP3CB, PPP3CC, PPP3R1, PPP3R2, PRF1, PRKCA, PRKCB, PTK2B, PTPN11, PTPN6, RAC1, RAC2, RAC3, RAET1L, RAF1, SH2D1A, SH2D1B, SH3BP2, SHC1, SOS1, SYK, TNF, TNFSF10, TYROBP, ULBP3, VAV1, VAV2, ZAP70*
Neurotrophin signaling pathway (hsa04722)	*ABL1, AKT1, BAD, BAX, BCL2, BRAF, CDC42, CRK, CRKL, FASLG, FOXO3, GAB1, GRB2, GSK3B, HRAS, IKBKB, IRAK1, IRAK2, IRAK3, IRAK4, IRS1, JUN, KRAS, MAP2K1, MAP2K2, MAP2K5, MAP2K7, MAP3K1, MAP3K3, MAP3K5, MAPK1, MAPK10, MAPK11, MAPK12, MAPK13, MAPK14, MAPK3, MAPK7, MAPK8, MAPK9, MAPKAPK2, NFKB1, NFKBIA, NFKBIB, NGF, NGFR, NRAS, NTF3, NTRK1, PIK3CA, PIK3CB, PIK3CD, PLCG1, PLCG2, PRKCD, PTPN11, RAC1, RAF1, RAPGEF1, RELA, RHOA, RIPK2, RPS6KA1, SH2B1, SH2B3, SHC1, SOS1, TP53, TRAF6*
NF-kappa B signaling pathway (hsa04064)	*ATM, BCL10, BCL2, BCL2A1, BCL2L1, BIRC2, BIRC3, BLNK, BTK, CARD10, CARD11, CCL13, CCL19, CCL21, CCL4, CD14, CD40, CD40LG, CFLAR, CHUK, CXCL1, CXCL12, CXCL2, CXCL3, CXCL8, CYLD, DDX58, EDA, EDA2R, EDAR, GADD45B, ICAM1, IKBKB, IKBKG, IL1B, IL1R1, IRAK1, IRAK4, LAT, LBP, LCK, LTA, LTB, LTBR, LY96, LYN, MALT1, MAP3K14, MAP3K7, MYD88, NFKB1, NFKB2, NFKBIA, PARP1, PLAU, PLCG1, PLCG2, PRKCB, PRKCQ, PTGS2, RELA, RELB, RIPK1, SYK, TAB1, TAB2, TAB3, TICAM1, TICAM2, TIRAP, TLR4, TNF, TNFAIP3, TNFRSF11A, TNFRSF13C, TNFRSF1A, TNFSF11, TNFSF13B, TNFSF14, TRADD, TRAF1, TRAF2, TRAF3, TRAF5, TRAF6, TRIM25, VCAM1, ZAP70*
NOD-like receptor signaling pathway (hsa04621)	*AIM2, ANTXR2, ATG16L1, BCL2, BCL2L1, BIRC2, BIRC3, CARD9, CASP1, CASP5, CASP8, CASR, CCL2, CCL5, CHUK, CXCL1, CXCL2, CXCL3, CXCL8, CYBA, CYBB, DEFA1, DEFB4A, DNM1L, FADD, IFI16, IFNA1, IFNA10, IFNA13, IFNA14, IFNA16, IFNA17, IFNA2, IFNA21, IFNA4, IFNA5, IFNA6, IFNA7, IFNA8, IFNAR1, IFNAR2, IFNB1, IKBKB, IKBKE, IKBKG, IL18, IL1B, IL6, IRAK4, IRF3, IRF7, IRF9, ITPR1, JAK1, JUN, MAP3K7, MAPK1, MAPK10, MAPK11, MAPK12, MAPK13, MAPK14, MAPK3, MAPK8, MAPK9, MAVS, MEFV, MYD88, NAIP, NFKB1, NFKBIA, NFKBIB, NLRC4, NLRP1, NLRP12, NLRP3, NLRP7, NLRX1, NOD1, NOD2, OAS1, OAS2, PLCB2, PRKCD, PSTPIP1, PYCARD, RELA, RHOA, RIPK1, RIPK2, RNASEL, STAT1, STAT2, TAB1, TAB2, TAB3, TANK, TBK1, TICAM1, TLR4, TNF, TNFAIP3, TRAF2, TRAF3, TRAF5, TRAF6, TRPM7, TXN, TYK2*
Osteoclast differentiation (hsa04380)	*ACP5, AKT1, BLNK, BTK, CHUK, CREB1, CSF1, CSF1R, CTSK, CYBA, CYLD, FCGR1A, FCGR2A, FCGR2B, FCGR3B, FOS, FOSL2, FYN, GAB2, GRB2, IFNAR1, IFNAR2, IFNB1, IFNG, IFNGR1, IFNGR2, IKBKB, IKBKG, IL1A, IL1B, IL1R1, IRF9, JAK1, JUN, JUNB, JUND, LCK, LCP2, LILRB1, LILRB4, MAP2K1, MAP2K6, MAP2K7, MAP3K14, MAP3K7, MAPK1, MAPK10, MAPK11, MAPK12, MAPK13, MAPK14, MAPK3, MAPK8, MAPK9, MITF, NCF1, NCF2, NCF4, NFATC1, NFATC2, NFKB1, NFKB2, NFKBIA, NOX1, OSCAR, PIK3CA, PIK3CB, PIK3CD, PLCG2, PPARG, PPP3CA, PPP3CB, PPP3CC, PPP3R1, PPP3R2, RAC1, RELA, RELB, SIRPA, SOCS1, SOCS3, SPI1, STAT1, STAT2, SYK, TAB1, TAB2, TGFB1, TGFB2, TGFBR1, TGFBR2, TNF, TNFRSF11A, TNFRSF11B, TNFRSF1A, TNFSF11, TRAF2, TRAF6, TREM2, TYK2, TYROBP*
Prolactin signaling pathway (hsa04917)	*AKT1, CCND1, CGA, CISH, CSN2, CYP17A1, ESR1, ESR2, FOS, FOXO3, GRB2, GSK3B, HRAS, INS, IRF1, JAK2, KRAS, MAP2K1, MAP2K2, MAPK1, MAPK10, MAPK11, MAPK12, MAPK13, MAPK14, MAPK3, MAPK8, MAPK9, NFKB1, NRAS, PIK3CA, PIK3CB, PIK3CD, PRL, PRLR, RAF1, RELA, SHC1, SOCS1, SOCS2, SOCS3, SOCS6, SOS1, SRC, STAT1, STAT3, STAT5A, STAT5B, TH, TNFRSF11A, TNFSF11*
RIG-I-like receptor signaling pathway (hsa04622)	*AZI2, CASP10, CASP8, CHUK, CXCL10, CXCL8, CYLD, DDX58, DHX58, FADD, IFIH1, IFNA1, IFNA10, IFNA13, IFNA14, IFNA16, IFNA17, IFNA2, IFNA21, IFNA4, IFNA5, IFNA6, IFNA7, IFNA8, IFNB1, IFNE, IFNK, IFNW1, IKBKB, IKBKE, IKBKG, IL12A, IL12B, IRF3, IRF7, ISG15, MAP3K1, MAP3K7, MAPK10, MAPK11, MAPK12, MAPK13, MAPK14, MAPK8, MAPK9, MAVS, NFKB1, NFKBIA, NFKBIB, NLRX1, PIN1, RELA, RIPK1, SIKE1, TANK, TBK1, TBKBP1, TNF, TRADD, TRAF2, TRAF3, TRAF6, TRIM25*

T cell receptor signaling pathway (hsa04660)	*AKT1, BCL10, CARD11, CBLB, CD247, CD28, CD3D, CD3E, CD4, CD40LG, CD8A, CD8B, CDC42, CDK4, CHUK, CSF2, CTLA4, FOS, FYN, GRAP2, GRB2, GSK3B, HRAS, ICOS, IFNG, IKBKB, IKBKG, IL10, IL2, IL4, IL5, ITK, JUN, KRAS, LAT, LCK, LCP2, MALT1, MAP2K1, MAP2K2, MAP2K7, MAP3K14, MAP3K7, MAP3K8, MAPK1, MAPK10, MAPK11, MAPK12, MAPK13, MAPK14, MAPK3, MAPK8, MAPK9, NCK1, NFATC1, NFATC2, NFATC3, NFKB1, NFKBIA, NFKBIB, NRAS, PAK1, PAK2, PIK3CA, PIK3CB, PIK3CD, PLCG1, PPP3CA, PPP3CB, PPP3CC, PPP3R1, PPP3R2, PRKCQ, PTPN6, PTPRC, RAF1, RASGRP1, RELA, RHOA, SOS1, TNF, VAV1, VAV2, ZAP70*
Th1 and Th2 cell differentiation (hsa04658)	*CD247, CD3D, CD3E, CD4, CHUK, FOS, GATA3, HLA-DOA, HLA-DOB, HLA-DPA1, HLA-DPB1, HLA-DQA1, HLA-DQA2, HLA-DQB1, HLA-DRA, HLA-DRB1, HLA-DRB5, IFNG, IFNGR1, IFNGR2, IKBKB, IKBKG, IL12A, IL12B, IL12RB1, IL12RB2, IL13, IL2, IL2RA, IL2RB, IL2RG, IL4, IL4R, IL5, JAK1, JAK2, JAK3, JUN, LAT, LCK, MAF, MAML1, MAPK1, MAPK10, MAPK11, MAPK12, MAPK13, MAPK14, MAPK3, MAPK8, MAPK9, NFATC1, NFATC2, NFATC3, NFKB1, NFKBIA, NFKBIB, NOTCH1, NOTCH2, NOTCH3, PLCG1, PPP3CA, PPP3CB, PPP3CC, PPP3R1, PPP3R2, PRKCQ, RBPJ, RELA, RUNX3, STAT1, STAT4, STAT5A, STAT5B, STAT6, TBX21, TYK2, ZAP70*
Th17 cell differentiation (hsa04659)	*AHR, CD247, CD3D, CD3E, CD4, CHUK, FOS, FOXP3, GATA3, HIF1A, HLA-DOA, HLA-DOB, HLA-DPA1, HLA-DPB1, HLA-DQA1, HLA-DQA2, HLA-DQB1, HLA-DRA, HLA-DRB1, HLA-DRB5, IFNG, IFNGR1, IFNGR2, IKBKB, IKBKG, IL12RB1, IL17A, IL17F, IL1B, IL1R1, IL1RAP, IL2, IL21, IL21R, IL22, IL23A, IL23R, IL27RA, IL2RA, IL2RB, IL2RG, IL4, IL4R, IL6, IL6R, IL6ST, IRF4, JAK1, JAK2, JAK3, JUN, LAT, LCK, MAPK1, MAPK10, MAPK11, MAPK12, MAPK13, MAPK14, MAPK3, MAPK8, MAPK9, MTOR, NFATC1, NFATC2, NFATC3, NFKB1, NFKBIA, NFKBIB, PLCG1, PPP3CA, PPP3CB, PPP3CC, PPP3R1, PPP3R2, PRKCQ, RARA, RELA, RORA, RORC, RUNX1, RXRA, SMAD2, SMAD4, STAT1, STAT3, STAT5A, STAT5B, STAT6, TBX21, TGFB1, TGFBR1, TGFBR2, TYK2, ZAP70*
TNF signaling pathway (hsa04668)	*AKT1, ATF2, BAG4, BCL3, BIRC2, BIRC3, CASP10, CASP3, CASP8, CCL2, CCL5, CEBPB, CFLAR, CHUK, CREB1, CSF1, CSF2, CX3CL1, CXCL1, CXCL10, CXCL2, CXCL3, CXCL5, DNM1L, FADD, FAS, FOS, ICAM1, IFNB1, IKBKB, IKBKG, IL15, IL18R1, IL1B, IL6, IRF1, JUN, JUNB, LIF, LTA, MAP2K1, MAP2K3, MAP2K4, MAP2K6, MAP2K7, MAP3K14, MAP3K5, MAP3K7, MAP3K8, MAPK1, MAPK10, MAPK11, MAPK12, MAPK13, MAPK14, MAPK3, MAPK8, MAPK9, MMP14, MMP3, MMP9, NFKB1, NFKBIA, NOD2, PIK3CA, PIK3CB, PIK3CD, PTGS2, RELA, RIPK1, RPS6KA4, SELE, SOCS3, TAB1, TAB2, TAB3, TNF, TNFAIP3, TNFRSF1A, TNFRSF1B, TRADD, TRAF1, TRAF2, TRAF3, TRAF5, VCAM1, VEGFC, VEGFD*
Toll-like receptor signaling pathway (hsa04620)	*AKT1, CASP8, CCL3, CCL3L3, CCL4, CCL5, CD14, CD40, CD80, CD86, CHUK, CTSK, CXCL10, CXCL11, CXCL8, CXCL9, FADD, FOS, IFNA1, IFNA10, IFNA13, IFNA14, IFNA16, IFNA17, IFNA2, IFNA21, IFNA4, IFNA5, IFNA6, IFNA7, IFNA8, IFNAR1, IFNAR2, IFNB1, IKBKB, IKBKE, IKBKG, IL12A, IL12B, IL1B, IL6, IRAK1, IRAK4, IRF3, IRF5, IRF7, JUN, LBP, LY96, MAP2K1, MAP2K2, MAP2K3, MAP2K4, MAP2K6, MAP2K7, MAP3K7, MAP3K8, MAPK1, MAPK10, MAPK11, MAPK12, MAPK13, MAPK14, MAPK3, MAPK8, MAPK9, MYD88, NFKB1, NFKBIA, PIK3CA, PIK3CB, PIK3CD, RAC1, RELA, RIPK1, SPP1, STAT1, TAB1, TAB2, TBK1, TICAM1, TICAM2, TIRAP, TLR1, TLR2, TLR3, TLR4, TLR5, TLR6, TLR7, TLR8, TLR9, TNF, TOLLIP, TRAF3, TRAF6*
VEGF signaling pathway (hsa04370)	*AKT1, BAD, CASP9, CDC42, HRAS, KDR, KRAS, MAP2K1, MAP2K2, MAPK1, MAPK11, MAPK12, MAPK13, MAPK14, MAPK3, MAPKAPK2, NFATC2, NRAS, PIK3CA, PIK3CB, PIK3CD, PLCG1, PLCG2, PPP3CA, PPP3CB, PPP3CC, PPP3R1, PPP3R2, PRKCA, PRKCB, PTGS2, PTK2, PXN, RAC1, RAC2, RAC3, RAF1, SRC, VEGFA*
Viral protein interaction with cytokine and cytokine receptor (hsa04061)	*ACKR3, CCL1, CCL11, CCL13, CCL19, CCL2, CCL21, CCL24, CCL25, CCL26, CCL3, CCL3L3, CCL4, CCL5, CCL7, CCR1, CCR10, CCR2, CCR3, CCR4, CCR5, CCR6, CCR7, CCR8, CCR9, CSF1, CSF1R, CX3CL1, CX3CR1, CXCL1, CXCL10, CXCL11, CXCL12, CXCL13, CXCL14, CXCL2, CXCL3, CXCL5, CXCL8, CXCL9, CXCR1, CXCR2, CXCR3, CXCR4, CXCR5, IL10, IL10RA, IL10RB, IL18, IL18R1, IL18RAP, IL19, IL2, IL20, IL20RA, IL20RB, IL22RA1, IL24, IL2RA, IL2RB, IL2RG, IL6, IL6R, IL6ST, LTA, LTBR, PF4, PPBP, TNF, TNFRSF14, TNFRSF1A, TNFRSF1B, TNFSF10, TNFSF14, XCR1*

Supplementary Table S3. SLE case-control pathway based aggregate association analysis result (SKAT-O).

Pathway (KEGG ID)	Genes in pathway	Genes on array	Ratio genes on array/ genes in pathway	SNVs in test	P-value	FDR
Th1 and Th2 cell differentiation (hsa04658)	92	78	0.85	14362	6.3E-11	2.2E-09
Antigen processing and presentation (hsa04612)	77	40	0.52	8017	1.8E-10	3.1E-09
Hematopoietic cell lineage (hsa04640)	97	71	0.73	13013	3.8E-10	4.5E-09
Th17 cell differentiation (hsa04659)	107	96	0.90	19347	1.7E-09	1.5E-08
Intestinal immune network for IgA production (hsa04672)	49	39	0.80	7909	3.4E-08	2.4E-07
Natural killer cell mediated cytotoxicity (hsa04650)	131	100	0.76	15821	4.7E-06	2.8E-05
TNF signaling pathway (hsa04668)	112	88	0.79	12639	1.9E-05	9.4E-05
JAK-STAT signaling pathway (hsa04630)	162	133	0.82	18003	7.4E-05	0.00032
RIG-I-like receptor signaling pathway (hsa04622)	70	63	0.90	8459	0.00021	0.00080
NOD-like receptor signaling pathway (hsa04621)	178	109	0.61	15729	0.00031	0.0011
Complement and coagulation cascades (hsa04610)	79	50	0.63	7112	0.00041	0.0013
Toll-like receptor signaling pathway (hsa04620)	104	96	0.92	12178	0.00080	0.0022
Cytokine-cytokine receptor interaction (hsa04060)	294	221	0.75	26771	0.00083	0.0022
C-type lectin receptor signaling pathway (hsa04625)	104	75	0.72	12986	0.0020	0.0050
IL-17 signaling pathway (hsa04657)	93	68	0.73	9358	0.0043	0.0100
Fc epsilon RI signaling pathway (hsa04664)	68	51	0.75	8514	0.0052	0.011
Viral protein interaction with cytokine and cytokine receptor (hsa04061)	100	75	0.75	8435	0.0062	0.013
NF-kappa B signaling pathway (hsa04064)	102	88	0.86	14349	0.0078	0.015
Osteoclast differentiation (hsa04380)	128	101	0.79	18602	0.013	0.023
T cell receptor signaling pathway (hsa04660)	103	85	0.83	14268	0.014	0.025
Cytosolic DNA-sensing pathway (hsa04623)	63	40	0.63	4993	0.015	0.025
B cell receptor signaling pathway (hsa04662)	82	61	0.74	11330	0.037	0.059
HIF-1 signaling pathway (hsa04066)	109	60	0.55	11748	0.043	0.065
Fc gamma R-mediated phagocytosis (hsa04666)	94	48	0.51	9608	0.055	0.080
Apoptosis (hsa04210)	136	78	0.57	12115	0.066	0.092
Prolactin signaling pathway (hsa04917)	70	51	0.73	7599	0.15	0.20
MAPK signaling pathway (hsa04010)	295	151	0.51	27384	0.16	0.20
Chemokine signaling pathway (hsa04062)	190	111	0.58	15084	0.16	0.20
Neurotrophin signaling pathway (hsa04722)	119	70	0.59	12388	0.18	0.22
VEGF signaling pathway (hsa04370)	59	39	0.66	7440	0.19	0.22
ErbB signaling pathway (hsa04012)	85	54	0.64	11201	0.28	0.31
FoxO signaling pathway (hsa04068)	132	67	0.51	10643	0.29	0.31
Adherens junction (hsa04520)	72	42	0.58	9633	0.29	0.31
Apoptosis - multiple species (hsa04215)	33	20	0.61	3722	0.31	0.32
Leukocyte transendothelial migration (hsa04670)	112	64	0.57	11444	0.35	0.35

FDR: False Discovery Rate

Supplementary Table S4. Pathway SLE polygenic risk scores, SLE patients positive for each pathway. The threshold for the pathway PRS was set at the 97.5th percentile in control individuals.

Pathway name (KEGG ID)	N SLE SNVs in PRS	N patients positive	Percent patients positive
Cytokine-cytokine receptor interaction (hsa04060)	297	390	40.7%
Osteoclast differentiation (hsa04380)	200	336	35.1%
MAPK signaling pathway (hsa04010)	263	333	34.8%
Jak-STAT signaling pathway (hsa04630)	214	276	28.8%
Chemokine signaling pathway (hsa04062)	158	256	26.7%
TNF signaling pathway (hsa04668)	144	247	25.8%
NF-kappa B signaling pathway (hsa04064)	157	246	25.7%
T cell receptor signaling pathway (hsa04660)	160	230	24.0%
Neurotrophin signaling pathway (hsa04722)	124	227	23.7%
HIF-1 signaling pathway (hsa04066)	113	223	23.3%
NOD-like receptor signaling pathway (hsa04621)	161	219	22.9%
Toll-like receptor signaling pathway (hsa04620)	138	212	22.1%
C-type lectin receptor signaling pathway (hsa04625)	136	209	21.8%
Complement and coagulation cascades (hsa04610)	73	198	20.7%
Th17 cell differentiation (hsa04659)	227	196	20.5%
Apoptosis (hsa04210)	118	190	19.8%
Leukocyte transendothelial migration (hsa04670)	106	190	19.8%
Hematopoietic cell lineage (hsa04640)	145	175	18.3%
Natural killer cell mediated cytotoxicity (hsa04650)	194	174	18.2%
Th1 and Th2 cell differentiation (hsa04658)	187	174	18.2%
B cell receptor signaling pathway (hsa04662)	128	172	18.0%
IL-17 signaling pathway (hsa04657)	102	171	17.8%
ErbB signaling pathway (hsa04012)	107	169	17.6%
RIG-I-like receptor signaling pathway (hsa04622)	91	158	16.5%
FoxO signaling pathway (hsa04068)	119	157	16.4%
Prolactin signaling pathway (hsa04917)	85	153	16.0%
Fc epsilon RI signaling pathway (hsa04664)	98	149	15.6%
Antigen processing and presentation (hsa04612)	127	149	15.6%
Fc gamma R-mediated phagocytosis (hsa04666)	107	143	14.9%
Viral protein interaction with cytokine and cytokine receptor (hsa04061)	96	143	14.9%
Adherens junction (hsa04520)	87	142	14.8%
VEGF signaling pathway (hsa04370)	81	133	13.9%
Cytosolic DNA-sensing pathway (hsa04623)	40	124	12.9%
Intestinal immune network for IgA production (hsa04672)	106	117	12.2%
Apoptosis - multiple species (hsa04215)	41	91	9.5%

Supplementary Table S5. Analysed gene-sets and their genes. Only sequenced genes are listed.

Gene set	Genes (RefSeq)	References
Interferon	*DDX58, IFI16, IFIH1, IFNA1, IFNA10, IFNA13, IFNA14, IFNA16, IFNA17, IFNA2, IFNA21, IFNA4, IFNA5, IFNA6, IFNA7, IFNA8, IFNAR1, IFNAR2, IFNB1, IFNE, IFNK, IFNW1, IRF5, IRF7, IRF9, JAK1, MAVS, PRKDC, STAT1, STAT2, STAT4, TYK2, ZBP1*	Rönnblom & Leonard, 2019 (PMID:31497305)
Interferonopathy	*ACP5, ADAR, C1QA, C1QB, C1QC, COPA, DDX58, IFIH1, ISG15, PSMB8, TREX1*	Rodero & Crow, 2016 (PMID:27821552); Rodero et al., 2017 (PMID:29259162); Davidson et al., 2018 (PMID:29846818)
SLE GWAS	*ABHD6, ATXN2, BACH2, BANK1, BLK, CD226, CD40, CD44, CD80, CDKN1B, CIITA, CLEC16A, CSK, CXCR5, ELF1, ETS1, EVI5, FAM167A, FAM98B, FCGR2A, GALC, GRAP2, GRB2, GTF2I, HCFC1, HIC2, HIP1, HLA-B, HLA-C, HLA-DQA1, HLA-DQA2, HLA-DRB1, ICAM1, IFIH1, IFNLR1, IKBKE, IKZF1, IKZF3, IL10, IL12A, IL12B, IL12RB2, IL21, IRAK1, IRF5, IRF7, IRF8, JAK2, LBH, LPP, LYN, LYST, MICB, MSH5, NCF2, NFKBIA, NOTCH2, NOTCH4, OLIG3, PHRF1, PLAT, PRDM1, PTPN22, PTPRC, PTTG1, PXK, RAD51B, RASGRP1, RASGRP3, RNF114, SH2B3, SLC15A4, SOCS1, SPRED2, STAT4, TCF7, TLR7, TNFAIP3, TNFSF4, TNIP1, TNPO3, TNXB, TRAFD1, TYK2, UBE2L3, WDFY4, YDJC, ZFP90*	Chen et al., 2017 (PMID:28509669); Langefeld et al., 2017 (PMID:28714469)
Complement	*C1QA, C1QB, C1QC, C1R, C1S, C2, C3, C3AR1, C4A, C4B, C4BPA, C5, C5AR1, C6, C7, C9, CD46, CD55, CD59, CFB, CFD, CFH, CFI, CR1, CR2, ITGAM, ITGAX, ITGB2, MASP1, MASP2, MBL2, SERPING1*	Complement subset of the KEGG Complement and coagulation cascades pathway (hsa04610)
Monogenic SLE	*ACP5, ADAR, C1QA, C1QB, C1QC, C1R, C1S, C2, C3, C4A, C4B, CYBB, DDX58, DNASE1L3, FAS, FASLG, IFIH1, ISG15, PRKCD, PSMB8, PTPN11, RAG1, RAG2, TREX1*	Tsokos et al., 2016 (PMID:27872476)

Supplementary Table S6. Top gene regions from aggregate testing (SKAT-O). Potentially novel loci are indicated in bold and only gene regions with FDR 0.05 or less are listed.

Gene region[a]	Chr	Start bp	End bp	N variants	Non-synonymous variants[b]	Conserved variants[c]	Top SNV[d]	FDR	P-value
TNXB	6	31908832	32177251	362	43	102	rs369580	1.7E-13	9.1E-17
PABPC4	1	39926385	40142621	132	10	45	rs879037	6.6E-06	4.3E-08
IFNK	9	27424212	27626596	231	7	58	rs895023	0.00081	1.2E-05
ATF2	2	175836878	176133034	166	4	64	rs79907460	0.0091	0.00016
SLC30A6	2	32290810	32549548	160	14	33	rs2365556	0.025	0.00048
SERPING1	11	57264927	57482426	167	14	42	rs12801093	0.04	0.0008
SRD5A2	2	31649556	31906140	48	0	3	rs28383069	0.04	0.00083
SPAST	2	32188580	32482806	190	12	31	rs2365556	0.042	0.0009
MEMO1	2	31992779	32336221	110	0	21	rs533970	0.046	0.0011

Only the top gene in the extended MHC on chr 6 is listed. Associations surviving Bonferroni correction for multiple testing (1832 gene regions) are above the line (P<2.7E-05). a) Gene regions included ±100kb to cover targeted regulatory regions; b) missense or nonsense variants; c) constrained variants defined as GERP RS score >2; d) Top associated SNV from the SLE case-control single variant logistic regression analysis. Bp: base pair, SNV: single nucleotide variant, FDR: False Discovery Rate.

Supplementary Table 7. Top gene regions from SLE case-control aggregate association testing including functional annotation (GenePy). Gene regions with P-values surviving permutation testing are listed.

Gene region	Chr	Start BP	End BP	SNVs in test	Annotation	P-value
MSH5	6	31706725	31731455	11	CADD	4.6E-15
CYP21A2	6	32005093	32010447	23	CADD	4.4E-12
TNXB	6	32007932	32078151	69	CADD	1.4E-11
HLA-DOB	6	32779540	32785825	74	CADD	7.2E-11
AGER	6	32147745	32153099	29	CADD	1.6E-10
HLA-DQB2	6	32722837	32732330	65	CADD	2.5E-09
MICA	6	31366561	31384090	196	CADD	2.3E-08
NOTCH4	6	32161620	32192844	120	CADD	2.7E-08
BTNL2	6	32361513	32375900	34	CADD	1.8E-07
BANK1	4	102710764	102996969	227	CADD	8.2E-07
HLA-C	6	31235526	31240913	169	CADD	9.5E-07
IRF5	7	128576976	128591089	44	CADD	2.2E-06
CFB	6	31912721	31920861	33	CADD	1.1E-05
HLA-DQA1	6	32604169	32613152	512	CADD	1.5E-05
MICB	6	31461054	31479901	13	REVEL	6.6E-24
TAP2	6	32788610	32807547	15	REVEL	1.1E-09
HLA-C	6	31235526	31240913	15	REVEL	5.8E-08
BTNL2	6	32361513	32375900	35	REVEL	3.5E-07
HLA-DQB1	6	32626241	32635466	18	REVEL	4.1E-06
CFB	6	31912721	31920861	14	REVEL	2.0E-05
MICA	6	31366561	31384090	26	REVEL	2.2E-05
BANK1	4	102710764	102996969	16	REVEL	3.4E-05
SLC15A4	12	129276739	129309541	5	REVEL	4.0E-05
HLA-B	6	31320649	31325989	12	REVEL	6.9E-05

CADD: weights including 63 annotations, such as conservation metrics, functional genomic data, transcript information and protein level scores;
REVEL: weights based on pathogenicity of rare missense variants.

Supplementary Table S8. Single variant SLE case-control association results Swedish cohort, independent loci with P<1x10-4.

CHR	BP	SNV	Locus	Minor/major allele	MAF case	MAF control	OR	FDR	P
6	31708463	rs3131381	*MSH5*	A/C	0.248	0.113	2.82	6.2E-23	6.7E-28
6	32612110	rs9273058	*HLA-DQA1*	C/T	0.339	0.504	0.49	2.9E-20	1.3E-23
6	31464798	rs2534675	*MICB*	T/C	0.449	0.292	2.02	6.0E-20	3.2E-23
7	128695983	rs13239597	*IRF5/TNPO3*	A/C	0.238	0.143	1.88	4.1E-11	1.1E-13
1	183542323	rs17849501	*NCF2*	T/C	0.108	0.053	2.33	3.3E-09	1.6E-11
2	191925424	rs3024859	*STAT4*	T/C	0.279	0.199	1.54	2.1E-06	1.4E-08
7	50308692	rs876037	*IKZF1*	A/T	0.242	0.312	0.66	3.4E-06	2.3E-08
16	11198932	rs35300161	*CLEC16A*	T/C	0.133	0.188	0.63	0.00013	1.2E-06
2	191919354	rs11676659	*STAT4*	G/A	0.033	0.063	0.46	0.00018	1.6E-06
6	106594719	rs2179175	*PRDM1*	T/C	0.278	0.222	1.44	0.00036	3.5E-06
9	27483959	rs895023	***MOB3B/IFNK***	G/A	0.026	0.056	0.45	0.00065	6.9E-06
12	129294244	rs11059926	*SLC15A4*	T/A	0.132	0.084	1.60	0.00088	9.5E-06
12	96374683	rs6538696	***HAL***	T/C	0.533	0.467	1.34	0.0013	1.4E-05
2	30961022	rs55799526	***CAPN13***	G/C	0.057	0.094	0.58	0.0018	2.1E-05
4	102340309	rs10433984	*BANK1*	A/T	0.301	0.250	1.37	0.0026	3.0E-05
5	150442171	rs3792790	*TNIP1*	C/A	0.459	0.529	0.75	0.0027	3.2E-05
6	138230040	rs200820567	*TNFAIP3*	A/T	0.061	0.035	1.89	0.0034	4.2E-05
10	64427649	rs7075349	*ZNF365/EGR2*	G/A	0.311	0.372	0.75	0.0041	5.0E-05
6	15168274	rs9396569	*JARID2*	A/G	0.143	0.107	1.48	0.0057	7.2E-05
19	10526854	rs34953890	*CDC37/TYK2*	A/C	0.170	0.220	0.71	0.0070	9.2E-05
10	128935768	rs12263483	*DOCK1/FAM196A*	G/A	0.101	0.142	0.67	0.0075	9.9E-05

Top SNP (or SNPs if independent) from each locus with at least one variant with P<1x10-4
Loci forwarded for replication are indcated in bold

Supplementary Table S9. Single variant SLE case-control association analysis in the Norwegian and Danish replication cohorts, and Sandinavian cohorts meta-analysis.

				**Norway**				**Denmark**				**Meta-analysis Scandinavia**			
**CHR**	**BP**	**SNV**	**Gene region**	**MAF SLE**	**MAF Ctrl**	**Odds ratio**	**P-value**	**MAF SLE**	**MAF Ctrl**	**Odds ratio**	**P-value**	**Odds ratio (R)**	**P-value (R)**	**Q**	**I^2**
2	30957326	rs2276568	*CAPN13*	0.081	0.063	1.32	0.19	0.080	0.061	1.33	0.067	1.03	0.93	0.00010	89
2	30961022	**rs55799526**	*CAPN13*	0.081	0.061	1.38	0.13	0.081	0.061	1.37	0.045	1.05	0.85	0.00010	90
2	30964632	rs72783018	*CAPN13*	0.087	0.064	1.37	0.12	0.075	0.063	1.22	0.21	1.06	0.74	0.012	77
2	30967587	rs72783020	*CAPN13*	0.087	0.063	1.41	0.093	0.078	0.063	1.27	0.13	1.08	0.70	0.0045	81
9	27482627	rs4879517	*MOB3B*	0.052	0.042	1.25	0.39	0.047	0.036	1.30	0.18	0.90	0.77	0	90
9	27483959	**rs895023**	*MOB3B*	0.052	0.051	1.01	0.96	0.047	0.037	1.26	0.23	0.83	0.58	0.00020	88
9	27489047	rs78313606	*MOB3B*	0.047	0.041	1.15	0.59	0.034	0.033	1.04	0.87	0.82	0.49	0.0034	82
9	27489418	rs76324761	*MOB3B*	0.047	0.041	1.15	0.59	0.034	0.033	1.04	0.87	0.82	0.49	0.0032	83
9	27498800	rs1031154	*IFNK/MOB3B*	0.054	0.042	1.30	0.30	0.046	0.038	1.21	0.32	0.91	0.78	0.00030	88
9	27502986	rs1977661	*IFNK/MOB3B*	0.087	0.092	0.93	0.70	0.088	0.092	0.95	0.70	0.96	0.63	0.96	0
12	96374750	rs2270318	*HAL*	0.39	0.36	1.16	0.18	0.42	0.41	1.05	0.54	1.15	0.0014	0.39	0

Top SLE associated SNVs from the Swedish SLE case-control cohort are indicated in bold. The the top SNV in *HAL* (rs6538696) failed genotyping.
Scandinavia meta-analysis included 1,794 SLE patients and 3,241 control individuals from Sweden, Norway and Denmark.
Random-effects meta-analysis (R) p-values and odds ratio estimates are shown.
MAF= Minor allele frequency, Q= P-value for Cochran's Q statistic, I^2= I2 heterogeneity index (0-100 scale).

Supplementary Table S10. Rare non-synonymous variants in genes for monogenic SLE and lupus-like disease.

								SLE (N=958)				Controls (N=1026)			
CHR	BP	SNV	REF	ALT	Gene name	Consequence	Amino acid change	Minor allele count	MAF	Count homozygous allele 1	Count hetero-zygotes	Minor allele count	MAF	Count homozygous allele 1	Count hetero-zygotes
1	22965341	rs772544683	G	A	*C1QA*	missense variant	p.Arg60Gln	0	0	0	0	1	0.00051	0	1
1	22973825	chr1:22973825	C	G	*C1QC*	missense variant	p.Pro96Arg	1	0.00056	0	1	0	0	0	0
1	22974053	rs560156356	C	T	*C1QC*	missense variant	p.Ala172Val	1	0.00056	0	1	0	0	0	0
1	154557500	chr1:154557500	G	A	*ADAR*	missense variant	p.Arg1198Trp	0	0	0	0	1	0.00049	0	1
1	154562881	rs145849344	C	T	*ADAR*	missense variant	p.Val802Ile	0	0	0	0	1	0.00049	0	1
1	154570903	rs17843865	T	C	*ADAR*	missense variant	p.Tyr630Cys	1	0.00052	0	1	0	0	0	0
1	154571009	rs771516546	C	T	*ADAR*	missense variant	p.Gly595Arg	0	0	0	0	1	0.00049	0	1
1	154573655	rs748585963	G	C	*ADAR*	missense variant	p.Pro531Arg	0	0	0	0	1	0.00049	0	1
1	154575074	chr1:154575074	T	G	*ADAR*	missense variant	p.His58Pro	0	0	0	0	1	0.00049	0	1
1	154600474	rs186420937	T	C	*ADAR*	start lost	p.Met1?	4	0.0021	0	4	3	0.0015	0	3
2	163124024	rs144455277	G	C	*IFIH1*	missense variant	p.Gln955Glu	2	0.0010	0	2	5	0.0024	0	5
2	163128887	rs376048533	C	T	*IFIH1*	missense variant	p.Arg822Gln	1	0.00052	0	1	0	0	0	0
2	163133396	rs72650663	G	A	*IFIH1*	missense variant	p.Thr702Ile	4	0.0021	0	4	1	0.00049	0	1
2	163134054	rs774347658	C	T	*IFIH1*	missense variant	p.Ala639Thr	1	0.00052	0	1	0	0	0	0
2	163134090	rs35744605	C	A	*IFIH1*	stop gained	p.Glu627*	8	0.0042	0	8	9	0.0044	0	9
2	163134176	rs200945986	C	T	*IFIH1*	missense variant	p.Arg598His	0	0	0	0	1	0.00049	0	1
2	163136564	rs142348767	A	C	*IFIH1*	missense variant	p.Leu528Arg	1	0.00052	0	1	0	0	0	0
2	163137999	rs772981550	T	C	*IFIH1*	missense variant	p.Asn455Asp	0	0	0	0	2	0.00097	0	2
2	163138050	rs139714761	A	G	*IFIH1*	missense variant	p.Ser438Pro	0	0	0	0	1	0.00049	0	1
2	163144659	rs775244788	C	T	*IFIH1*	missense variant	p.Val361Ile	1	0.00052	0	1	0	0	0	0
2	163144674	rs150317197	G	T	*IFIH1*	missense variant	p.Pro356Thr	1	0.00052	0	1	1	0.00049	0	1
2	163144694	rs72650664	T	C	*IFIH1*	missense variant	p.Lys349Arg	4	0.0021	0	4	0	0	0	0
2	163163228	chr2:163163228	C	T	*IFIH1*	missense variant	p.Val254Ile	1	0.00052	0	1	0	0	0	0
2	163167419	rs74162075	T	C	*IFIH1*	missense variant	p.Asn160Asp	2	0.0011	0	2	4	0.0020	0	4
2	163174487	rs760903088	G	T	*IFIH1*	missense variant	p.His111Asn	1	0.00052	0	1	0	0	0	0
2	163174615	chr2:163174615	T	C	*IFIH1*	missense variant	p.Lys68Arg	1	0.00053	0	1	0	0	0	0
3	48508028	rs749323787	G	A	*TREX1*	missense variant	p.Gly47Ser	1	0.00056	0	1	0	0	0	0
3	48508962	rs765487310	C	T	*TREX1*	missense variant	p.Thr358Ile	1	0.00053	0	1	0	0	0	0
3	53213596	rs376358631	G	A	*PRKCD*	missense variant	p.Arg40His	1	0.00055	0	1	0	0	0	0
3	53217499	chr3:53217499	C	T	*PRKCD*	missense variant	p.Pro230Leu	1	0.00055	0	1	0	0	0	0
3	53223210	rs146289210	C	T	*PRKCD*	missense variant	p.Thr564Met	1	0.00054	0	1	1	0.00050	0	1
3	58179012	rs766661698	C	T	*DNASE1L3*	missense variant	p.Ala287Thr	1	0.00052	0	1	1	0.00049	0	1
3	58191230	rs12491947	G	T	*DNASE1L3*	missense variant	p.Asn96Lys	9	0.0047	0	9	5	0.0024	0	5
3	58191274	rs74350392	C	G	*DNASE1L3*	missense variant	p.Gly82Arg	8	0.0042	0	8	13	0.0063	0	13
6	31901976	rs150827255	C	G	*C2*	missense variant	p.Ser250Cys	1	0.00052	0	1	0	0	0	0

6	31911055	rs142243595	G	A	*C2*	missense variant	p.Ala472Thr	0	0	0	0	1	0.00049	0	1
6	31912523	rs36221133	T	C	*C2*	missense variant	p.Val641Ala	10	0.0052	0	10	17	0.0083	0	17
6	31996241	rs764056735	G	A	*C4B*	missense variant	p.Met1054Ile	0	0	0	0	2	0.0010	1	0
6	31996773	rs781626894	A	G	*C4B*	missense variant	p.Ile1147Val	0	0	0	0	1	0.00050	0	1
6	32810551	chr6:32810551	C	A	*PSMB8*	missense variant	p.Arg102Leu	0	0	0	0	1	0.00049	0	1
6	32810794	rs17220206	T	A	*PSMB8*	missense variant	p.Thr74Ser	2	0.0010	0	2	1	0.00049	0	1
9	32457245	rs138425677	G	A	*DDX58*	missense variant	p.Pro885Ser	3	0.0016	0	3	0	0	0	0
9	32472997	rs139726764	G	A	*DDX58*	missense variant	p.Arg664Cys	1	0.00053	0	1	0	0	0	0
9	32480284	chr9:32480284	G	C	*DDX58*	missense variant	p.Asp569Glu	1	0.00052	0	1	0	0	0	0
9	32481339	rs61752945	C	T	*DDX58*	missense variant & splice region variant	p.Arg546Gln	11	0.0057	0	11	14	0.0068	0	14
9	32500832	rs72710678	C	T	*DDX58*	missense variant	p.Arg71His	22	0.011	0	22	13	0.0063	0	13
9	32526076	rs147964586	G	A	*DDX58*	missense variant	p.Ala30Val	1	0.00052	0	1	0	0	0	0
10	90771767	rs56006128	G	A	*FAS*	missense variant	p.Glu194Lys	4	0.0022	0	4	1	0.00050	0	1
11	36595579	rs76897604	A	G	*RAG1*	missense variant	p.Gln242Arg	14	0.0073	0	14	17	0.0084	0	17
11	36596695	chr11:36596695	G	A	*RAG1*	missense variant	p.Ser614Asn	0	0	0	0	1	0.00049	0	1
11	36597492	rs4151033	G	A	*RAG1*	missense variant	p.Glu880Lys	5	0.0026	0	5	0	0	0	0
11	36597513	rs4151034	G	A	*RAG1*	missense variant	p.Asp887Asn	10	0.0052	0	10	13	0.0064	1	11
11	36597870	rs139113046	A	G	*RAG1*	missense variant	p.Met1006Val	11	0.0057	0	11	15	0.0073	0	15
11	36614202	rs144812762	C	T	*RAG2*	missense variant	p.Arg506His	0	0	0	0	1	0.00049	0	1
11	36614521	rs140682926	C	G	*RAG2*	missense variant	p.Asp400His	1	0.00052	0	1	7	0.0034	0	7
11	36614574	chr11:36614574	T	G	*RAG2*	missense variant	p.Glu382Ala	0	0	0	0	1	0.00049	0	1
11	36614910	rs149241274	T	C	*RAG2*	missense variant	p.Glu270Gly	1	0.00052	0	1	0	0	0	0
11	65487283	chr11:65487283	G	C	*RNASEH2C*	missense variant	p.Ala234Gly	1	0.00053	0	1	0	0	0	0
11	65487341	chr11:65487341	T	C	*RNASEH2C*	missense variant	p.Met215Val	1	0.00053	0	1	0	0	0	0
11	65487503	rs182000627	C	T	*RNASEH2C*	missense variant	p.Gly161Arg	3	0.0017	0	3	0	0	0	0
11	65487511	rs753880827	C	G	*RNASEH2C*	missense variant	p.Ser158Thr	1	0.00056	0	1	0	0	0	0
12	7173211	rs781960849	A	G	*C1S*	missense variant	p.Ile270Val	1	0.00052	0	1	0	0	0	0
12	7173893	rs117907409	G	A	*C1S*	missense variant	p.Asp315Asn	3	0.0016	0	3	6	0.0029	0	6
12	7175028	rs20573	G	A	*C1S*	missense variant	p.Arg383His	2	0.0010	0	2	0	0	0	0
12	7175824	rs139493862	A	C	*C1S*	missense variant	p.Lys420Asn	0	0	0	0	1	0.00049	0	1
12	7177488	rs121909582	C	T	*C1S*	missense variant	p.Arg534Trp	1	0.00052	0	1	0	0	0	0
12	7241211	rs117402032	T	G	*C1R*	missense variant	p.Ile344Leu	1	0.00052	0	1	0	0	0	0
12	7242740	rs139531404	C	G	*C1R*	missense variant	p.Met111Ile	5	0.0026	0	5	10	0.0049	0	10
16	3705479	rs34907394	G	C	*DNASE1*	missense variant	p.Glu35Asp	6	0.0034	0	6	3	0.0016	0	3
16	3705900	rs755091005	C	G	*DNASE1*	missense variant	p.His66Gln	1	0.0006	0	1	0	0	0	0
16	3706754	chr16:3706754	G	C	*DNASE1*	missense variant & splice region variant	p.Glu146Gln	0	0	0	0	1	0.00051	0	1
16	3707066	rs150933932	C	T	*DNASE1*	missense variant	p.Ala168Val	5	0.0028	0	5	7	0.0036	0	7
16	3707191	rs74892550	G	A	*DNASE1*	missense variant	p.Val185Ile	0	0	0	0	11	0.0056	0	11
16	3707341	rs139254891	A	T	*DNASE1*	missense variant & splice region variant	p.Arg235Trp	1	0.00056	0	1	0	0	0	0

19	6678454	rs778590435	C	T	*C3*	missense variant	p.Arg1548Gln	1	0.00054	0	1	0	0	0	0
19	6697719	rs771305132	C	T	*C3*	missense variant	p.Glu843Lys	1	0.00053	0	1	0	0	0	0
19	6711182	rs532944249	G	A	*C3*	missense variant	p.Ser432Leu	0	0	0	0	1	0.00052	0	1
19	6719341	rs780548430	G	C	*C3*	missense variant	p.Gln50Glu	1	0.00057	0	1	0	0	0	0
19	11685942	rs147115345	G	A	*ACP5*	missense variant	p.Thr45Ile	1	0.00055	0	1	3	0.0015	0	3
19	11685989	rs147025508	G	A	*ACP5*	missense variant	p.Arg272Cys	10	0.0055	0	10	7	0.0036	0	7
19	11686037	rs146196342	C	G	*ACP5*	missense variant	p.Val256Leu	2	0.0011	0	2	1	0.00051	0	1
19	11687621	rs141651325	C	T	*ACP5*	missense variant	p.Arg100His	0	0	0	0	1	0.00051	0	1
19	11687630	rs777140546	C	T	*ACP5*	missense variant	p.Arg97His	0	0	0	0	1	0.00051	0	1
19	11688072	rs757630659	C	G	*ACP5*	missense variant	p.Gly21Arg	1	0.00057	0	1	0	0	0	0
19	12918104	rs754727728	T	C	*RNASEH2A*	missense variant	p.Val95Ala	0	0	0	0	1	0.00049	0	1
19	12920928	rs757486362	G	A	*RNASEH2A*	missense variant	p.Arg152Gln	0	0	0	0	2	0.00098	0	2
19	12921196	rs62619782	T	A	*RNASEH2A*	missense variant	p.Asp205Glu	12	0.0063	0	12	8	0.0039	0	8
19	12921216	rs377244188	A	T	*RNASEH2A*	missense variant & splice region variant	p.Asn212Ile	1	0.00052	0	1	2	0.00098	0	2
19	12923974	rs372667206	C	T	*RNASEH2A*	missense variant	p.Arg239Cys	1	0.00053	0	1	2	0.00097	0	2
X	37658266	chrX:37658266	G	A	*CYBB*	missense variant	p.Glu245Lys	0	0	0	0	1	0.00053	0	1
X	37663322	rs141756032	G	C	*CYBB*	missense variant	p.Gly364Arg	8	0.0045	0	8	9	0.0048	0	9

MAF: Minor Allele Frequency

Supplementary Table S11. SLE case-only non-synonymous variants. These are missense or nonsense SNVs observed in at least one SLE patient, but not in the control population or in external sets of control individuals of similar ancestry (SweGen and GnomAD: European non-Finnish controls; Ameur et al. 2017, Karczewski et al. 2019)

					SLE (n=958)				
CHR	BP	SNV	Minor allele	Major allele	Minor allele count	Minor allele frequency	Gene name	Consequence	Amino acid change
11	1266280	rs773068050	C	A	5	0.0028	*MUC5B*	missense variant	p.Thr2727Pro
1	186363103	chr1:186363103	A	C	4	0.0023	*C1orf27*	missense variant	p.Gln246Lys
1	151342270	rs772030489	T	G	2	0.0011	*SELENBP1*	missense variant	p.Pro36Thr
2	27455971	rs776014297	A	T	2	0.0010	*CAD*	missense variant	p.Met922Lys
2	179698928	chr2:179698928	A	G	2	0.0010	*CCDC141*	missense variant	p.Ser1522Phe
9	16431447	chr9:16431447	A	G	2	0.0011	*BNC2*	missense variant	p.His307Tyr
9	21166175	rs779242420	C	T	2	0.0010	*IFNA21*	missense variant	p.Tyr146Cys
10	75583821	chr10:75583821	T	G	2	0.0011	*CAMK2G*	missense variant	p.His370Asn
12	6458353	rs775543049	A	G	2	0.0010	*SCNN1A*	stop gained	p.Arg551*
12	48482728	rs750735162	C	T	2	0.0010	*SENP1*	missense variant	p.Thr79Ala
12	56350882	chr12:56350882	T	G	2	0.0010	*PMEL*	missense variant	p.Pro402His
12	129190793	chr12:129190793	G	C	2	0.0011	*TMEM132C*	missense variant	p.Pro1094Ala
14	23057866	chr14:23057866	T	A	2	0.0010	*DAD1*	missense variant	p.Ser66Arg
15	91030272	rs181919733	A	G	2	0.0010	*IQGAP1*	missense variant	p.Val1371Met
17	41143320	chr17:41143320	A	G	2	0.0011	*RUNDC1*	missense variant	p.Val477Ile
19	4891395	rs139019426	C	T	2	0.0010	*ARRDC5*	missense variant	p.Gln231Arg
19	18273781	rs777121279	A	G	2	0.0011	*PIK3R2*	missense variant	p.Gly372Ser
19	55240959	rs764066889	A	G	2	0.0010	*KIR3DL3*	missense variant & splice region variant	p.Gly219Asp
1	905700	rs759355675	G	C	1	0.00056	*PLEKHN1*	missense variant	p.Pro76Arg
1	1246066	chr1:1246066	T	C	1	0.00055	*PUSL1*	stop gained & splice region variant	p.Gln233*
1	3739745	rs148840465	T	C	1	0.00052	*CEP104*	missense variant	p.Gly855Glu
1	6529607	rs781318885	C	G	1	0.00055	*PLEKHG5*	missense variant	p.Pro723Ala
1	6535129	chr1:6535129	A	C	1	0.00055	*PLEKHG5*	missense variant	p.Ala173Ser
1	6589075	rs147263684	C	T	1	0.00052	*NOL9*	missense variant	p.Ile602Val
1	7798073	rs778503877	G	A	1	0.00052	*CAMTA1*	missense variant	p.Lys1238Arg
1	7812557	rs369220323	A	G	1	0.00052	*CAMTA1*	missense variant	p.Arg1641Gln
1	7837316	chr1:7837316	T	G	1	0.00052	*VAMP3*	missense variant	p.Ala57Ser
1	7879456	chr1:7879456	C	T	1	0.00052	*PER3*	missense variant	p.Ile545Thr
1	8075450	chr1:8075450	G	A	1	0.00052	*ERRFI1*	missense variant	p.Phe46Leu
1	8399719	chr1:8399719	T	G	1	0.00053	*SLC45A1*	missense variant	p.Leu681Phe
1	8424007	rs778046482	C	T	1	0.00056	*RERE*	missense variant	p.Lys134Glu
1	9786994	chr1:9786994	A	G	1	0.00052	*PIK3CD*	missense variant	p.Glu1033Lys
1	10195185	rs138056371	G	A	1	0.00052	*UBE4B*	missense variant	p.Asn722Ser
1	10386339	rs141942131	T	C	1	0.00052	*KIF1B*	missense variant	p.Thr949Met
1	10523664	chr1:10523664	A	G	1	0.00054	*DFFA*	missense variant	p.Ala152Val
1	11090845	chr1:11090845	C	G	1	0.00052	*MASP2*	stop gained	p.Tyr394*
1	11169772	chr1:11169772	G	C	1	0.00052	*MTOR*	missense variant	p.Val2461Leu
1	11255025	chr1:11255025	G	C	1	0.00052	*ANGPTL7*	missense variant	p.Thr329Ser
1	12336842	chr1:12336842	A	T	1	0.00052	*VPS13D*	missense variant	p.Val1066Asp
1	12337649	chr1:12337649	C	A	1	0.00052	*VPS13D*	missense variant	p.Glu1335Ala
1	12359263	chr1:12359263	A	G	1	0.00052	*VPS13D*	missense variant	p.Arg2013Lys
1	15855646	chr1:15855646	A	C	1	0.00052	*DNAJC16*	missense variant	p.Leu16Met
1	15892647	rs61738974	A	G	1	0.00052	*DNAJC16*	missense variant	p.Glu584Lys
1	16260959	chr1:16260959	C	A	1	0.00057	*SPEN*	missense variant	p.Thr2742Pro
1	17277609	rs757471392	T	C	1	0.00053	*CROCC*	missense variant	p.Arg1000Cys
1	19566385	chr1:19566385	A	T	1	0.00052	*EMC1*	missense variant	p.His294Leu
1	21952859	rs768044335	C	G	1	0.00052	*RAP1GAP*	missense variant	p.Gln38Glu
1	22062929	chr1:22062929	G	A	1	0.00052	*USP48*	missense variant	p.Phe109Ser
1	22078025	chr1:22078025	A	G	1	0.00052	*USP48*	missense variant	p.Thr250Ile
1	22329533	chr1:22329533	A	C	1	0.00054	*CELA3A*	missense variant	p.Ser27Arg
1	22973825	chr1:22973825	G	C	1	0.00056	*C1QC*	missense variant	p.Pro96Arg
1	24409181	rs148441250	A	G	1	0.00052	*MYOM3*	missense variant	p.Thr666Met
1	24484263	chr1:24484263	T	C	1	0.00053	*IFNLR1*	missense variant	p.Arg307Gln
1	25166361	chr1:25166361	C	G	1	0.00052	*CLIC4*	missense variant	p.Arg142Ser
1	26784313	chr1:26784313	T	C	1	0.00052	*DHDDS*	missense variant	p.Leu192Phe
1	26870645	chr1:26870645	G	C	1	0.00052	*RPS6KA1*	missense variant	p.Pro47Arg
1	26900915	rs755547404	A	G	1	0.00054	*RPS6KA1*	missense variant	p.Cys110Tyr
1	27734810	rs200665850	T	C	1	0.00052	*WASF2*	missense variant	p.Arg457Gln
1	32098045	chr1:32098045	A	G	1	0.00055	*HCRTR1*	missense variant	p.Glu366Lys

1	40038204	chr1:40038204	T	C	1	0.00052	*PABPC4*	missense variant	p.Arg83His
1	40125028	chr1:40125028	T	C	1	0.00056	*NT5C1A*	missense variant	p.Arg291Gln
1	43719800	chr1:43719800	T	G	1	0.00052	*WDR65*	missense variant	p.Ala1231Ser
1	43886632	chr1:43886632	A	C	1	0.00052	*SZT2*	missense variant	p.Arg492Ser
1	43907384	chr1:43907384	G	C	1	0.00053	*SZT2*	missense variant	p.Ala2489Gly
1	52840492	rs767782494	A	G	1	0.00052	*ORC1*	missense variant	p.Thr794Met
1	52902547	chr1:52902547	G	C	1	0.00052	*ZCCHC11*	missense variant	p.Asp1348His
1	57378187	chr1:57378187	T	C	1	0.00057	*C8A*	stop gained	p.Arg498*
1	57537275	chr1:57537275	T	G	1	0.00052	*DAB1*	missense variant	p.Leu160Ile
1	59156063	rs768128851	A	G	1	0.00057	*MYSM1*	missense variant	p.Pro82Leu
1	61554274	chr1:61554274	G	C	1	0.00052	*NFIA*	missense variant	p.Leu206Val
1	61554329	chr1:61554329	G	T	1	0.00052	*NFIA*	missense variant	p.Leu224Trp
1	63870189	chr1:63870189	G	A	1	0.00052	*ALG6*	missense variant	p.His108Arg
1	63998360	rs759233815	A	G	1	0.00052	*EFCAB7*	missense variant	p.Arg140Gln
1	64104375	rs751121095	G	A	1	0.00052	*PGM1*	missense variant	p.Ile368Val
1	65255098	chr1:65255098	A	C	1	0.00052	*RAVER2*	missense variant	p.Pro336Thr
1	65273113	chr1:65273113	G	A	1	0.00052	*RAVER2*	missense variant	p.Lys546Glu
1	65871673	chr1:65871673	A	G	1	0.00056	*DNAJC6*	missense variant	p.Gly783Asp
1	85790511	rs189798371	G	A	1	0.00052	*DDAH1*	missense variant	p.Ile218Thr
1	92442693	chr1:92442693	G	A	1	0.00053	*BRDT*	missense variant	p.Lys238Glu
1	92469982	chr1:92469982	G	T	1	0.00053	*BRDT*	missense variant	p.Ile800Met
1	92979452	chr1:92979452	C	T	1	0.00053	*EVI5*	missense variant	p.Ile743Val
1	100756962	chr1:100756962	C	A	1	0.00053	*RTCA*	missense variant	p.Lys348Gln
1	100964750	rs754033815	C	A	1	0.00052	*CDC14A*	missense variant	p.Thr563Pro
1	109395240	chr1:109395240	C	T	1	0.00052	*AKNAD1*	missense variant	p.Asp16Gly
1	109439703	rs747080388	A	G	1	0.00052	*GPSM2*	missense variant	p.Ala92Thr
1	112999589	chr1:112999589	C	T	1	0.00052	*CTTNBP2NL*	missense variant	p.Ile492Thr
1	114267385	rs747112740	C	T	1	0.00052	*PHTF1*	missense variant	p.Asn207Asp
1	114483711	rs747566932	T	A	1	0.00052	*HIPK1*	missense variant	p.Ser236Cys
1	114942135	chr1:114942135	G	C	1	0.00052	*TRIM33*	missense variant	p.Asp1022His
1	115110848	rs762953689	C	T	1	0.00052	*BCAS2*	missense variant	p.Glu194Gly
1	115238109	chr1:115238109	T	G	1	0.00052	*AMPD1*	stop gained	p.Ser28*
1	115258744	rs121434596	T	C	1	0.00052	*NRAS*	missense variant	p.Gly13Asp
1	115261341	chr1:115261341	C	G	1	0.00052	*CSDE1*	missense variant	p.Pro794Ala
1	115267874	rs779445127	G	A	1	0.00052	*CSDE1*	missense variant	p.Val620Ala
1	117087219	rs754340222	C	G	1	0.00054	*CD58*	missense variant	p.Ile26Met
1	120057084	rs769334806	T	C	1	0.00052	*HSD3B1*	missense variant	p.Pro315Leu
1	120496238	chr1:120496238	C	G	1	0.00052	*NOTCH2*	missense variant	p.Pro765Ala
1	120496289	chr1:120496289	G	C	1	0.00052	*NOTCH2*	missense variant	p.Gly748Arg
1	120496295	chr1:120496295	G	C	1	0.00052	*NOTCH2*	missense variant	p.Asp746His
1	150675830	rs144616489	T	A	1	0.00052	*HORMAD1*	missense variant	p.Ser330Thr
1	150675853	chr1:150675853	C	T	1	0.00052	*HORMAD1*	missense variant	p.Asn322Ser
1	150967779	chr1:150967779	A	G	1	0.00052	*ANXA9*	missense variant	p.Cys340Tyr
1	151131346	rs779652424	A	G	1	0.00055	*TNFAIP8L2*	missense variant	p.Arg58His
1	151134560	rs184513568	T	C	1	0.00052	*LYSMD1*	missense variant	p.Arg66His
1	151139661	chr1:151139661	C	G	1	0.00052	*SCNM1*	missense variant	p.Gln92His
1	151141491	chr1:151141491	C	G	1	0.00052	*SCNM1*	missense variant	p.Arg208Pro
1	151260226	rs751192864	A	G	1	0.00057	*ZNF687*	missense variant	p.Gly487Ser
1	151378048	chr1:151378048	T	G	1	0.00052	*POGZ*	missense variant	p.Pro1155Thr
1	151378361	chr1:151378361	A	C	1	0.00052	*POGZ*	missense variant	p.Leu1050Phe
1	151400443	chr1:151400443	T	C	1	0.00052	*POGZ*	missense variant	p.Glu312Lys
1	151747257	chr1:151747257	T	C	1	0.00052	*TDRKH*	missense variant	p.Ser521Asn
1	152085433	rs750586260	C	T	1	0.00052	*TCHH*	missense variant	p.Tyr87Cys
1	152187890	chr1:152187890	A	C	1	0.00055	*HRNR*	missense variant	p.Gly2072Val
1	152188572	rs760657166	T	A	1	0.00053	*HRNR*	missense variant	p.Tyr1845Asn
1	152191367	chr1:152191367	T	G	1	0.00055	*HRNR*	missense variant	p.Ser913Tyr
1	152191526	rs186602563	G	C	1	0.00053	*HRNR*	missense variant	p.Ser860Thr
1	152191748	rs761620569	A	C	1	0.00054	*HRNR*	missense variant	p.Gly786Val
1	152277317	chr1:152277317	A	C	1	0.00053	*FLG*	stop gained	p.Glu3349*
1	152280757	chr1:152280757	G	C	1	0.00052	*FLG*	missense variant	p.Gly2202Ala
1	152280764	chr1:152280764	T	C	1	0.00052	*FLG*	missense variant	p.Gly2200Arg
1	152284828	rs771331527	A	G	1	0.00053	*FLG*	missense variant	p.Pro845Leu
1	152323225	chr1:152323225	C	T	1	0.00052	*FLG2*	missense variant	p.His2346Arg
1	154033441	chr1:154033441	C	T	1	0.00052	*NUP210L*	missense variant	p.Ile909Val
1	154305114	chr1:154305114	A	G	1	0.00052	*ATP8B2*	missense variant	p.Arg210His
1	154317926	rs752493222	T	C	1	0.00052	*ATP8B2*	stop gained	p.Arg900*
1	154403033	rs576589094	T	C	1	0.00053	*IL6R*	missense variant	p.Arg137Trp
1	155014081	rs150822628	A	G	1	0.00053	*DCST1*	missense variant	p.Arg247His
1	155161981	chr1:155161981	C	T	1	0.00052	*MUC1*	missense variant	p.Lys51Arg

1	155173022	chr1:155173022	A	G	1	0.00053	*THBS3*	stop gained	p.Arg250*
1	155233065	chr1:155233065	A	G	1	0.00053	*CLK2*	missense variant	p.Leu482Phe
1	155269913	rs754454017	A	T	1	0.00057	*PKLR*	missense variant	p.Ser87Cys
1	155289628	rs781599688	G	A	1	0.00052	*FDPS*	missense variant	p.Lys323Arg
1	156621272	chr1:156621272	T	C	1	0.00053	*BCAN*	missense variant	p.Pro363Leu
1	156639290	chr1:156639290	T	C	1	0.00057	*NES*	missense variant	p.Gly1564Arg
1	156706476	chr1:156706476	G	C	1	0.00052	*RRNAD1*	missense variant	p.Asn453Lys
1	156785853	chr1:156785853	A	G	1	0.00055	*SH2D2A*	missense variant	p.Thr23Ile
1	156917740	rs766160542	A	T	1	0.00054	*ARHGEF11*	missense variant	p.Glu721Val
1	158151457	rs770171229	G	C	1	0.00052	*CD1D*	missense variant	p.Arg92Gly
1	158298796	chr1:158298796	A	T	1	0.00052	*CD1B*	missense variant	p.Thr299Ser
1	159019289	rs747906601	G	A	1	0.00052	*IFI16*	missense variant	p.His522Arg
1	160064898	chr1:160064898	T	C	1	0.00056	*IGSF8*	missense variant	p.Arg68Lys
1	160109762	chr1:160109762	T	C	1	0.00053	*ATP1A2*	missense variant	p.Arg1008Trp
1	160249951	chr1:160249951	C	T	1	0.00052	*DCAF8*	missense variant	p.Lys80Arg
1	160607014	chr1:160607014	G	C	1	0.00052	*SLAMF1*	missense variant	p.Val128Leu
1	160806025	rs191721381	A	G	1	0.00052	*CD244*	missense variant	p.Pro290Leu
1	160811227	chr1:160811227	G	C	1	0.00052	*CD244*	missense variant	p.Arg148Thr
1	160811631	chr1:160811631	T	G	1	0.00052	*CD244*	missense variant	p.Pro41Gln
1	161007805	chr1:161007805	T	G	1	0.00054	*TSTD1*	missense variant	p.Ala72Asp
1	161007806	chr1:161007806	T	C	1	0.00054	*TSTD1*	missense variant	p.Ala72Thr
1	161042555	chr1:161042555	T	C	1	0.00054	*PVRL4*	missense variant	p.Gly477Ser
1	161042596	chr1:161042596	A	G	1	0.00054	*PVRL4*	missense variant	p.Pro463Leu
1	161089130	rs754186210	G	C	1	0.00052	*NIT1*	missense variant	p.Ser102Cys
1	161141739	rs768971975	T	C	1	0.00052	*B4GALT3*	missense variant	p.Arg350Gln
1	161199591	rs771557332	G	C	1	0.00052	*NR1I3*	missense variant	p.Ser357Thr
1	162473602	chr1:162473602	T	C	1	0.00052	*UHMK1*	missense variant	p.Ala271Val
1	167815473	rs145191438	T	C	1	0.00052	*ADCY10*	missense variant	p.Met822Ile
1	169492469	rs756397912	C	T	1	0.00052	*F5*	missense variant	p.Gln2010Arg
1	173839561	rs779871322	A	G	1	0.00052	*ZBTB37*	missense variant	p.Met66Ile
1	173916519	rs758524800	C	T	1	0.00052	*RC3H1*	missense variant	p.Ile909Val
1	173930952	chr1:173930952	G	C	1	0.00052	*RC3H1*	missense variant	p.Val705Leu
1	179347845	rs150327590	G	T	1	0.00052	*AXDND1*	missense variant	p.Leu150Val
1	182555619	chr1:182555619	C	A	1	0.00052	*RNASEL*	missense variant	p.Leu108Arg
1	182616021	chr1:182616021	A	G	1	0.00052	*RGS8*	missense variant	p.Thr149Met
1	183253860	chr1:183253860	C	G	1	0.00052	*NMNAT2*	missense variant	p.Arg172Gly
1	183495785	rs374677970	C	T	1	0.00052	*SMG7*	missense variant	p.Ser123Pro
1	183536080	rs762173491	T	C	1	0.00052	*NCF2*	missense variant	p.Arg300Gln
1	185964009	rs751427036	T	A	1	0.00052	*HMCN1*	missense variant	p.Ile1190Phe
1	186024782	chr1:186024782	G	A	1	0.00052	*HMCN1*	missense variant	p.Lys2374Glu
1	186037025	chr1:186037025	A	C	1	0.00052	*HMCN1*	missense variant	p.Leu2589Ile
1	186064381	chr1:186064381	T	C	1	0.00052	*HMCN1*	missense variant	p.Pro3434Leu
1	186097361	chr1:186097361	T	G	1	0.00052	*HMCN1*	missense variant	p.Cys4281Phe
1	186113673	rs781091193	G	A	1	0.00052	*HMCN1*	missense variant	p.Lys4702Glu
1	186120434	rs774497609	G	A	1	0.00052	*HMCN1*	missense variant	p.Asp4904Gly
1	186143771	rs533893133	G	A	1	0.00052	*HMCN1*	missense variant	p.Met5314Val
1	186147655	rs144069476	A	G	1	0.00052	*HMCN1*	missense variant	p.Gly5351Arg
1	186158805	chr1:186158805	T	C	1	0.00052	*HMCN1*	missense variant	p.Thr5568Ile
1	186329113	chr1:186329113	A	C	1	0.00052	*TPR*	missense variant	p.Val403Leu
1	196696064	chr1:196696064	C	T	1	0.00052	*CFH*	missense variant	p.Cys744Arg
1	196715020	chr1:196715020	G	T	1	0.00052	*CFH*	stop gained	p.Tyr1128*
1	198671570	chr1:198671570	T	C	1	0.00052	*PTPRC*	missense variant	p.Pro165Leu
1	198678842	chr1:198678842	G	A	1	0.00058	*PTPRC*	missense variant	p.Ile354Val
1	200801885	chr1:200801885	G	A	1	0.00053	*CAMSAP2*	missense variant	p.Gln291Arg
1	200946014	chr1:200946014	A	G	1	0.00057	*KIF21B*	missense variant	p.Pro1445Ser
1	201012602	chr1:201012602	T	G	1	0.00052	*CACNA1S*	missense variant	p.Pro1619Thr
1	201013544	rs762847052	C	A	1	0.00055	*CACNA1S*	missense variant	p.Ile1570Ser
1	201020206	chr1:201020206	A	G	1	0.00053	*CACNA1S*	missense variant	p.Pro1340Leu
1	201060830	rs763260505	A	G	1	0.00053	*CACNA1S*	missense variant	p.Ala211Val
1	201772802	rs777253346	A	G	1	0.00052	*NAV1*	missense variant	p.Ser1200Asn
1	203143649	chr1:203143649	T	G	1	0.00056	*MYBPH*	missense variant	p.Asp139Glu
1	203148927	rs765522974	T	A	1	0.00053	*CHI3L1*	missense variant	p.Trp325Arg
1	203192776	chr1:203192776	T	C	1	0.00054	*CHIT1*	missense variant	p.Met109Ile
1	203767455	chr1:203767455	C	G	1	0.00052	*ZBED6*	missense variant	p.Val269Leu
1	205589257	chr1:205589257	G	A	1	0.00052	*ELK4*	missense variant	p.Val306Ala
1	207040733	chr1:207040733	T	C	1	0.00052	*IL20*	missense variant	p.Ala128Val
1	207134442	chr1:207134442	T	G	1	0.00056	*FCAMR*	missense variant	p.Thr260Lys
1	207240960	chr1:207240960	T	G	1	0.00052	*PFKFB2*	missense variant	p.Arg250Leu
1	207318001	rs770445743	C	T	1	0.00052	*C4BPA*	missense variant	p.Ile578Thr

1	207498991	chr1:207498991	G	A	1	0.00052	*CD55*	missense variant	p.Glu168Gly
1	207504518	rs748840094	T	G	1	0.00052	*CD55*	stop gained	p.Gly244*
1	207642048	rs769415969	T	C	1	0.00052	*CR2*	missense variant	p.Pro208Ser
1	207782637	chr1:207782637	T	A	1	0.00052	*CR1*	missense variant & splice region variant	p.Ile1967Leu
1	207790072	chr1:207790072	A	C	1	0.00052	*CR1*	missense variant	p.Arg2272Ser
1	207791569	chr1:207791569	A	G	1	0.00052	*CR1*	missense variant	p.Ser2348Asn
1	209783218	chr1:209783218	T	G	1	0.00052	*CAMK1G*	missense variant	p.Leu257Phe
1	211545599	chr1:211545599	C	T	1	0.00052	*TRAF5*	missense variant	p.Val410Ala
1	223283834	rs778914774	C	T	1	0.00052	*TLR5*	missense variant	p.Asn847Ser
1	223285383	chr1:223285383	T	C	1	0.00052	*TLR5*	missense variant	p.Ala331Thr
1	223285788	chr1:223285788	C	T	1	0.00052	*TLR5*	missense variant	p.Lys196Glu
1	223988446	chr1:223988446	G	C	1	0.00052	*TP53BP2*	missense variant	p.Gly431Ala
1	235969755	chr1:235969755	A	T	1	0.00052	*LYST*	missense variant	p.His894Leu
1	235976285	chr1:235976285	C	T	1	0.00052	*LYST*	missense variant	p.Glu90Gly
1	236706938	chr1:236706938	G	A	1	0.00052	*LGALS8*	missense variant	p.Asn252Asp
1	236730038	rs369632116	T	G	1	0.00052	*HEATR1*	missense variant	p.Leu1406Met
1	247013491	chr1:247013491	A	T	1	0.00052	*AHCTF1*	missense variant	p.Arg1974Ser
1	247013587	chr1:247013587	A	T	1	0.00052	*AHCTF1*	missense variant	p.Arg1942Ser
1	247027372	rs759273373	G	A	1	0.00052	*AHCTF1*	missense variant	p.Ser1167Pro
1	247040602	chr1:247040602	A	C	1	0.00052	*AHCTF1*	missense variant & splice region variant	p.Cys923Phe
1	247588157	chr1:247588157	G	C	1	0.00052	*NLRP3*	missense variant	p.Ser471Cys
2	11355692	rs753011445	C	T	1	0.00052	*ROCK2*	missense variant	p.Glu514Gly
2	23919236	chr2:23919236	C	T	1	0.00053	*KLHL29*	missense variant	p.Val653Ala
2	24092612	chr2:24092612	A	G	1	0.00052	*ATAD2B*	missense variant	p.His333Tyr
2	24302399	chr2:24302399	T	C	1	0.00052	*TP53I3*	missense variant	p.Gly244Asp
2	24360783	chr2:24360783	G	T	1	0.00054	*FAM228B*	missense variant	p.Leu58Arg
2	24522977	rs781391253	T	C	1	0.00052	*ITSN2*	missense variant	p.Arg382His
2	24930558	chr2:24930558	C	A	1	0.00052	*NCOA1*	missense variant	p.Lys740Thr
2	24952371	rs142674833	A	G	1	0.00052	*NCOA1*	missense variant & splice region variant	p.Gly963Glu
2	25045402	chr2:25045402	C	T	1	0.00052	*ADCY3*	missense variant	p.Asn994Ser
2	25471073	chr2:25471073	C	T	1	0.00055	*DNMT3A*	missense variant	p.Asn230Asp
2	26204555	rs769044393	T	C	1	0.00056	*KIF3C*	missense variant	p.Val78Met
2	26607864	chr2:26607864	C	T	1	0.00052	*EPT1*	missense variant	p.Tyr257His
2	26671665	chr2:26671665	A	C	1	0.00054	*DRC1*	missense variant	p.Asp501Glu
2	27015410	chr2:27015410	G	A	1	0.00053	*CENPA*	missense variant	p.Asn84Ser
2	27259596	chr2:27259596	A	G	1	0.00052	*TMEM214*	missense variant	p.Val284Met
2	27327510	chr2:27327510	G	C	1	0.00055	*CGREF1*	missense variant	p.Val31Leu
2	27351889	chr2:27351889	A	G	1	0.00052	*ABHD1*	missense variant	p.Asp118Asn
2	27500791	chr2:27500791	T	G	1	0.00052	*DNAJC5G*	missense variant	p.Asp95Tyr
2	27601915	chr2:27601915	A	G	1	0.00053	*ZNF513*	missense variant	p.Ser73Phe
2	27684321	chr2:27684321	T	C	1	0.00052	*IFT172*	missense variant	p.Glu753Lys
2	27851368	rs188110444	A	C	1	0.00052	*CCDC121*	missense variant	p.Arg88Leu
2	27887385	chr2:27887385	T	C	1	0.00052	*SLC4A1AP*	stop gained	p.Arg256*
2	28081344	rs147662293	T	C	1	0.00052	*RBKS*	missense variant	p.Ala62Thr
2	28999758	rs760562458	A	G	1	0.00052	*PPP1CB*	missense variant	p.Ala32Thr
2	29063332	chr2:29063332	C	G	1	0.00052	*SPDYA*	missense variant	p.Glu283Gln
2	29222155	chr2:29222155	C	G	1	0.00056	*FAM179A*	missense variant	p.Gly83Ala
2	30682588	chr2:30682588	C	G	1	0.00052	*LCLAT1*	missense variant & splice region variant	p.Gly37Ala
2	30966265	chr2:30966265	C	T	1	0.00052	*CAPN13*	missense variant	p.Met477Val
2	31425957	chr2:31425957	A	C	1	0.00052	*CAPN14*	missense variant	p.Asp92Tyr
2	31483576	rs773880032	T	G	1	0.00053	*EHD3*	missense variant	p.Ala235Ser
2	32422820	rs755069292	A	G	1	0.00052	*SLC30A6*	missense variant	p.Arg237Gln
2	32530604	rs753860958	C	T	1	0.00052	*YIPF4*	missense variant	p.Val215Ala
2	32735666	chr2:32735666	T	G	1	0.00052	*BIRC6*	missense variant	p.Gln3437His
2	33335810	rs755344524	T	C	1	0.00052	*LTBP1*	missense variant	p.Pro342Leu
2	33498801	chr2:33498801	C	T	1	0.00052	*LTBP1*	missense variant	p.Ile900Thr
2	37310480	rs764689790	C	G	1	0.00052	*HEATR5B*	missense variant	p.Ile26Met
2	39553381	chr2:39553381	T	C	1	0.00052	*MAP4K3*	missense variant	p.Gly190Ser
2	43514109	chr2:43514109	T	G	1	0.00052	*THADA*	missense variant	p.Ala1701Asp
2	45807071	chr2:45807071	C	G	1	0.00052	*SRBD1*	missense variant	p.Leu339Val
2	46378210	chr2:46378210	A	G	1	0.00053	*PRKCE*	missense variant	p.Val588Met
2	46602856	chr2:46602856	C	G	1	0.00054	*EPAS1*	missense variant	p.Gly305Ala
2	61275738	chr2:61275738	A	T	1	0.00052	*PEX13*	missense variant	p.Ser349Thr
2	61473490	chr2:61473490	G	C	1	0.00053	*USP34*	missense variant	p.Val2173Leu
2	68365908	chr2:68365908	T	A	1	0.00052	*WDR92*	missense variant	p.Met200Lys

2	68384615	chr2:68384615	G	A	1	0.00052	*RP11-474G23.1*	missense variant	p.Leu325Pro
2	68384796	chr2:68384796	T	C	1	0.00053	*RP11-474G23.1*	missense variant	p.Gly265Ser
2	69590685	chr2:69590685	C	T	1	0.00052	*GFPT1*	missense variant	p.Lys114Arg
2	69709811	chr2:69709811	T	C	1	0.00053	*AAK1*	missense variant	p.Met833Ile
2	69769789	chr2:69769789	A	C	1	0.00052	*AAK1*	missense variant	p.Val134Leu
2	73198715	chr2:73198715	G	T	1	0.00054	*SFXN5*	missense variant	p.Lys242Thr
2	74717515	chr2:74717515	C	G	1	0.00054	*TTC31*	missense variant	p.Ser126Thr
2	87015654	chr2:87015654	C	G	1	0.00052	*CD8A*	missense variant & splice region variant	p.Arg260Gly
2	98382607	rs751344985	T	C	1	0.00052	*TMEM131*	missense variant	p.Asp1565Asn
2	98428991	chr2:98428991	T	C	1	0.00052	*TMEM131*	missense variant	p.Gly586Arg
2	98431769	rs777316829	A	T	1	0.00052	*TMEM131*	missense variant & splice region variant	p.His319Leu
2	100623724	rs140526847	C	T	1	0.00052	*AFF3*	missense variant	p.Ile150Val
2	102507680	chr2:102507680	T	C	1	0.00052	*MAP4K4*	missense variant	p.Thr1311Ile
2	102509737	rs146402687	A	T	1	0.00052	*FLJ20373*	stop gained	p.Leu172*
2	102954780	rs771730337	T	A	1	0.00053	*IL1RL1*	missense variant	p.Lys19Met
2	102955351	chr2:102955351	T	A	1	0.00052	*IL1RL1*	missense variant	p.Gln39Leu
2	109513434	chr2:109513434	T	A	1	0.00054	*EDAR*	missense variant	p.Ser458Thr
2	128322932	chr2:128322932	C	T	1	0.00056	*MYO7B*	missense variant	p.Leu86Pro
2	128339504	chr2:128339504	G	C	1	0.00057	*MYO7B*	missense variant	p.His373Gln
2	128707502	chr2:128707502	T	C	1	0.00052	*SAP130*	missense variant	p.Arg939Gln
2	128712754	rs376315873	A	G	1	0.00053	*SAP130*	missense variant	p.Pro769Leu
2	133539592	rs780737770	T	G	1	0.00052	*NCKAP5*	missense variant	p.Leu1598Met
2	133539733	chr2:133539733	C	T	1	0.00052	*NCKAP5*	missense variant	p.Arg1551Gly
2	135712103	chr2:135712103	T	A	1	0.00052	*CCNT2*	missense variant	p.Glu693Val
2	135756467	rs141899370	C	G	1	0.00053	*MAP3K19*	missense variant	p.Arg139Gly
2	136362420	chr2:136362420	T	G	1	0.00053	*R3HDM1*	missense variant	p.Arg2Met
2	152132350	rs766955341	A	T	1	0.00052	*NMI*	missense variant	p.Lys123Asn
2	152319950	chr2:152319950	G	A	1	0.00053	*RIF1*	missense variant	p.Met1306Val
2	152330569	chr2:152330569	C	A	1	0.00053	*RIF1*	missense variant	p.Glu2396Ala
2	160599702	chr2:160599702	G	C	1	0.00052	*MARCH7*	missense variant	p.Ser95Cys
2	160824050	chr2:160824050	A	C	1	0.00052	*PLA2R1*	missense variant & splice region variant	p.Lys968Asn
2	163046214	chr2:163046214	C	T	1	0.00053	*FAP*	missense variant	p.Ile501Val
2	163066471	chr2:163066471	G	C	1	0.00052	*FAP*	missense variant	p.Trp346Cys
2	163080142	chr2:163080142	C	T	1	0.00052	*FAP*	missense variant	p.Tyr130Cys
2	163163228	chr2:163163228	T	C	1	0.00052	*IFIH1*	missense variant	p.Val254Ile
2	163174615	chr2:163174615	C	T	1	0.00053	*IFIH1*	missense variant	p.Lys68Arg
2	163204145	rs751929215	G	A	1	0.00052	*GCA*	missense variant	p.Thr29Ala
2	169622848	rs773417516	T	C	1	0.00052	*CERS6*	missense variant	p.Pro340Leu
2	169681188	chr2:169681188	A	C	1	0.00052	*NOSTRIN*	missense variant	p.Ala53Glu
2	169948301	rs754460573	T	C	1	0.00052	*DHRS9*	missense variant & splice region variant	p.Arg252Trp
2	170030471	rs764887934	C	T	1	0.00052	*LRP2*	missense variant	p.Asn3658Asp
2	170093681	chr2:170093681	T	G	1	0.00052	*LRP2*	missense variant	p.Ser1541Arg
2	170101218	chr2:170101218	T	C	1	0.00052	*LRP2*	missense variant	p.Asp1139Asn
2	170163901	rs759417883	A	C	1	0.00052	*LRP2*	missense variant	p.Ser106Ile
2	173352342	chr2:173352342	G	T	1	0.00052	*ITGA6*	missense variant	p.Phe782Cys
2	173429386	rs766194416	C	T	1	0.00052	*PDK1*	missense variant	p.Ile209Thr
2	179411904	rs541040798	A	G	1	0.00052	*TTN*	missense variant	p.Arg31450Cys
2	179416689	chr2:179416689	T	G	1	0.00052	*TTN*	stop gained	p.Ser30313*
2	179417556	chr2:179417556	A	G	1	0.00052	*TTN*	missense variant	p.Pro30024Leu
2	179419339	chr2:179419339	T	C	1	0.00052	*TTN*	missense variant	p.Val29579Ile
2	179436520	rs776673912	T	C	1	0.00052	*TTN*	missense variant	p.Arg24780Gln
2	179439589	chr2:179439589	G	A	1	0.00052	*TTN*	missense variant	p.Val23757Ala
2	179439763	chr2:179439763	T	C	1	0.00052	*TTN*	missense variant	p.Ser23699Asn
2	179439827	chr2:179439827	T	G	1	0.00052	*TTN*	missense variant	p.Gln23678Lys
2	179440124	chr2:179440124	C	A	1	0.00052	*TTN*	missense variant	p.Trp23579Gly
2	179441911	chr2:179441911	A	T	1	0.00052	*TTN*	missense variant	p.Asn23051Tyr
2	179443428	chr2:179443428	A	G	1	0.00052	*TTN*	missense variant	p.Pro22747Ser
2	179452524	chr2:179452524	T	G	1	0.00052	*TTN*	missense variant	p.Pro21171His
2	179463983	rs766906652	G	A	1	0.00052	*TTN*	missense variant	p.Ile18846Thr
2	179477250	chr2:179477250	A	C	1	0.00052	*TTN*	missense variant	p.Ala16668Ser
2	179486355	rs768414586	G	C	1	0.00052	*TTN*	missense variant	p.Gly15066Arg
2	179495856	chr2:179495856	T	C	1	0.00052	*TTN*	missense variant	p.Arg14640His
2	179499131	chr2:179499131	T	A	1	0.00053	*TTN*	missense variant	p.Val14126Glu
2	179500824	rs727504774	T	C	1	0.00052	*TTN*	missense variant	p.Arg13825Gln
2	179518183	chr2:179518183	T	G	1	0.00052	*TTN*	missense variant	p.Pro12893His

2	179527104	rs779791341	T	C	1	0.00055	*TTN*	missense variant	p.Val12334Met
2	179542425	rs764585210	G	A	1	0.00052	*TTN*	missense variant	p.Val11405Ala
2	179579754	chr2:179579754	A	T	1	0.00052	*TTN*	missense variant	p.Asp8720Val
2	179595004	chr2:179595004	T	C	1	0.00052	*TTN*	missense variant	p.Met6041Ile
2	179605752	rs397517830	T	C	1	0.00052	*TTN*	missense variant	p.Glu4070Lys
2	179621261	chr2:179621261	C	A	1	0.00052	*TTN*	missense variant	p.Ser3648Ala
2	179640287	rs767322897	T	C	1	0.00052	*TTN*	missense variant	p.Val2102Met
2	179664272	chr2:179664272	C	T	1	0.00054	*TTN*	missense variant	p.Ile286Val
2	181922463	chr2:181922463	G	A	1	0.00052	*UBE2E3*	missense variant	p.Ile97Val
2	182359500	chr2:182359500	G	A	1	0.00052	*ITGA4*	missense variant	p.Ile434Val
2	183703335	rs758921569	T	C	1	0.00052	*FRZB*	missense variant	p.Arg200Gln
2	183859591	chr2:183859591	A	C	1	0.00052	*NCKAP1*	missense variant	p.Asp262Tyr
2	198571321	chr2:198571321	T	G	1	0.00052	*MARS2*	stop gained	p.Glu398*
2	198948896	rs771861039	T	C	1	0.00052	*PLCL1*	missense variant	p.Arg219Trp
2	198949716	chr2:198949716	A	G	1	0.00052	*PLCL1*	missense variant	p.Gly492Glu
2	198949836	rs777477678	G	A	1	0.00052	*PLCL1*	missense variant	p.Lys532Arg
2	202093772	rs151011448	A	G	1	0.00052	*CASP10*	missense variant	p.Arg511Gln
2	203152396	chr2:203152396	G	A	1	0.00052	*NOP58*	missense variant	p.Arg150Gly
2	203420137	rs755765731	A	C	1	0.00052	*BMPR2*	missense variant	p.Asn583Lys
2	204736181	chr2:204736181	T	C	1	0.00052	*CTLA4*	missense variant	p.Leu180Phe
2	216229603	chr2:216229603	T	C	1	0.00052	*FN1*	missense variant & splice region variant	p.Arg2417Gln
2	216256433	chr2:216256433	A	G	1	0.00053	*FN1*	missense variant	p.Arg1392Cys
2	216263988	chr2:216263988	T	C	1	0.00052	*FN1*	missense variant	p.Gly1114Ser
2	219000467	rs200906463	A	G	1	0.00053	*CXCR2*	missense variant	p.Ala315Thr
2	219855702	chr2:219855702	C	G	1	0.00052	*CRYBA2*	missense variant	p.Ser106Arg
2	228155568	chr2:228155568	A	C	1	0.00052	*COL4A3*	stop gained	p.Ser1059*
2	231042338	chr2:231042338	C	A	1	0.00052	*SP110*	missense variant	p.Phe502Leu
2	233839504	rs186848841	T	C	1	0.00052	*NGEF*	missense variant	p.Glu33Lys
2	233995240	chr2:233995240	A	G	1	0.00052	*INPP5D*	missense variant	p.Ala183Thr
2	234231596	chr2:234231596	G	C	1	0.00052	*SAG*	missense variant	p.Pro127Arg
2	234255546	chr2:234255546	A	C	1	0.00052	*SAG*	missense variant	p.Asp402Glu
2	237406115	chr2:237406115	T	C	1	0.00052	*IQCA1*	missense variant	p.Met9Ile
2	241556395	rs753593237	C	A	1	0.00052	*CAPN10*	missense variant & splice region variant	p.Thr134Pro
3	419502	chr3:419502	G	A	1	0.00052	*CHL1*	missense variant & splice region variant	p.Ile585Val
3	423910	chr3:423910	A	G	1	0.00052	*CHL1*	missense variant	p.Arg642Lys
3	447213	rs139892128	C	G	1	0.00052	*CHL1*	missense variant	p.Arg1165Pro
3	3095627	rs749951952	A	G	1	0.00052	*CNTN4*	missense variant	p.Ser983Asn
3	3192625	chr3:3192625	C	G	1	0.00052	*CRBN*	missense variant	p.Thr418Arg
3	3192720	chr3:3192720	T	C	1	0.00052	*CRBN*	stop gained	p.Trp386*
3	4110287	chr3:4110287	A	C	1	0.00052	*SUMF1*	missense variant	p.Arg386Leu
3	4355357	rs752256260	T	C	1	0.00052	*SETMAR*	missense variant	p.Ser311Leu
3	4558204	rs751806213	G	A	1	0.00052	*ITPR1*	missense variant	p.His10Arg
3	4774844	chr3:4774844	A	C	1	0.00052	*ITPR1*	missense variant	p.Leu1750Ile
3	5249805	chr3:5249805	T	A	1	0.00052	*EDEM1*	missense variant	p.Ile456Phe
3	9704036	chr3:9704036	G	C	1	0.00052	*MTMR14*	missense variant	p.Pro132Ala
3	9785920	rs754915176	G	A	1	0.00054	*BRPF1*	missense variant	p.Asn883Ser
3	9868858	chr3:9868858	C	T	1	0.00052	*ARPC4-TTLL3*	missense variant	p.Val412Ala
3	10005877	chr3:10005877	G	T	1	0.00052	*EMC3*	missense variant	p.Glu221Ala
3	10312631	rs749767833	A	G	1	0.00053	*TATDN2*	missense variant	p.Val589Met
3	10319976	chr3:10319976	T	G	1	0.00055	*TATDN2*	missense variant	p.Gly659Cys
3	12544850	rs373274480	T	C	1	0.00052	*TSEN2*	missense variant	p.Thr133Met
3	15115413	rs762060370	C	T	1	0.00052	*ZFYVE20*	missense variant	p.Asp744Gly
3	17053032	chr3:17053032	G	A	1	0.00052	*PLCL2*	missense variant	p.Ile606Val
3	19974813	rs757699669	A	G	1	0.00052	*EFHB*	missense variant	p.Ala233Val
3	20027113	chr3:20027113	C	T	1	0.00053	*PP2D1*	missense variant	p.Glu551Gly
3	33093405	rs76016860	C	T	1	0.00052	*GLB1*	missense variant	p.Asp343Gly
3	38134338	rs765139538	A	G	1	0.00052	*DLEC1*	missense variant	p.Asp575Asn
3	38539176	chr3:38539176	A	G	1	0.00052	*EXOG*	missense variant	p.Ala74Thr
3	38545171	chr3:38545171	G	A	1	0.00053	*EXOG*	missense variant	p.Asp142Gly
3	38591990	rs199473331	T	C	1	0.00056	*SCN5A*	missense variant	p.Arg1958Gln
3	38592647	rs200217157	T	C	1	0.00054	*SCN5A*	missense variant	p.Arg1739Gln
3	42235341	rs376442996	C	T	1	0.00052	*TRAK1*	missense variant	p.Val309Ala
3	46003729	rs746250940	G	A	1	0.00052	*FYCO1*	missense variant	p.Ile1142Thr
3	46010120	rs768271085	A	G	1	0.00052	*FYCO1*	stop gained	p.Arg236*
3	46307012	chr3:46307012	G	C	1	0.00052	*CCR3*	missense variant	p.Ile142Met
3	46620841	chr3:46620841	G	A	1	0.00053	*TDGF1*	missense variant	p.Met70Val

3	48501189	chr3:48501189	T	C	1	0.00052	*ATRIP*	missense variant	p.Ser310Phe
3	49321912	chr3:49321912	T	C	1	0.00052	*USP4*	missense variant	p.Glu793Lys
3	49335323	chr3:49335323	A	C	1	0.00052	*USP4*	missense variant	p.Met557Ile
3	49456725	rs781466698	A	G	1	0.00055	*AMT*	missense variant	p.Arg222Cys
3	49569281	chr3:49569281	T	C	1	0.00055	*DAG1*	missense variant	p.Thr446Ile
3	49740157	chr3:49740157	A	C	1	0.00053	*RNF123*	missense variant	p.Ala574Asp
3	49753009	chr3:49753009	T	G	1	0.00057	*RNF123*	missense variant & splice region variant	p.Lys1004Asn
3	49830669	rs148137199	T	C	1	0.00053	*CDHR4*	missense variant	p.Val567Met
3	49833401	rs768814405	A	C	1	0.00052	*CDHR4*	missense variant	p.Val229Phe
3	50005081	rs368376344	C	T	1	0.00052	*RBM6*	missense variant	p.Tyr75His
3	50340155	chr3:50340155	C	T	1	0.00056	*HYAL1*	missense variant	p.Gln78Arg
3	50357323	chr3:50357323	A	G	1	0.00056	*HYAL2*	missense variant	p.Arg200Trp
3	50387151	chr3:50387151	A	G	1	0.00053	*NPRL2*	missense variant	p.Thr95Ile
3	50390686	chr3:50390686	A	G	1	0.00053	*CYB561D2*	missense variant	p.Met60Ile
3	50421754	chr3:50421754	C	A	1	0.00052	*CACNA2D2*	missense variant	p.Ser175Arg
3	50684588	rs749474530	T	C	1	0.00054	*MAPKAPK3*	missense variant	p.Thr317Met
3	50685473	chr3:50685473	G	A	1	0.00053	*MAPKAPK3*	missense variant	p.Gln382Arg
3	52282183	rs375720271	A	G	1	0.00054	*PPM1M*	missense variant	p.Arg242Gln
3	52360860	rs201173405	G	A	1	0.00054	*DNAH1*	missense variant	p.Ile231Val
3	52721370	chr3:52721370	T	C	1	0.00052	*GNL3*	missense variant	p.Leu61Phe
3	52812385	rs771292673	T	G	1	0.00052	*ITIH1*	missense variant	p.Leu56Phe
3	52816262	chr3:52816262	A	G	1	0.00052	*ITIH1*	missense variant	p.Met303Ile
3	52833406	rs758669573	A	G	1	0.00052	*ITIH3*	missense variant	p.Val270Met
3	52860574	chr3:52860574	T	A	1	0.00055	*ITIH4*	missense variant	p.Trp205Arg
3	52876865	rs779114178	G	A	1	0.00052	*TMEM110-MUSTN1*	missense variant	p.Tyr244His
3	53217499	chr3:53217499	T	C	1	0.00055	*PRKCD*	missense variant	p.Pro230Leu
3	53796108	chr3:53796108	G	C	1	0.00053	*CACNA1D*	missense variant & splice region variant	p.Asp1310Glu
3	53883794	chr3:53883794	T	G	1	0.00052	*IL17RB*	missense variant	p.Met66Ile
3	56675649	rs370672814	A	G	1	0.00052	*FAM208A*	missense variant	p.Arg783Cys
3	57902736	rs752100585	T	C	1	0.00052	*SLMAP*	missense variant	p.Arg731Trp
3	58381436	rs755436386	G	C	1	0.00052	*PXK*	missense variant	p.Gln258Glu
3	58487273	chr3:58487273	A	G	1	0.00052	*KCTD6*	missense variant	p.Val210Met
3	62189525	rs749942817	A	G	1	0.00053	*PTPRG*	missense variant	p.Ala686Thr
3	62259422	rs765533974	C	A	1	0.00052	*PTPRG*	missense variant	p.Lys1123Thr
3	62268508	rs778947846	G	C	1	0.00052	*PTPRG*	missense variant	p.Ala1340Gly
3	69079051	rs149174901	T	G	1	0.00052	*TMF1*	missense variant	p.Leu840Ile
3	69112200	rs547884414	C	T	1	0.00052	*UBA3*	missense variant	p.Asn209Ser
3	69230392	rs367603851	A	G	1	0.00052	*FRMD4B*	missense variant	p.Pro837Ser
3	69243012	chr3:69243012	C	G	1	0.00052	*FRMD4B*	missense variant	p.Gln501Glu
3	69244178	chr3:69244178	G	C	1	0.00052	*FRMD4B*	missense variant	p.Glu495Gln
3	69246116	rs754526422	C	T	1	0.00052	*FRMD4B*	missense variant	p.Ile343Val
3	69249968	chr3:69249968	T	A	1	0.00052	*FRMD4B*	missense variant	p.Ser2Thr
3	89390209	chr3:89390209	G	A	1	0.00052	*EPHA3*	missense variant	p.Met320Val
3	93596001	chr3:93596001	C	T	1	0.00052	*PROS1*	missense variant	p.Tyr560Cys
3	93615503	chr3:93615503	C	G	1	0.00053	*PROS1*	missense variant	p.Asp294Glu
3	93619659	chr3:93619659	C	T	1	0.00052	*PROS1*	missense variant	p.Lys239Arg
3	93783386	chr3:93783386	C	G	1	0.00052	*NSUN3*	missense variant	p.Val40Leu
3	97605508	chr3:97605508	A	C	1	0.00052	*CRYBG3*	missense variant	p.Gln448Lys
3	100362472	rs116356748	T	C	1	0.00053	*GPR128*	missense variant	p.Thr314Met
3	100535598	rs745848822	A	G	1	0.00052	*ABI3BP*	missense variant	p.Arg1062Trp
3	100548490	chr3:100548490	T	C	1	0.00052	*ABI3BP*	missense variant	p.Arg833Gln
3	119133678	rs761071117	A	G	1	0.00054	*ARHGAP31*	missense variant	p.Glu968Lys
3	119134605	chr3:119134605	T	G	1	0.00052	*ARHGAP31*	missense variant	p.Ala1277Ser
3	121980448	rs762998933	G	A	1	0.00052	*CASR*	missense variant	p.Asn189Ser
3	123663804	chr3:123663804	G	A	1	0.00052	*CCDC14*	missense variant	p.Met460Thr
3	123699321	chr3:123699321	C	T	1	0.00052	*ROPN1*	missense variant	p.Gln3Arg
3	128979481	rs760414395	T	C	1	0.00052	*COPG1*	missense variant	p.Ser320Leu
3	133327754	chr3:133327754	A	G	1	0.00052	*TOPBP1*	missense variant	p.Ala1409Val
3	133335724	chr3:133335724	C	T	1	0.00055	*TOPBP1*	missense variant	p.Ile1269Val
3	133347439	chr3:133347439	T	C	1	0.00052	*TOPBP1*	missense variant	p.Ala887Thr
3	133376680	chr3:133376680	T	C	1	0.00052	*TOPBP1*	missense variant	p.Val109Ile
3	133377907	chr3:133377907	G	A	1	0.00052	*TOPBP1*	missense variant	p.Tyr58His
3	136574215	rs140162609	G	A	1	0.00052	*SLC35G2*	missense variant	p.Ile305Val
3	138116198	chr3:138116198	A	G	1	0.00055	*MRAS*	missense variant	p.Ala76Thr
3	138192431	rs773319385	G	A	1	0.00052	*ESYT3*	missense variant	p.Tyr764Cys
3	138338582	chr3:138338582	T	A	1	0.00052	*FAIM*	missense variant	p.Asp5Val
3	139280070	rs761470404	A	T	1	0.00052	*NMNAT3*	missense variant	p.Ile181Phe

3	147121756	chr3:147121756	T	C	1	0.00052	*ZIC4*	missense variant	p.Glu44Lys
3	147131247	chr3:147131247	T	C	1	0.00054	*ZIC1*	missense variant	p.Thr418Ile
3	148759980	chr3:148759980	C	T	1	0.00052	*HLTF*	missense variant	p.Asn724Asp
3	148778593	chr3:148778593	C	T	1	0.00052	*HLTF*	missense variant	p.Thr405Ala
3	148793686	chr3:148793686	C	T	1	0.00052	*HLTF*	missense variant	p.Lys126Arg
3	148880486	chr3:148880486	A	G	1	0.00052	*HPS3*	missense variant	p.Ala768Thr
3	148889981	chr3:148889981	G	A	1	0.00052	*HPS3*	missense variant	p.Tyr996Cys
3	157082210	rs767375080	G	C	1	0.00052	*VEPH1*	missense variant	p.Val407Leu
3	157155302	rs778015862	A	C	1	0.00056	*PTX3*	missense variant & splice region variant	p.Pro44His
3	160395198	rs199795781	C	G	1	0.00052	*ARL14*	missense variant	p.Asp22His
3	160395595	rs773095286	G	A	1	0.00052	*ARL14*	missense variant	p.Gln154Arg
3	172163007	chr3:172163007	C	A	1	0.00052	*GHSR*	missense variant	p.Ser349Ala
3	172224304	rs758781458	T	A	1	0.00052	*TNFSF10*	missense variant	p.Phe275Tyr
3	175165038	chr3:175165038	A	G	1	0.00052	*NAALADL2*	missense variant	p.Arg371Gln
3	178957851	rs3914675	T	C	1	0.00055	*KCNMB3*	missense variant & splice region variant	p.Arg152His
3	186272723	chr3:186272723	A	C	1	0.00052	*TBCCD1*	missense variant	p.Cys337Phe
3	186314858	chr3:186314858	A	G	1	0.00053	*DNAJB11*	missense variant	p.Asp156Asn
3	186362569	chr3:186362569	T	C	1	0.00052	*FETUB*	missense variant	p.Pro152Ser
3	186457198	chr3:186457198	A	G	1	0.00052	*KNG1*	missense variant	p.Gly374Arg
3	186459931	chr3:186459931	C	G	1	0.00052	*KNG1*	missense variant	p.Gln582His
3	187088646	chr3:187088646	A	G	1	0.00052	*RTP4*	missense variant	p.Glu76Lys
3	188202428	chr3:188202428	C	T	1	0.00052	*LPP*	missense variant	p.Leu81Pro
3	190366297	chr3:190366297	A	G	1	0.00052	*IL1RAP*	missense variant	p.Val506Met
3	190373951	chr3:190373951	G	T	1	0.00052	*IL1RAP*	missense variant	p.Leu540Trp
3	190374046	chr3:190374046	G	A	1	0.00052	*IL1RAP*	missense variant	p.Arg572Gly
3	196534761	chr3:196534761	A	G	1	0.00052	*PAK2*	missense variant	p.Asp229Asn
3	196612156	chr3:196612156	G	A	1	0.00052	*SENP5*	missense variant	p.Gln35Arg
4	15060075	chr4:15060075	T	G	1	0.00052	*CPEB2*	missense variant	p.Ser831Ile
4	15443821	rs752265309	G	C	1	0.00052	*C1QTNF7*	missense variant	p.Leu97Val
4	15556750	rs376403848	T	A	1	0.00052	*CC2D2A*	missense variant	p.Met848Leu
4	15688863	chr4:15688863	C	T	1	0.00053	*FAM200B*	missense variant	p.Ile88Thr
4	15688937	chr4:15688937	T	G	1	0.00054	*FAM200B*	stop gained	p.Glu113*
4	15688940	chr4:15688940	A	C	1	0.00054	*FAM200B*	missense variant	p.Leu114Ile
4	26483291	chr4:26483291	T	C	1	0.00053	*CCKAR*	missense variant	p.Ser419Asn
4	36230220	chr4:36230220	G	T	1	0.00052	*ARAP2*	missense variant	p.Lys297Gln
4	36286149	chr4:36286149	G	A	1	0.00053	*DTHD1*	missense variant	p.Ile190Val
4	36292114	chr4:36292114	C	T	1	0.00052	*DTHD1*	missense variant	p.Val251Ala
4	36292306	chr4:36292306	T	G	1	0.00052	*DTHD1*	missense variant	p.Gly315Val
4	36309888	chr4:36309888	A	C	1	0.00052	*DTHD1*	missense variant	p.Ser538Tyr
4	36310070	chr4:36310070	A	G	1	0.00052	*DTHD1*	missense variant	p.Gly599Arg
4	36318049	rs542659542	C	G	1	0.00052	*DTHD1*	missense variant	p.Val676Leu
4	37445759	rs780612415	A	G	1	0.00052	*KIAA1239*	missense variant	p.Gly717Arg
4	38799470	rs772351679	T	A	1	0.00052	*TLR1*	missense variant	p.Ile328Asn
4	40104649	rs772027051	G	A	1	0.00052	*N4BP2*	missense variant	p.Tyr395Cys
4	40154468	chr4:40154468	A	G	1	0.00052	*N4BP2*	missense variant	p.Gly1738Arg
4	54364834	chr4:54364834	T	G	1	0.00052	*LNX1*	missense variant	p.Leu318Ile
4	55141054	rs201503614	T	C	1	0.00052	*PDGFRA*	missense variant	p.Pro567Leu
4	55145007	chr4:55145007	G	T	1	0.00052	*PDGFRA*	missense variant	p.Ser726Arg
4	55973917	chr4:55973917	T	C	1	0.00052	*KDR*	missense variant	p.Ala467Thr
4	57777652	chr4:57777652	G	A	1	0.00052	*REST*	missense variant	p.Tyr283Cys
4	57797767	rs372214552	A	G	1	0.00052	*REST*	missense variant	p.Glu915Lys
4	57860919	chr4:57860919	T	A	1	0.00052	*POLR2B*	missense variant	p.Met155Leu
4	68379930	rs777788368	T	C	1	0.00052	*CENPC*	missense variant	p.Val436Met
4	68383899	chr4:68383899	G	T	1	0.00053	*CENPC*	missense variant	p.Thr269Pro
4	74306380	chr4:74306380	G	C	1	0.00052	*AFP*	stop gained	p.Ser111*
4	74351688	rs758736794	C	A	1	0.00052	*AFM*	missense variant	p.Tyr127Ser
4	74853246	rs781398672	C	T	1	0.00052	*PPBP*	missense variant	p.Gln91Arg
4	74964321	rs201836008	G	T	1	0.00052	*CXCL2*	missense variant	p.Lys102Thr
4	76878845	rs773963650	G	C	1	0.00052	*SDAD1*	missense variant	p.Ser532Thr
4	79443912	rs752144269	C	G	1	0.00052	*FRAS1*	missense variant	p.Gln3586His
4	79461724	chr4:79461724	T	A	1	0.00054	*FRAS1*	missense variant	p.Ile3829Phe
4	80940090	chr4:80940090	G	C	1	0.00052	*ANTXR2*	missense variant	p.Val303Leu
4	95185874	chr4:95185874	G	A	1	0.00052	*SMARCAD1*	missense variant	p.Lys433Arg
4	100460497	chr4:100460497	G	C	1	0.00052	*C4orf17*	missense variant	p.Thr269Ser
4	101109090	chr4:101109090	G	T	1	0.00052	*DDIT4L*	missense variant	p.His109Pro
4	103517325	chr4:103517325	T	C	1	0.00052	*NFKB1*	missense variant	p.Pro444Leu
4	103533671	chr4:103533671	T	G	1	0.00052	*NFKB1*	missense variant	p.Ala834Ser

4	109862577	chr4:109862577	G	A	1	0.00052	*COL25A1*	missense variant	p.Ile170Thr
4	110603878	chr4:110603878	T	C	1	0.00052	*CCDC109B*	stop gained	p.Gln198*
4	110763628	chr4:110763628	A	T	1	0.00052	*RRH*	missense variant	p.Ser242Thr
4	110902062	rs762841951	T	C	1	0.00052	*EGF*	missense variant	p.Arg768Cys
4	123301319	rs375116122	T	C	1	0.00052	*ADAD1*	missense variant	p.Thr32Met
4	123542049	chr4:123542049	A	G	1	0.00052	*IL21*	missense variant	p.Arg40Cys
4	154624499	rs751213268	T	A	1	0.00052	*TLR2*	missense variant	p.His147Leu
4	154624940	rs755459719	G	A	1	0.00054	*TLR2*	missense variant	p.Asn294Ser
4	158142850	chr4:158142850	T	A	1	0.00052	*GRIA2*	missense variant	p.Glu40Asp
4	177608376	chr4:177608376	A	C	1	0.00052	*VEGFC*	missense variant	p.Leu370Phe
4	185599443	chr4:185599443	T	G	1	0.00053	*PRIMPOL*	missense variant	p.Arg301Leu
4	187003717	chr4:187003717	C	T	1	0.00052	*TLR3*	missense variant	p.Ser293Pro
4	187004453	chr4:187004453	T	A	1	0.00052	*TLR3*	missense variant	p.Gln538Leu
4	187070358	chr4:187070358	A	G	1	0.00053	*FAM149A*	missense variant	p.Gly200Ser
4	187074872	rs140771382	A	G	1	0.00056	*FAM149A*	missense variant	p.Ala345Thr
5	640631	chr5:640631	G	T	1	0.00056	*CEP72*	missense variant	p.Leu484Arg
5	7897206	chr5:7897206	A	G	1	0.00052	*MTRR*	missense variant	p.Gly627Arg
5	7897207	chr5:7897207	T	G	1	0.00052	*MTRR*	missense variant	p.Gly627Val
5	33453434	chr5:33453434	G	A	1	0.00052	*TARS*	missense variant	p.Asn157Asp
5	34935865	chr5:34935865	G	A	1	0.00052	*DNAJC21*	missense variant	p.Tyr81Cys
5	35807015	rs555353539	C	A	1	0.00052	*SPEF2*	missense variant	p.Arg1739Ser
5	37333578	chr5:37333578	T	A	1	0.00052	*NUP155*	missense variant	p.Leu502His
5	37443473	chr5:37443473	G	T	1	0.00052	*WDR70*	missense variant & splice region variant	p.Cys229Gly
5	38511918	chr5:38511918	A	T	1	0.00052	*LIFR*	missense variant	p.Asp237Val
5	38919318	rs556983548	T	C	1	0.00054	*OSMR*	missense variant	p.Thr139Met
5	39134434	chr5:39134434	C	T	1	0.00052	*FYB*	missense variant	p.Thr575Ala
5	39202725	chr5:39202725	G	A	1	0.00052	*FYB*	missense variant	p.Val123Ala
5	39341377	rs533953561	T	C	1	0.00052	*C9*	missense variant	p.Arg116Gln
5	40765321	chr5:40765321	C	G	1	0.00054	*PRKAA1*	missense variant	p.Gln296Glu
5	41057219	chr5:41057219	G	A	1	0.00052	*MROH2B*	missense variant	p.Leu304Pro
5	41159192	rs76528010	T	C	1	0.00052	*C6*	missense variant	p.Met616Ile
5	41159339	rs80108105	G	C	1	0.00052	*C6*	missense variant	p.Gln567His
5	41186246	rs756731027	G	A	1	0.00052	*C6*	missense variant	p.Cys218Arg
5	42800968	rs770189876	A	G	1	0.00052	*SEPP1*	missense variant	p.Arg334Trp
5	52235441	rs756236797	G	A	1	0.00052	*ITGA1*	missense variant	p.Ile1034Val
5	54527256	chr5:54527256	G	T	1	0.00053	*CCNO*	missense variant	p.Met334Leu
5	56177920	chr5:56177920	T	C	1	0.00052	*MAP3K1*	stop gained	p.Gln965*
5	66391496	rs370889678	T	C	1	0.00052	*MAST4*	missense variant	p.Thr305Met
5	66398343	rs760008201	A	G	1	0.00052	*MAST4*	missense variant	p.Gly166Glu
5	68555753	chr5:68555753	A	G	1	0.00052	*CDK7*	missense variant	p.Val173Ile
5	75913572	chr5:75913572	C	A	1	0.00052	*F2RL2*	missense variant	p.Ile320Met
5	76128892	chr5:76128892	G	T	1	0.00052	*F2RL1*	missense variant	p.Phe154Val
5	77396783	chr5:77396783	T	C	1	0.00053	*AP3B1*	missense variant	p.Asp822Asn
5	77458708	chr5:77458708	A	G	1	0.00052	*AP3B1*	missense variant	p.Thr433Ile
5	77536756	chr5:77536756	G	A	1	0.00052	*AP3B1*	missense variant	p.Ile70Thr
5	82786220	rs201689008	T	C	1	0.00052	*VCAN*	missense variant	p.Ala125Val
5	82837383	rs771479305	C	T	1	0.00052	*VCAN*	missense variant	p.Val2854Ala
5	82841445	rs773830482	C	T	1	0.00052	*VCAN*	missense variant	p.Tyr3119His
5	82841454	rs770608370	A	G	1	0.00052	*VCAN*	missense variant	p.Asp3122Asn
5	96232165	rs757590989	T	C	1	0.00052	*ERAP2*	missense variant	p.Pro443Leu
5	96235881	chr5:96235881	A	G	1	0.00052	*ERAP2*	missense variant	p.Met520Ile
5	108207125	chr5:108207125	G	A	1	0.00052	*FER*	missense variant	p.Asn242Ser
5	108380408	chr5:108380408	T	A	1	0.00052	*FER*	missense variant	p.Thr581Ser
5	110428236	chr5:110428236	A	C	1	0.00052	*WDR36*	missense variant	p.Pro84Thr
5	114952121	chr5:114952121	C	T	1	0.00053	*TMED7-TICAM2*	missense variant	p.Ile154Val
5	131676324	chr5:131676324	T	C	1	0.00052	*SLC22A4*	missense variant	p.Thr504Ile
5	131977997	chr5:131977997	C	G	1	0.00052	*RAD50*	missense variant	p.Asp1294His
5	132052578	rs759314163	A	T	1	0.00052	*KIF3A*	missense variant	p.Met239Leu
5	137519998	chr5:137519998	G	C	1	0.00052	*KIF20A*	missense variant	p.Arg475Gly
5	138163340	chr5:138163340	T	G	1	0.00053	*CTNNA1*	missense variant	p.Arg332Leu
5	138210173	rs371492043	G	A	1	0.00052	*LRRTM2*	missense variant	p.Met26Thr
5	139930668	chr5:139930668	G	C	1	0.00052	*SRA1*	missense variant	p.Lys159Asn
5	140035567	chr5:140035567	G	C	1	0.00052	*IK*	missense variant	p.Pro264Arg
5	140038586	rs755395781	A	G	1	0.00052	*IK*	missense variant	p.Arg338Gln
5	140073232	chr5:140073232	C	T	1	0.00052	*HARS2*	missense variant	p.Tyr89His
5	140797677	chr5:140797677	A	T	1	0.00052	*PCDHGB7*	stop gained	p.Leu84*
5	140798911	chr5:140798911	A	C	1	0.00056	*PCDHGB7*	missense variant	p.Asp495Glu
5	140870891	rs762200164	C	T	1	0.00053	*PCDHGC5*	missense variant	p.Ile695Thr

5	142780394	chr5:142780394	C	T	1	0.00052	*NR3C1*	missense variant	p.Lys4Arg
5	149274824	chr5:149274824	T	A	1	0.00052	*PDE6A*	missense variant	p.Ser550Arg
5	149375800	chr5:149375800	T	C	1	0.00055	*TIGD6*	missense variant	p.Gly38Ser
5	149384453	rs766913056	G	T	1	0.00052	*HMGXB3*	missense variant	p.Val13Gly
5	149429906	chr5:149429906	G	A	1	0.00054	*HMGXB3*	missense variant	p.Tyr1098Cys
5	149431355	chr5:149431355	A	T	1	0.00052	*HMGXB3*	missense variant	p.Ile1160Asn
5	150406568	chr5:150406568	A	C	1	0.00052	*GPX3*	missense variant	p.Pro117Thr
5	156566152	chr5:156566152	C	T	1	0.00052	*MED7*	missense variant	p.Ile97Met
5	156589871	rs201052186	T	A	1	0.00052	*FAM71B*	missense variant	p.Ser469Thr
5	156671418	rs772726821	C	T	1	0.00052	*ITK*	missense variant	p.Leu460Pro
5	179201217	rs578226932	G	A	1	0.00055	*MAML1*	missense variant	p.Tyr797Cys
5	179663388	chr5:179663388	G	C	1	0.00052	*MAPK9*	missense variant	p.Arg424Pro
5	180664023	rs770353665	A	G	1	0.00052	*GNB2L1*	missense variant	p.Thr321Met
6	3012847	chr6:3012847	C	T	1	0.00052	*NQO2*	missense variant	p.Leu81Pro
6	3127508	chr6:3127508	G	A	1	0.00052	*BPHL*	missense variant	p.Lys82Glu
6	15523376	rs74907982	A	G	1	0.00052	*DTNBP1*	missense variant	p.Pro297Ser
6	20546621	chr6:20546621	A	G	1	0.00052	*CDKAL1*	missense variant	p.Glu14Lys
6	21231095	chr6:21231095	C	T	1	0.00053	*CDKAL1*	missense variant	p.Leu522Pro
6	21231134	chr6:21231134	T	C	1	0.00053	*CDKAL1*	missense variant	p.Ala535Val
6	24559241	rs773691612	G	C	1	0.00052	*KIAA0319*	missense variant & splice region variant	p.Gly912Arg
6	30514598	rs375873464	T	C	1	0.00053	*GNL1*	missense variant	p.Arg486His
6	30878374	rs773111203	G	C	1	0.00055	*GTF2H4*	missense variant & splice region variant	p.Thr126Arg
6	30883526	chr6:30883526	G	C	1	0.00052	*VARS2*	missense variant	p.Arg160Gly
6	30885568	rs527633192	A	G	1	0.00052	*VARS2*	missense variant	p.Val354Met
6	30893952	rs761841915	A	G	1	0.00054	*VARS2*	missense variant	p.Asp1083Asn
6	31084556	chr6:31084556	A	G	1	0.00053	*CDSN*	missense variant	p.Pro279Leu
6	31474073	chr6:31474073	A	C	1	0.00052	*MICB*	missense variant	p.Thr160Asn
6	31603991	chr6:31603991	T	A	1	0.00053	*PRRC2A*	missense variant	p.Gln1877Leu
6	31603999	rs769170351	C	T	1	0.00054	*PRRC2A*	missense variant	p.Tyr1880His
6	31611688	chr6:31611688	A	G	1	0.00053	*BAG6*	missense variant	p.Leu579Phe
6	31727879	chr6:31727879	A	G	1	0.00052	*MSH5*	missense variant	p.Met583Ile
6	32065606	chr6:32065606	T	C	1	0.00052	*TNXB*	missense variant	p.Gly124Arg
6	32261258	chr6:32261258	T	C	1	0.00052	*C6orf10*	missense variant	p.Val398Ile
6	32411152	chr6:32411152	G	C	1	0.00052	*HLA-DRA*	missense variant	p.Phe173Leu
6	32610413	chr6:32610413	A	G	1	0.00052	*HLA-DQA1*	missense variant	p.Glu214Lys
6	32813369	chr6:32813369	A	T	1	0.00052	*TAP1*	missense variant	p.Asp805Val
6	33170472	chr6:33170472	T	G	1	0.00054	*SLC39A7*	missense variant	p.Gly312Val
6	33259953	chr6:33259953	A	G	1	0.00053	*RGL2*	missense variant	p.Pro754Ser
6	33289632	rs201782741	A	G	1	0.00053	*DAXX*	missense variant	p.Pro24Leu
6	33373314	rs775737916	C	A	1	0.00055	*KIFC1*	missense variant	p.Gln481Pro
6	35391806	chr6:35391806	A	C	1	0.00057	*PPARD*	missense variant	p.Pro170Thr
6	39883943	rs772980797	G	A	1	0.00053	*MOCS1*	missense variant	p.Ile151Thr
6	47251698	chr6:47251698	G	A	1	0.00052	*TNFRSF21*	missense variant	p.Trp407Arg
6	47541896	rs770123305	A	G	1	0.00052	*CD2AP*	missense variant	p.Gly213Glu
6	52619853	chr6:52619853	T	G	1	0.00052	*GSTA2*	missense variant	p.Gln54Lys
6	75890736	rs758201181	C	T	1	0.00052	*COL12A1*	missense variant	p.Thr695Ala
6	76576729	rs748073974	G	A	1	0.00052	*MYO6*	missense variant	p.Ile617Met
6	76596608	chr6:76596608	C	A	1	0.00052	*MYO6*	missense variant	p.Lys852Thr
6	84368754	rs771692440	C	T	1	0.00052	*SNAP91*	missense variant	p.Ile170Met
6	91260258	rs755212534	T	C	1	0.00052	*MAP3K7*	missense variant	p.Gly293Glu
6	111912986	rs573038591	C	G	1	0.00052	*TRAF3IP2*	missense variant	p.Leu111Val
6	123653072	chr6:123653072	C	G	1	0.00052	*TRDN*	missense variant & splice region variant	p.Pro475Ala
6	123818364	chr6:123818364	G	A	1	0.00053	*TRDN*	missense variant	p.Ile276Thr
6	136687472	rs751440841	A	G	1	0.00052	*MAP7*	stop gained	p.Arg350*
6	137147510	chr6:137147510	T	A	1	0.00052	*PEX7*	missense variant	p.His81Leu
6	137476140	rs141572567	A	G	1	0.00052	*IL22RA2*	missense variant	p.Ala137Val
6	137524806	chr6:137524806	G	A	1	0.00052	*IFNGR1*	missense variant	p.Leu188Pro
6	138192497	chr6:138192497	T	C	1	0.00052	*TNFAIP3*	stop gained	p.Arg45*
6	151914315	chr6:151914315	G	T	1	0.00052	*CCDC170*	missense variant	p.Met456Arg
6	154763322	rs776457984	G	C	1	0.00053	*CNKSR3*	missense variant	p.Ala107Pro
6	158323039	chr6:158323039	A	T	1	0.00052	*SNX9*	missense variant	p.Ser194Arg
6	159457400	rs754875937	T	C	1	0.00053	*TAGAP*	missense variant	p.Arg552Gln
6	161135891	chr6:161135891	T	G	1	0.00052	*PLG*	stop gained	p.Glu205*
6	168312072	rs747756056	T	C	1	0.00052	*MLLT4*	missense variant	p.Ser646Phe
6	168349154	chr6:168349154	A	T	1	0.00052	*MLLT4*	missense variant	p.Ile1268Asn
6	168363211	rs770828342	C	G	1	0.00052	*MLLT4*	missense variant	p.Gln1647His

6	170844456	rs576330081	A	G	1	0.00052	*PSMB1*	missense variant	p.Pro193Leu
6	170888039	chr6:170888039	G	C	1	0.00052	*PDCD2*	missense variant	p.Gly261Ala
7	1784536	chr7:1784536	A	G	1	0.00058	*ELFN1*	missense variant	p.Gly102Ser
7	2269759	rs747588764	T	G	1	0.00053	*MAD1L1*	missense variant	p.Leu4Met
7	2771015	chr7:2771015	T	C	1	0.00053	*GNA12*	missense variant	p.Asp316Asn
7	2834725	chr7:2834725	C	T	1	0.00053	*GNA12*	missense variant	p.Tyr121Cys
7	6472582	chr7:6472582	G	A	1	0.00053	*DAGLB*	missense variant	p.Ile143Thr
7	6476036	chr7:6476036	T	C	1	0.00052	*DAGLB*	missense variant	p.Asp126Asn
7	17373722	rs755496764	G	A	1	0.00052	*AHR*	missense variant	p.Ile298Val
7	22200139	chr7:22200139	T	C	1	0.00052	*RAPGEF5*	missense variant	p.Arg355His
7	22233141	chr7:22233141	A	C	1	0.00052	*RAPGEF5*	missense variant	p.Asp47Tyr
7	22330856	rs776635634	A	G	1	0.00052	*RAPGEF5*	missense variant	p.His76Tyr
7	38468970	rs747027899	C	T	1	0.00053	*AMPH*	missense variant	p.Ile214Val
7	50547491	chr7:50547491	T	C	1	0.00052	*DDC*	missense variant	p.Asp339Asn
7	55240758	rs746757722	G	A	1	0.00057	*EGFR*	missense variant	p.Met668Val
7	73654389	rs781896174	A	G	1	0.00053	*RFC2*	missense variant	p.Thr191Ile
7	80088004	rs761633220	G	C	1	0.00052	*GNAT3*	missense variant	p.Asp350His
7	80378261	chr7:80378261	T	C	1	0.00052	*SEMA3C*	missense variant	p.Ala617Thr
7	87168613	chr7:87168613	G	A	1	0.00052	*ABCB1*	missense variant	p.Tyr790His
7	87173539	rs773899676	T	G	1	0.00052	*ABCB1*	missense variant	p.Thr706Asn
7	87196151	rs200823786	C	T	1	0.00052	*ABCB1*	missense variant	p.Ile160Met
7	91503429	rs761977328	C	G	1	0.00052	*MTERF*	missense variant	p.His227Asp
7	91670175	chr7:91670175	G	A	1	0.00052	*AKAP9*	missense variant	p.Gln1639Arg
7	91715717	chr7:91715717	A	G	1	0.00053	*AKAP9*	missense variant	p.Arg3071Lys
7	91718772	chr7:91718772	T	C	1	0.00052	*AKAP9*	missense variant	p.Ala3100Val
7	91794015	chr7:91794015	G	A	1	0.00056	*LRRD1*	missense variant	p.Tyr168His
7	92163724	rs182459371	A	G	1	0.00052	*RBM48*	missense variant	p.Val153Met
7	99655414	chr7:99655414	T	C	1	0.00052	*ZSCAN21*	missense variant	p.Pro165Ser
7	99669713	chr7:99669713	A	G	1	0.00052	*ZNF3*	stop gained	p.Arg132*
7	99703161	rs373162905	T	C	1	0.00054	*AP4M1*	missense variant & splice region variant	p.Arg317Trp
7	99717341	chr7:99717341	T	G	1	0.00052	*TAF6*	missense variant	p.Thr10Asn
7	99764673	chr7:99764673	A	G	1	0.00056	*GAL3ST4*	missense variant	p.Arg16Trp
7	99767965	rs778573008	T	C	1	0.00056	*GPC2*	missense variant	p.Ser543Asn
7	100416243	rs541670683	A	G	1	0.00056	*EPHB4*	missense variant	p.Arg441Trp
7	103113308	chr7:103113308	G	A	1	0.00052	*RELN*	missense variant	p.Leu3445Pro
7	103197606	rs774088356	T	C	1	0.00052	*RELN*	missense variant & splice region variant	p.Ser1872Asn
7	103252239	chr7:103252239	A	T	1	0.00052	*RELN*	missense variant	p.Asp905Val
7	107671415	chr7:107671415	A	G	1	0.00052	*LAMB4*	stop gained	p.Gln1610*
7	107706234	rs765832432	C	T	1	0.00052	*LAMB4*	missense variant	p.Ile937Val
7	107706917	rs755148173	C	T	1	0.00052	*LAMB4*	missense variant	p.Ser859Gly
7	115889084	rs142430563	T	C	1	0.00052	*TES*	missense variant	p.Arg42Cys
7	116146049	rs759051961	G	C	1	0.00052	*CAV2*	missense variant	p.Leu59Val
7	116397800	chr7:116397800	G	A	1	0.00052	*MET*	missense variant	p.Ile692Val
7	116829445	chr7:116829445	A	C	1	0.00052	*ST7*	missense variant & splice region variant	p.Asp383Glu
7	123301938	chr7:123301938	A	G	1	0.00052	*LMOD2*	missense variant	p.Glu100Lys
7	123302077	chr7:123302077	A	G	1	0.00052	*LMOD2*	missense variant	p.Gly146Glu
7	123332562	chr7:123332562	G	C	1	0.00054	*WASL*	missense variant	p.Asp396His
7	123594181	chr7:123594181	G	A	1	0.00052	*SPAM1*	missense variant	p.Glu186Gly
7	128483963	chr7:128483963	G	T	1	0.00053	*FLNC*	missense variant	p.Asn975Lys
7	135078895	chr7:135078895	C	T	1	0.00052	*CNOT4*	missense variant	p.Thr468Ala
7	135095310	rs184784256	G	A	1	0.00053	*CNOT4*	missense variant	p.Val259Ala
7	140378974	chr7:140378974	T	C	1	0.00052	*ADCK2*	missense variant	p.Ala367Val
7	142965270	chr7:142965270	A	C	1	0.00053	*GSTK1*	missense variant	p.His264Gln
7	142988749	rs759719769	G	A	1	0.00052	*CASP2*	missense variant	p.Asp64Gly
7	143053968	chr7:143053968	G	A	1	0.00052	*FAM131B*	missense variant	p.Phe253Ser
7	154739693	chr7:154739693	A	G	1	0.00052	*PAXIP1*	stop gained	p.Arg948*
7	154760232	chr7:154760232	A	G	1	0.00059	*PAXIP1*	missense variant	p.Thr560Met
8	3205621	chr8:3205621	G	T	1	0.00052	*CSMD1*	missense variant	p.Asn1124His
8	3265630	chr8:3265630	T	C	1	0.00052	*CSMD1*	missense variant	p.Arg622Gln
8	3443747	rs777685797	C	T	1	0.00052	*CSMD1*	missense variant	p.Asn379Ser
8	11679272	chr8:11679272	C	T	1	0.00052	*FDFT1*	missense variant	p.Phe132Ser
8	17794782	chr8:17794782	T	C	1	0.00052	*PCM1*	missense variant	p.Thr79Ile
8	17796371	rs781151340	A	T	1	0.00052	*PCM1*	missense variant	p.Asp155Glu
8	17830188	rs778689206	G	A	1	0.00053	*PCM1*	missense variant	p.Lys1312Arg
8	27321446	chr8:27321446	A	G	1	0.00056	*CHRNA2*	missense variant	p.His172Tyr

8	27361123	rs759198021	A	G	1	0.00052	*EPHX2*	stop gained & splice region variant	p.Trp63*
8	32621624	chr8:32621624	C	A	1	0.00052	*NRG1*	missense variant	p.Asn616His
8	38001870	rs551783234	G	A	1	0.00053	*STAR*	missense variant	p.Leu260Pro
8	38005831	chr8:38005831	T	C	1	0.00053	*STAR*	missense variant	p.Glu65Lys
8	38258525	chr8:38258525	A	G	1	0.00052	*LETM2*	missense variant	p.Arg313His
8	42037762	rs771818476	G	A	1	0.00052	*PLAT*	missense variant & splice region variant	p.Ile407Thr
8	42178255	chr8:42178255	A	G	1	0.00054	*IKBKB*	missense variant & splice region variant	p.Glu507Lys
8	42188891	chr8:42188891	A	G	1	0.00052	*IKBKB*	missense variant	p.Arg91His
8	42218813	chr8:42218813	A	G	1	0.00052	*POLB*	missense variant & splice region variant	p.Gly184Asp
8	48719878	chr8:48719878	C	A	1	0.00052	*PRKDC*	missense variant	p.Phe3188Leu
8	48798584	rs775866201	A	G	1	0.00053	*PRKDC*	missense variant	p.Thr1565Met
8	56699017	rs769149695	T	C	1	0.00052	*TGS1*	missense variant	p.Thr187Ile
8	71499372	chr8:71499372	C	G	1	0.00052	*TRAM1*	missense variant	p.Leu204Val
8	73849779	chr8:73849779	A	C	1	0.00054	*KCNB2*	missense variant	p.Pro730Gln
8	73850063	chr8:73850063	G	A	1	0.00052	*KCNB2*	missense variant	p.Lys825Glu
8	90775067	rs764903626	A	G	1	0.00052	*RIPK2*	missense variant	p.Asp62Asn
8	99217434	rs762122486	G	C	1	0.00053	*NIPAL2*	missense variant	p.Met232Ile
8	99224722	chr8:99224722	C	T	1	0.00054	*NIPAL2*	missense variant	p.Glu189Gly
8	124790298	chr8:124790298	G	A	1	0.00052	*FAM91A1*	missense variant	p.Tyr134Cys
8	124824771	chr8:124824771	G	A	1	0.00053	*FAM91A1*	missense variant	p.Ile782Val
8	131193082	chr8:131193082	A	C	1	0.00052	*ASAP1*	missense variant	p.Gly192Val
8	133823311	chr8:133823311	A	G	1	0.00052	*PHF20L1*	missense variant	p.Val290Ile
8	133848818	chr8:133848818	T	C	1	0.00052	*PHF20L1*	missense variant	p.Ser648Leu
8	133899098	rs767101433	T	C	1	0.00052	*TG*	missense variant	p.Thr494Ile
8	134072345	chr8:134072345	T	C	1	0.00053	*SLA*	missense variant & splice region variant	p.Gly61Arg
8	141810650	chr8:141810650	T	A	1	0.00052	*PTK2*	missense variant	p.Leu378Gln
8	143927994	chr8:143927994	C	T	1	0.00054	*GML*	missense variant	p.Met122Thr
9	5431907	chr9:5431907	A	G	1	0.00052	*PLGRKT*	missense variant	p.Ala24Val
9	15474214	rs766532453	T	A	1	0.00053	*PSIP1*	missense variant	p.Ser217Arg
9	20981639	chr9:20981639	T	C	1	0.00052	*FOCAD*	missense variant	p.Ser1531Phe
9	21077511	rs761313571	C	T	1	0.00052	*IFNB1*	missense variant	p.Lys120Glu
9	21481264	rs543760462	G	C	1	0.00052	*IFNE*	missense variant	p.Val144Leu
9	27289724	chr9:27289724	C	T	1	0.00052	*EQTN*	missense variant	p.Asn143Asp
9	27562401	chr9:27562401	C	T	1	0.00052	*C9orf72*	missense variant	p.His193Arg
9	32472997	rs139726764	A	G	1	0.00053	*DDX58*	missense variant	p.Arg664Cys
9	32480284	chr9:32480284	C	G	1	0.00052	*DDX58*	missense variant	p.Asp569Glu
9	32526076	rs147964586	A	G	1	0.00052	*DDX58*	missense variant	p.Ala30Val
9	32630108	chr9:32630108	A	G	1	0.00053	*TAF1L*	missense variant	p.His1824Tyr
9	33038714	chr9:33038714	T	C	1	0.00052	*DNAJA1*	missense variant	p.Pro336Leu
9	34724712	chr9:34724712	A	G	1	0.00052	*FAM205A*	missense variant	p.Pro842Leu
9	35680970	chr9:35680970	T	C	1	0.00052	*CA9*	missense variant	p.Thr443Ile
9	35680976	chr9:35680976	T	G	1	0.00052	*CA9*	missense variant	p.Gly445Val
9	35684258	chr9:35684258	C	T	1	0.00052	*TPM2*	missense variant	p.Ile253Val
9	35714056	chr9:35714056	G	A	1	0.00052	*TLN1*	missense variant	p.Leu1048Ser
9	35736056	chr9:35736056	G	A	1	0.00052	*CREB3*	missense variant	p.Asp208Gly
9	35739077	rs752951773	A	G	1	0.00052	*GBA2*	missense variant	p.Arg579Trp
9	35740046	rs78197987	T	G	1	0.00052	*GBA2*	missense variant	p.Ala459Glu
9	35752131	chr9:35752131	T	G	1	0.00052	*RGP1*	missense variant	p.Cys354Phe
9	37126730	rs778387788	G	A	1	0.00052	*ZCCHC7*	missense variant	p.Lys134Arg
9	72741182	chr9:72741182	A	T	1	0.00052	*MAMDC2*	missense variant	p.Tyr251Asn
9	72840697	chr9:72840697	C	T	1	0.00054	*MAMDC2*	missense variant	p.Ile648Thr
9	72892266	chr9:72892266	T	G	1	0.00054	*SMC5*	missense variant	p.Asp141Tyr
9	75775217	rs746085433	A	C	1	0.00052	*ANXA1*	missense variant	p.His103Gln
9	86616625	rs770552137	T	C	1	0.00052	*RMI1*	missense variant	p.Pro242Ser
9	86617677	chr9:86617677	T	G	1	0.00053	*RMI1*	missense variant	p.Met592Ile
9	99278031	chr9:99278031	C	T	1	0.00052	*CDC14B*	missense variant	p.Ile458Val
9	99413985	chr9:99413985	T	C	1	0.00052	*AAED1*	missense variant	p.Val91Ile
9	100203947	rs749923554	C	A	1	0.00053	*TDRD7*	missense variant	p.Leu215Phe
9	100672673	chr9:100672673	A	T	1	0.00052	*C9orf156*	missense variant	p.His212Leu
9	100692605	rs748415690	T	G	1	0.00053	*HEMGN*	missense variant	p.Gln358Lys
9	101822201	chr9:101822201	A	C	1	0.00052	*COL15A1*	missense variant	p.Pro1123Gln
9	101894898	rs776680716	T	C	1	0.00052	*TGFBR1*	missense variant	p.Arg155Cys
9	116760613	rs534863329	T	C	1	0.00052	*ZNF618*	stop gained	p.Gln138*
9	123667366	chr9:123667366	T	A	1	0.00054	*TRAF1*	missense variant	p.Ser395Thr

9	123937321	chr9:123937321	G	A	1	0.00052	*CNTRL*	missense variant	p.Lys2258Arg
9	130506928	chr9:130506928	C	A	1	0.00056	*SH2D3C*	missense variant	p.Val572Gly
9	131469663	rs754199667	A	G	1	0.00055	*PKN3*	missense variant	p.Val272Met
9	134501399	rs779664732	A	G	1	0.00052	*RAPGEF1*	missense variant	p.Pro539Ser
9	135201814	rs770527045	T	C	1	0.00052	*SETX*	missense variant	p.Ser1724Asn
9	135275521	chr9:135275521	A	G	1	0.00052	*TTF1*	missense variant	p.Leu498Phe
9	136227153	chr9:136227153	T	C	1	0.00052	*SURF2*	missense variant	p.Thr177Ile
9	136270439	chr9:136270439	T	G	1	0.00056	*C9orf96*	missense variant	p.Arg646Leu
9	139270848	chr9:139270848	G	C	1	0.00058	*SNAPC4*	missense variant	p.Arg1457Pro
9	139324834	rs140543689	T	C	1	0.00055	*INPP5E*	missense variant	p.Gly566Asp
9	139350537	chr9:139350537	T	C	1	0.00054	*SEC16A*	missense variant	p.Val1878Ile
9	139371466	chr9:139371466	A	G	1	0.00057	*SEC16A*	missense variant	p.Ser201Phe
10	6066275	chr10:6066275	C	T	1	0.00052	*IL2RA*	missense variant	p.Glu100Gly
10	7601802	chr10:7601802	C	T	1	0.00052	*ITIH5*	missense variant	p.His681Arg
10	7618845	chr10:7618845	C	T	1	0.00053	*ITIH5*	missense variant	p.Lys517Glu
10	7759659	rs369417932	A	G	1	0.00052	*ITIH2*	missense variant	p.Glu180Lys
10	7838115	chr10:7838115	A	G	1	0.00058	*ATP5C1*	missense variant	p.Asp30Asn
10	11543198	chr10:11543198	A	G	1	0.00052	*USP6NL*	stop gained	p.Arg119*
10	14862121	chr10:14862121	G	T	1	0.00052	*CDNF*	missense variant	p.Lys141Thr
10	14885423	chr10:14885423	A	G	1	0.00055	*HSPA14*	missense variant	p.Cys92Tyr
10	14909263	chr10:14909263	C	T	1	0.00052	*HSPA14*	missense variant	p.Ile392Thr
10	14976478	chr10:14976478	A	C	1	0.00052	*DCLRE1C*	missense variant	p.Trp193Cys
10	16932418	chr10:16932418	A	G	1	0.00052	*CUBN*	stop gained	p.Gln2903*
10	17024493	rs770647065	A	G	1	0.00052	*CUBN*	missense variant	p.Ser1562Phe
10	17126274	rs771444469	G	A	1	0.00052	*CUBN*	missense variant	p.Ile766Thr
10	17737161	chr10:17737161	T	G	1	0.00052	*STAM*	missense variant	p.Ala217Ser
10	17756642	chr10:17756642	C	A	1	0.00052	*STAM*	missense variant	p.Thr496Pro
10	26518653	chr10:26518653	G	A	1	0.00052	*GAD2*	missense variant	p.Lys263Glu
10	26559597	chr10:26559597	G	C	1	0.00052	*GAD2*	missense variant	p.Thr335Arg
10	27048038	chr10:27048038	C	G	1	0.00053	*ABI1*	missense variant	p.Ser345Cys
10	28233155	chr10:28233155	C	T	1	0.00052	*ARMC4*	missense variant	p.Lys580Arg
10	33190536	rs775441007	T	C	1	0.00053	*ITGB1*	missense variant	p.Val787Ile
10	33552685	chr10:33552685	T	A	1	0.00052	*NRP1*	missense variant	p.Ser183Thr
10	35333504	chr10:35333504	C	T	1	0.00053	*CUL2*	missense variant	p.Tyr254Cys
10	49452876	rs774906204	G	A	1	0.00052	*FRMPD2*	missense variant	p.Leu109Ser
10	50085162	rs759570261	T	A	1	0.00052	*WDFY4*	missense variant	p.His2362Leu
10	60121125	chr10:60121125	A	C	1	0.00052	*UBE2D1*	missense variant	p.Pro18Thr
10	64159393	rs781556841	T	A	1	0.00052	*ZNF365*	missense variant	p.Met357Leu
10	64159409	chr10:64159409	G	C	1	0.00052	*ZNF365*	missense variant	p.Ala362Gly
10	69700756	chr10:69700756	C	T	1	0.00054	*HERC4*	missense variant	p.Lys823Arg
10	69934296	rs111965755	T	C	1	0.00052	*MYPN*	missense variant	p.Pro816Leu
10	70960036	chr10:70960036	C	G	1	0.00052	*SUPV3L1*	missense variant & splice region variant	p.Leu433Phe
10	72357879	chr10:72357879	A	G	1	0.00054	*PRF1*	missense variant	p.Thr533Ile
10	73544777	rs757570269	A	G	1	0.00056	*CDH23*	missense variant	p.Ala1883Thr
10	73553011	chr10:73553011	G	A	1	0.00052	*CDH23*	missense variant	p.Glu2114Gly
10	73569716	chr10:73569716	A	C	1	0.00055	*CDH23*	missense variant	p.Asp2959Glu
10	73580074	rs752687627	C	T	1	0.00052	*PSAP*	missense variant	p.Lys312Glu
10	75276999	rs748602458	A	G	1	0.00052	*USP54*	missense variant	p.Ala1062Val
10	75279564	rs769615440	C	T	1	0.00053	*USP54*	missense variant	p.Gln890Arg
10	75289405	chr10:75289405	G	A	1	0.00052	*USP54*	missense variant	p.Leu698Pro
10	75674612	rs371319040	C	T	1	0.00052	*PLAU*	missense variant	p.Met303Thr
10	75860818	chr10:75860818	G	A	1	0.00052	*VCL*	missense variant	p.Gln662Arg
10	82040072	chr10:82040072	A	C	1	0.00056	*MAT1A*	missense variant & splice region variant	p.Gly136Cys
10	87407007	chr10:87407007	T	G	1	0.00052	*GRID1*	missense variant	p.Asn715Lys
10	90429671	chr10:90429671	G	A	1	0.00052	*LIPF*	missense variant	p.His177Arg
10	90668464	rs756949249	G	T	1	0.00052	*STAMBPL1*	missense variant	p.Phe85Cys
10	90984962	chr10:90984962	A	G	1	0.00052	*LIPA*	missense variant	p.Pro188Ser
10	91066763	rs752866846	A	T	1	0.00052	*IFIT2*	stop gained	p.Tyr350*
10	91098604	chr10:91098604	A	T	1	0.00052	*IFIT3*	missense variant	p.Asp64Glu
10	91143404	rs771089871	A	G	1	0.00052	*IFIT1B*	missense variant	p.Ala112Thr
10	91196039	chr10:91196039	A	C	1	0.00052	*SLC16A12*	missense variant	p.Ala356Ser
10	95380726	rs762426409	T	C	1	0.00052	*PDE6C*	stop gained	p.Arg238*
10	95418757	chr10:95418757	T	G	1	0.00052	*PDE6C*	missense variant	p.Glu712Asp
10	95452470	chr10:95452470	T	C	1	0.00052	*FRA10AC1*	missense variant	p.Asp105Asn
10	97096366	chr10:97096366	C	G	1	0.00054	*SORBS1*	missense variant	p.Ala1138Gly
10	97101125	chr10:97101125	A	G	1	0.00052	*SORBS1*	missense variant	p.Pro1123Ser
10	97983740	chr10:97983740	A	G	1	0.00052	*BLNK*	stop gained	p.Arg123*

10	98079071	rs762908031	T	C	1	0.00052	*DNTT*	missense variant	p.Pro144Leu
10	98087323	rs763298852	C	G	1	0.00052	*DNTT*	missense variant	p.Asp325His
10	99211979	chr10:99211979	G	C	1	0.00052	*ZDHHC16*	missense variant	p.Leu126Val
10	99223635	chr10:99223635	C	G	1	0.00052	*MMS19*	missense variant	p.Ala631Gly
10	99350116	rs773907690	A	C	1	0.00053	*C10orf62*	missense variant	p.His154Gln
10	100182224	rs747984964	A	G	1	0.00054	*HPS1*	missense variant	p.Arg549Cys
10	103868821	rs773634189	T	G	1	0.00052	*LDB1*	missense variant	p.Pro324Thr
10	103900485	chr10:103900485	A	T	1	0.00053	*PPRC1*	missense variant	p.Ser740Arg
10	104269039	chr10:104269039	G	A	1	0.00052	*SUFU*	missense variant	p.Tyr99Cys
10	104592290	chr10:104592290	C	G	1	0.00055	*CYP17A1*	missense variant	p.His373Asp
10	105762263	rs776323344	A	G	1	0.00052	*SLK*	missense variant	p.Asp443Asn
10	105763139	rs372263493	G	A	1	0.00053	*SLK*	missense variant	p.Ile735Val
10	105781469	chr10:105781469	G	A	1	0.00053	*SLK*	missense variant	p.Glu1180Gly
10	105807547	chr10:105807547	C	A	1	0.00052	*COL17A1*	missense variant	p.Met762Arg
10	105830213	chr10:105830213	A	G	1	0.00052	*COL17A1*	missense variant	p.Thr193Ile
10	105920820	rs759122938	C	T	1	0.00052	*WDR96*	missense variant	p.Asn1172Ser
10	105971871	chr10:105971871	A	G	1	0.00052	*WDR96*	missense variant	p.Thr210Met
10	106019354	chr10:106019354	G	A	1	0.00052	*GSTO1*	missense variant	p.Asn55Ser
10	115349477	chr10:115349477	T	C	1	0.00054	*NRAP*	missense variant	p.Arg1687Gln
10	128785866	chr10:128785866	C	G	1	0.00052	*DOCK1*	missense variant	p.Trp103Cys
10	129172375	chr10:129172375	C	T	1	0.00052	*DOCK1*	missense variant	p.Phe1170Ser
11	6415551	chr11:6415551	A	T	1	0.00053	*SMPD1*	missense variant	p.Leu537His
11	6423431	rs775422772	T	A	1	0.00056	*APBB1*	missense variant	p.Asp421Glu
11	6561164	chr11:6561164	A	G	1	0.00054	*DNHD1*	missense variant	p.Arg1160Gln
11	12231089	rs777287062	T	C	1	0.00052	*MICAL2*	missense variant	p.Ser212Leu
11	12241757	rs556662753	A	G	1	0.00052	*MICAL2*	missense variant	p.Asp320Asn
11	12244252	chr11:12244252	C	T	1	0.00052	*MICAL2*	missense variant	p.Tyr471His
11	12278446	rs199868277	T	C	1	0.00053	*MICAL2*	missense variant	p.Arg1024Trp
11	16774416	chr11:16774416	A	G	1	0.00052	*C11orf58*	missense variant	p.Arg98Gln
11	17474719	rs749771128	T	C	1	0.00052	*ABCC8*	missense variant	p.Ala375Thr
11	17590736	chr11:17590736	A	G	1	0.00052	*OTOG*	missense variant	p.Val572Met
11	18050716	rs772454328	A	T	1	0.00052	*TPH1*	missense variant	p.Leu221Phe
11	18108433	rs758017855	C	T	1	0.00052	*SAAL1*	missense variant	p.Glu341Gly
11	18320479	chr11:18320479	C	A	1	0.00052	*HPS5*	missense variant	p.Cys342Gly
11	18357478	chr11:18357478	G	T	1	0.00052	*GTF2H1*	missense variant	p.Leu111Arg
11	18456337	rs777146300	T	C	1	0.00052	*LDHC*	missense variant	p.Arg157Cys
11	18579789	chr11:18579789	T	A	1	0.00052	*UEVLD*	missense variant	p.Val234Glu
11	18591767	chr11:18591767	T	C	1	0.00052	*UEVLD*	stop gained	p.Trp117*
11	18636215	chr11:18636215	C	T	1	0.00052	*SPTY2D1*	missense variant	p.Thr536Ala
11	18636536	rs555192381	T	C	1	0.00057	*SPTY2D1*	missense variant	p.Val429Ile
11	20950016	rs757768975	A	G	1	0.00052	*NELL1*	missense variant	p.Val358Ile
11	21592458	rs754480107	T	C	1	0.00052	*NELL1*	missense variant	p.Thr738Ile
11	35229669	rs773638372	G	A	1	0.00052	*CD44*	missense variant	p.Ser478Gly
11	46783596	chr11:46783596	A	G	1	0.00052	*CKAP5*	missense variant	p.Thr1392Met
11	47296560	chr11:47296560	A	G	1	0.00055	*MADD*	missense variant	p.Arg170His
11	47304084	chr11:47304084	G	A	1	0.00052	*MADD*	missense variant	p.Lys541Arg
11	47307987	chr11:47307987	A	G	1	0.00052	*MADD*	missense variant	p.Ser852Asn
11	57427180	rs374658636	T	C	1	0.00052	*CLP1*	missense variant	p.Arg89Cys
11	57506695	chr11:57506695	G	A	1	0.00052	*TMX2*	missense variant	p.Asp236Gly
11	57513051	chr11:57513051	C	T	1	0.00052	*BTBD18*	missense variant	p.Arg232Gly
11	57518639	chr11:57518639	C	T	1	0.00052	*BTBD18*	missense variant	p.Lys8Glu
11	58377398	chr11:58377398	T	A	1	0.00052	*ZFP91*	missense variant	p.Arg156Trp
11	59344376	rs761932791	T	C	1	0.00052	*OSBP*	missense variant	p.Arg728His
11	59574239	rs748789573	C	T	1	0.00053	*MRPL16*	missense variant	p.Met113Val
11	59596976	chr11:59596976	T	G	1	0.00052	*GIF*	missense variant	p.Ala412Asp
11	59626719	rs568414573	G	A	1	0.00052	*TCN1*	missense variant	p.Leu193Pro
11	59633874	rs750644060	T	C	1	0.00052	*TCN1*	missense variant	p.Glu24Lys
11	60665372	rs760498240	C	T	1	0.00052	*PRPF19*	missense variant	p.Thr455Ala
11	61578485	rs763381968	A	C	1	0.00052	*FADS1*	missense variant	p.Trp249Cys
11	63953439	chr11:63953439	A	T	1	0.00055	*STIP1*	missense variant	p.Leu48Gln
11	64532977	rs747776685	T	C	1	0.00059	*SF1*	missense variant	p.Gly534Ser
11	64851195	rs201897046	T	C	1	0.00054	*CDCA5*	missense variant & splice region variant	p.Gly16Glu
11	65422408	chr11:65422408	G	A	1	0.00055	*RELA*	missense variant	p.Met366Thr
11	65486195	chr11:65486195	A	G	1	0.00052	*KAT5*	missense variant	p.Gly467Arg
11	65487283	chr11:65487283	C	G	1	0.00053	*RNASEH2C*	missense variant	p.Ala234Gly
11	65487341	chr11:65487341	C	T	1	0.00053	*RNASEH2C*	missense variant	p.Met215Val
11	66241449	chr11:66241449	C	T	1	0.00056	*PELI3*	missense variant	p.Leu298Pro

11	66391439	rs754368323	A	G	1	0.00052	*RBM14*	stop gained & splice region variant	p.Trp135*
11	66391793	chr11:66391793	G	A	1	0.00054	*RBM14*	missense variant	p.Lys149Arg
11	66392401	chr11:66392401	T	G	1	0.00056	*RBM14*	missense variant	p.Ala352Ser
11	66407523	chr11:66407523	T	C	1	0.00052	*RBM4*	missense variant	p.Ala114Val
11	66453550	chr11:66453550	C	A	1	0.00055	*SPTBN2*	missense variant	p.Val2322Gly
11	67068445	chr11:67068445	A	G	1	0.00057	*ANKRD13D*	missense variant	p.Cys3Tyr
11	67164829	chr11:67164829	T	C	1	0.00057	*RAD9A*	missense variant	p.Thr351Ile
11	68575043	chr11:68575043	A	C	1	0.00055	*CPT1A*	missense variant	p.Trp115Cys
11	68575044	chr11:68575044	A	C	1	0.00055	*CPT1A*	missense variant	p.Trp115Leu
11	68660415	chr11:68660415	T	A	1	0.00052	*MRPL21*	missense variant	p.Ser166Thr
11	68665473	rs773363270	T	G	1	0.00052	*MRPL21*	missense variant	p.Pro52Thr
11	70009495	rs374970212	A	G	1	0.00052	*ANO1*	missense variant	p.Asp609Asn
11	71628685	chr11:71628685	A	C	1	0.00052	*RP11-849H4.2*	missense variant	p.Val140Phe
11	71701716	rs773012443	T	C	1	0.00052	*RNF121*	missense variant	p.Arg194Trp
11	71726456	rs752799135	C	T	1	0.00056	*NUMA1*	missense variant	p.Asp698Gly
11	76954889	chr11:76954889	C	A	1	0.00052	*GDPD4*	missense variant	p.Phe364Cys
11	77910694	chr11:77910694	C	T	1	0.00052	*USP35*	missense variant	p.Ile287Thr
11	77924720	chr11:77924720	A	C	1	0.00053	*USP35*	missense variant	p.Ala973Glu
11	77924795	chr11:77924795	A	G	1	0.00053	*USP35*	missense variant	p.Gly998Asp
11	77937852	chr11:77937852	C	T	1	0.00053	*GAB2*	missense variant	p.Asn289Ser
11	87020553	chr11:87020553	G	A	1	0.00052	*TMEM135*	missense variant	p.Ile259Val
11	89106654	rs528433051	C	T	1	0.00052	*NOX4*	missense variant	p.Thr382Ala
11	102201801	chr11:102201801	A	G	1	0.00052	*BIRC3*	missense variant	p.Ala385Thr
11	102564734	rs376387145	C	T	1	0.00052	*MMP27*	missense variant	p.Thr366Ala
11	102665941	rs186925812	A	G	1	0.00052	*MMP1*	missense variant	p.Thr288Ile
11	102824909	rs773554450	T	C	1	0.00053	*MMP13*	missense variant	p.Glu205Lys
11	108043929	rs192893789	C	A	1	0.00052	*NPAT*	missense variant	p.Asn594Lys
11	108121544	rs554805703	A	G	1	0.00052	*ATM*	missense variant	p.Arg451His
11	108158421	chr11:108158421	G	C	1	0.00054	*ATM*	missense variant	p.Thr1363Ser
11	108235896	rs786203721	A	C	1	0.00052	*ATM*	missense variant	p.Leu2980Ile
11	111896967	chr11:111896967	C	G	1	0.00052	*DLAT*	missense variant	p.Ala109Pro
11	111943820	chr11:111943820	A	G	1	0.00052	*PIH1D2*	missense variant	p.Pro27Ser
11	111953260	rs779558030	A	G	1	0.00053	*C11orf57*	missense variant	p.Arg149His
11	112072779	rs181898566	A	G	1	0.00052	*BCO2*	missense variant	p.Val354Ile
11	116729339	rs767005637	A	G	1	0.00052	*SIK3*	missense variant	p.Arg941Trp
11	117969714	rs149423953	A	C	1	0.00052	*TMPRSS4*	missense variant	p.Arg20Ser
11	118128020	chr11:118128020	C	T	1	0.00052	*MPZL2*	missense variant	p.Glu198Gly
11	118521223	rs781909091	T	G	1	0.00052	*PHLDB1*	missense variant	p.Arg1282Leu
11	118850225	rs369297325	G	C	1	0.00054	*FOXR1*	missense variant	p.Ala153Gly
11	119029055	chr11:119029055	A	G	1	0.00052	*ABCG4*	missense variant	p.Ala72Thr
11	119029587	chr11:119029587	G	A	1	0.00052	*ABCG4*	missense variant	p.Tyr462Cys
11	119045226	rs199476043	A	G	1	0.00053	*NLRX1*	missense variant	p.Arg305His
11	119050411	rs777542825	T	C	1	0.00053	*NLRX1*	stop gained	p.Arg561*
11	119050440	rs746329727	A	C	1	0.00053	*NLRX1*	missense variant	p.Ser570Arg
11	119061541	chr11:119061541	A	G	1	0.00056	*CCDC153*	stop gained	p.Gln120*
11	120198193	rs768909818	C	G	1	0.00052	*TMEM136*	missense variant	p.Gly37Arg
11	134022867	rs147905924	T	C	1	0.00052	*NCAPD3*	missense variant	p.Arg1490Gln
11	134074047	chr11:134074047	C	T	1	0.00052	*NCAPD3*	missense variant	p.Tyr370Cys
11	134119074	chr11:134119074	C	G	1	0.00052	*THYN1*	missense variant	p.Pro156Arg
12	6132847	rs756064548	T	A	1	0.00054	*VWF*	missense variant	p.Val1110Glu
12	6153527	rs61748477	A	G	1	0.00054	*VWF*	missense variant	p.Thr791Met
12	6637443	chr12:6637443	G	C	1	0.00054	*NCAPD2*	missense variant	p.Ala1083Gly
12	6923965	chr12:6923965	G	C	1	0.00053	*CD4*	missense variant	p.Ser138Arg
12	6924149	rs140406047	A	G	1	0.00053	*CD4*	missense variant	p.Val200Met
12	6940426	chr12:6940426	T	C	1	0.00053	*LEPREL2*	missense variant	p.Ser344Phe
12	6970139	chr12:6970139	G	A	1	0.00052	*USP5*	missense variant	p.Asn456Ser
12	7077710	rs782451030	T	G	1	0.00052	*PHB2*	stop gained	p.Cys108*
12	7173211	rs781960849	G	A	1	0.00052	*C1S*	missense variant	p.Ile270Val
12	7310658	rs79149590	A	C	1	0.00055	*CLSTN3*	missense variant	p.Thr963Asn
12	7894070	chr12:7894070	C	T	1	0.00052	*CLEC4C*	missense variant	p.Tyr61Cys
12	8196590	chr12:8196590	C	G	1	0.00052	*FOXJ2*	missense variant	p.Arg174Pro
12	8211697	rs370515640	T	C	1	0.00052	*C3AR1*	missense variant	p.Arg362Gln
12	8759510	rs759799595	T	C	1	0.00052	*AICDA*	missense variant	p.Arg36His
12	9086467	rs773756154	T	C	1	0.00052	*PHC1*	missense variant	p.Pro634Ser
12	9220787	rs770612196	T	C	1	0.00052	*A2M*	missense variant	p.Cys1467Tyr
12	14769643	chr12:14769643	T	C	1	0.00052	*GUCY2C*	missense variant	p.Ser965Asn
12	14849366	chr12:14849366	C	A	1	0.00052	*GUCY2C*	missense variant	p.Leu6Trp
12	15654593	chr12:15654593	T	A	1	0.00052	*PTPRO*	missense variant	p.Asn234Ile

12	25274804	chr12:25274804	T	G	1	0.00052	*CASC1*	missense variant	p.Phe374Leu
12	25380180	chr12:25380180	G	A	1	0.00052	*KRAS*	missense variant	p.Ile93Thr
12	31231344	chr12:31231344	C	A	1	0.00052	*DDX11*	missense variant	p.Gln6Pro
12	31236916	chr12:31236916	T	C	1	0.00052	*DDX11*	stop gained	p.Arg54*
12	32480528	rs201857634	T	G	1	0.00056	*BICD1*	missense variant	p.Gly380Val
12	32955405	chr12:32955405	T	C	1	0.00052	*PKP2*	missense variant	p.Ser744Asn
12	34179426	chr12:34179426	T	C	1	0.00052	*ALG10*	missense variant	p.Ser333Phe
12	39688290	rs373406994	A	C	1	0.00057	*KIF21A*	missense variant	p.Arg1655Leu
12	39760184	rs765082569	T	C	1	0.00052	*KIF21A*	missense variant	p.Ala291Thr
12	40694669	chr12:40694669	C	G	1	0.00052	*LRRK2*	missense variant	p.Gly198Ala
12	40702974	rs201013695	G	A	1	0.00052	*LRRK2*	missense variant	p.Tyr1419Cys
12	40713824	rs759685441	T	A	1	0.00054	*LRRK2*	missense variant	p.His1621Leu
12	40713825	rs765275134	A	C	1	0.00053	*LRRK2*	missense variant	p.His1621Gln
12	44176243	chr12:44176243	T	G	1	0.00052	*IRAK4*	missense variant	p.Ala359Ser
12	44196238	chr12:44196238	A	G	1	0.00054	*TWF1*	missense variant	p.Pro79Ser
12	48177940	chr12:48177940	G	A	1	0.00057	*HDAC7*	missense variant	p.Cys1004Arg
12	48457588	chr12:48457588	T	C	1	0.00052	*SENP1*	missense variant	p.Gly438Arg
12	48468218	chr12:48468218	T	G	1	0.00052	*SENP1*	missense variant	p.Pro277Thr
12	49298213	chr12:49298213	A	G	1	0.00052	*CCDC65*	missense variant	p.Ala32Thr
12	49315945	chr12:49315945	C	A	1	0.00052	*FKBP11*	missense variant	p.Leu143Arg
12	49390628	rs531994480	T	G	1	0.00052	*DDN*	missense variant	p.Ser677Arg
12	49421922	chr12:49421922	T	G	1	0.00055	*KMT2D*	missense variant & splice region variant	p.Asn4795Lys
12	49426595	chr12:49426595	A	G	1	0.00052	*KMT2D*	stop gained	p.Gln3965*
12	49426837	chr12:49426837	T	C	1	0.00056	*KMT2D*	missense variant	p.Ser3884Asn
12	49428064	chr12:49428064	C	T	1	0.00053	*KMT2D*	missense variant	p.Gln3509Arg
12	49432575	chr12:49432575	A	G	1	0.00055	*KMT2D*	missense variant	p.Ala2855Val
12	49447042	chr12:49447042	T	A	1	0.00052	*KMT2D*	missense variant	p.Phe301Tyr
12	51092172	chr12:51092172	C	T	1	0.00052	*DIP2B*	missense variant	p.Tyr704His
12	51102337	rs746963040	A	G	1	0.00052	*DIP2B*	missense variant	p.Val881Met
12	51203313	chr12:51203313	T	C	1	0.00052	*ATF1*	missense variant	p.Ala90Val
12	52284856	chr12:52284856	T	C	1	0.00054	*ANKRD33*	missense variant	p.Pro376Ser
12	53647053	rs750845106	C	T	1	0.00052	*MFSD5*	missense variant	p.Phe252Ser
12	53668773	chr12:53668773	A	G	1	0.00053	*ESPL1*	missense variant	p.Gly560Glu
12	53680248	chr12:53680248	A	T	1	0.00054	*ESPL1*	missense variant	p.Leu1243Gln
12	53701436	rs767509596	T	C	1	0.00055	*AAAS*	missense variant	p.Arg493His
12	54920358	rs779095429	A	G	1	0.00052	*NCKAP1L*	missense variant	p.Val735Ile
12	54926017	chr12:54926017	G	C	1	0.00052	*NCKAP1L*	missense variant	p.Pro949Ala
12	54966942	rs137936270	T	C	1	0.00052	*PDE1B*	missense variant	p.Ser249Leu
12	56296101	chr12:56296101	A	G	1	0.00056	*WIBG*	missense variant	p.Pro57Leu
12	56351432	chr12:56351432	G	C	1	0.00053	*PMEL*	missense variant	p.Val219Leu
12	56363280	chr12:56363280	A	G	1	0.00052	*CDK2*	missense variant	p.Ala170Thr
12	56397574	chr12:56397574	G	A	1	0.00052	*SUOX*	missense variant	p.Glu134Gly
12	56491576	chr12:56491576	C	A	1	0.00052	*ERBB3*	missense variant	p.Tyr823Ser
12	56527902	rs771895295	T	G	1	0.00052	*ESYT1*	missense variant	p.Lys504Asn
12	56743969	rs774874986	T	C	1	0.00052	*STAT2*	missense variant	p.Arg374Gln
12	57397455	rs377628250	A	G	1	0.00052	*ZBTB39*	missense variant	p.Thr416Ile
12	57605078	chr12:57605078	A	C	1	0.00056	*LRP1*	missense variant	p.Arg4346Ser
12	57843223	chr12:57843223	A	T	1	0.00052	*INHBC*	missense variant	p.Asn159Lys
12	57843425	chr12:57843425	A	G	1	0.00055	*INHBC*	missense variant	p.Val227Ile
12	57922958	chr12:57922958	T	C	1	0.00053	*MBD6*	missense variant	p.Pro985Ser
12	58020616	chr12:58020616	A	G	1	0.00053	*B4GALNT1*	missense variant	p.Arg505Cys
12	58180073	chr12:58180073	C	A	1	0.00052	*TSFM*	missense variant & splice region variant	p.Glu120Ala
12	66547210	rs767684716	C	T	1	0.00053	*TMBIM4*	missense variant	p.Tyr39Cys
12	68708729	chr12:68708729	T	C	1	0.00052	*MDM1*	missense variant & splice region variant	p.Gly500Arg
12	72771892	rs752459412	T	C	1	0.00055	*TRHDE*	missense variant	p.Pro391Ser
12	96360155	rs79312289	G	C	1	0.00052	*AMDHD1*	missense variant	p.Asn354Lys
12	99192750	chr12:99192750	T	C	1	0.00052	*ANKS1B*	missense variant	p.Gly1077Arg
12	103249110	rs199475652	C	A	1	0.00052	*PAH*	missense variant & splice region variant	p.His170Gln
12	104046434	chr12:104046434	C	T	1	0.00052	*STAB2*	missense variant	p.Met453Thr
12	104048432	chr12:104048432	G	A	1	0.00052	*STAB2*	missense variant	p.Arg503Gly
12	104078854	rs138445835	A	G	1	0.00052	*STAB2*	missense variant	p.Ala997Thr
12	104111602	chr12:104111602	T	A	1	0.00052	*STAB2*	missense variant	p.Ile1556Phe
12	108961086	chr12:108961086	T	C	1	0.00052	*ISCU*	stop gained	p.Gln154*
12	109041247	chr12:109041247	T	C	1	0.00052	*CORO1C*	missense variant	p.Asp506Asn
12	109541397	chr12:109541397	C	A	1	0.00052	*UNG*	missense variant	p.Lys261Thr

12	109945540	rs774203713	T	C	1	0.00052	*UBE3B*	missense variant & splice region variant	p.Thr541Ile
12	109949059	rs376213401	A	G	1	0.00052	*UBE3B*	missense variant	p.Arg636Gln
12	109972537	rs370843966	A	G	1	0.00057	*UBE3B*	missense variant	p.Glu1053Lys
12	111948294	chr12:111948294	C	G	1	0.00052	*ATXN2*	missense variant	p.Gln711Glu
12	111956136	rs755386197	A	G	1	0.00052	*ATXN2*	missense variant	p.Ser521Leu
12	111956139	chr12:111956139	T	C	1	0.00052	*ATXN2*	missense variant	p.Gly520Glu
12	112280935	chr12:112280935	G	A	1	0.00052	*MAPKAPK5*	missense variant	p.Tyr22Cys
12	112485570	chr12:112485570	C	T	1	0.00052	*NAA25*	missense variant	p.Ile635Met
12	122332723	rs552043284	T	C	1	0.00052	*PSMD9*	missense variant	p.Arg73Cys
12	123238315	rs780142224	G	T	1	0.00052	*DENR*	missense variant	p.Leu23Val
12	123434428	chr12:123434428	C	G	1	0.00054	*ABCB9*	missense variant	p.Leu252Val
12	123813442	rs757344135	G	A	1	0.00052	*SBNO1*	missense variant & splice region variant	p.Phe379Leu
12	129189764	rs777057937	T	C	1	0.00058	*TMEM132C*	missense variant	p.Arg751Cys
12	129299323	chr12:129299323	C	T	1	0.00052	*SLC15A4*	missense variant	p.Asn280Ser
13	21370341	chr13:21370341	C	T	1	0.00052	*XPO4*	missense variant	p.Lys891Glu
13	24330756	chr13:24330756	G	C	1	0.00056	*MIPEP*	missense variant & splice region variant	p.Ala658Pro
13	25672042	chr13:25672042	C	A	1	0.00053	*PABPC3*	missense variant	p.His569Pro
13	28626774	chr13:28626774	G	T	1	0.00052	*FLT3*	missense variant	p.Arg174Ser
13	28877500	rs763158497	T	C	1	0.00057	*FLT1*	missense variant	p.Arg1274Lys
13	28895671	rs754303774	T	C	1	0.00052	*FLT1*	missense variant	p.Val1035Met
13	31231679	chr13:31231679	T	G	1	0.00052	*USPL1*	missense variant	p.Ala489Ser
13	31231818	chr13:31231818	G	C	1	0.00053	*USPL1*	missense variant	p.Ser535Cys
13	37427768	rs757281925	T	C	1	0.00053	*SMAD9*	missense variant	p.Val350Met
13	41134585	chr13:41134585	G	T	1	0.00052	*FOXO1*	missense variant	p.Tyr348Ser
13	41517106	chr13:41517106	T	G	1	0.00052	*ELF1*	missense variant	p.Thr263Asn
13	41524074	rs753000405	G	C	1	0.00052	*ELF1*	missense variant	p.Val133Leu
13	41646921	chr13:41646921	A	G	1	0.00052	*WBP4*	missense variant	p.Ala164Thr
13	48830338	chr13:48830338	C	T	1	0.00052	*ITM2B*	missense variant	p.Ile91Thr
13	49086968	rs746852001	C	T	1	0.00052	*RCBTB2*	missense variant	p.His143Arg
13	50092236	chr13:50092236	T	C	1	0.00052	*PHF11*	missense variant	p.Ala136Val
13	76164099	chr13:76164099	T	C	1	0.00052	*UCHL3*	missense variant	p.Pro101Leu
13	76164252	chr13:76164252	A	G	1	0.00052	*UCHL3*	missense variant	p.Gly152Asp
13	76335124	chr13:76335124	C	G	1	0.00053	*LMO7*	missense variant	p.Gln141His
13	76397972	chr13:76397972	C	G	1	0.00052	*LMO7*	missense variant	p.Gly1023Ala
13	76407260	rs755067327	G	A	1	0.00052	*LMO7*	missense variant	p.Lys1060Arg
13	113512550	chr13:113512550	G	C	1	0.00052	*ATP11A*	missense variant	p.His871Gln
13	113556677	rs542171405	C	T	1	0.00052	*MCF2L*	missense variant	p.Met54Thr
13	114472131	chr13:114472131	G	A	1	0.00052	*TMEM255B*	missense variant & splice region variant	p.Met84Val
14	20876416	chr14:20876416	T	C	1	0.00052	*TEP1*	missense variant	p.Met61Ile
14	20897481	chr14:20897481	T	A	1	0.00054	*KLHL33*	missense variant	p.Cys377Ser
14	21487849	rs372825917	A	G	1	0.00052	*NDRG2*	missense variant	p.Thr222Ile
14	21511164	chr14:21511164	G	A	1	0.00055	*RNASE7*	missense variant	p.Arg5Gly
14	21511524	chr14:21511524	T	C	1	0.00052	*RNASE7*	stop gained	p.Arg125*
14	21992478	rs370096247	T	C	1	0.00052	*SALL2*	missense variant	p.Glu462Lys
14	22111727	chr14:22111727	G	C	1	0.00052	*TRAV1-2*	missense variant	p.Arg83Gly
14	22280017	chr14:22280017	G	C	1	0.00052	*TRAV9-1*	missense variant	p.Ala69Gly
14	22466287	chr14:22466287	C	G	1	0.00052	*TRAV17*	missense variant	p.Glu73Gln
14	23237365	chr14:23237365	A	G	1	0.00052	*OXA1L*	missense variant	p.Gly202Arg
14	23445709	chr14:23445709	T	C	1	0.00052	*AJUBA*	missense variant	p.Ala399Thr
14	23566904	chr14:23566904	A	T	1	0.00052	*C14orf119*	missense variant	p.Phe13Ile
14	23745607	rs73602454	A	G	1	0.00052	*HOMEZ*	missense variant	p.Ser279Leu
14	23856759	chr14:23856759	G	C	1	0.00054	*MYH6*	missense variant	p.Gln1543His
14	23894984	chr14:23894984	G	T	1	0.00052	*MYH7*	missense variant	p.Ile736Leu
14	24571998	rs766166255	G	T	1	0.00053	*PCK2*	missense variant	p.Phe424Cys
14	24588364	rs373798935	T	C	1	0.00052	*DCAF11*	missense variant	p.Arg264Cys
14	24739230	chr14:24739230	T	C	1	0.00056	*RABGGTA*	missense variant	p.Arg119His
14	24798400	rs752368778	G	A	1	0.00055	*ADCY4*	missense variant	p.Leu464Pro
14	24883816	chr14:24883816	C	T	1	0.00052	*NYNRIN*	missense variant	p.Ile954Thr
14	50307541	chr14:50307541	G	A	1	0.00052	*NEMF*	missense variant	p.Ile134Thr
14	50318558	chr14:50318558	C	A	1	0.00052	*NEMF*	missense variant	p.Val32Gly
14	55510109	rs771813922	T	C	1	0.00052	*SOCS4*	missense variant	p.Pro117Leu
14	62187288	chr14:62187288	T	C	1	0.00052	*HIF1A*	missense variant & splice region variant	p.Ala99Val
14	64681089	rs755527635	A	G	1	0.00056	*SYNE2*	missense variant	p.Val6412Met
14	64683131	chr14:64683131	T	C	1	0.00052	*SYNE2*	missense variant	p.Thr6442Ile

14	65527962	rs369004788	G	C	1	0.00052	*FNTB*	missense variant	p.Leu450Val
14	65550920	rs781393600	C	T	1	0.00052	*MAX*	missense variant	p.Lys90Arg
14	67850069	chr14:67850069	G	C	1	0.00052	*EIF2S1*	missense variant	p.Ala287Gly
14	70245141	chr14:70245141	C	G	1	0.00052	*SLC10A1*	missense variant	p.Phe284Leu
14	73980809	chr14:73980809	T	A	1	0.00052	*HEATR4*	missense variant	p.Leu447Gln
14	73989345	chr14:73989345	C	T	1	0.00053	*HEATR4*	missense variant	p.His171Arg
14	77705068	chr14:77705068	G	C	1	0.00052	*TMEM63C*	missense variant	p.Pro228Arg
14	94754645	rs779524045	G	A	1	0.00052	*SERPINA10*	missense variant	p.Trp364Arg
14	94756648	rs774696183	T	C	1	0.00057	*SERPINA10*	missense variant	p.Asp135Asn
14	99865078	chr14:99865078	G	T	1	0.00054	*SETD3*	missense variant	p.Lys575Gln
14	101349424	chr14:101349424	A	G	1	0.00053	*RTL1*	missense variant	p.Pro568Ser
14	102793048	chr14:102793048	A	G	1	0.00052	*ZNF839*	missense variant	p.Gly339Ser
14	102808331	chr14:102808331	A	G	1	0.00054	*ZNF839*	missense variant	p.Gly867Ser
14	103369720	rs747696299	T	G	1	0.00056	*TRAF3*	missense variant	p.Glu363Asp
14	105220205	chr14:105220205	T	A	1	0.00056	*SIVA1*	missense variant	p.Gln50Leu
14	106110159	rs370671336	A	C	1	0.00057	*IGHG2*	missense variant	p.Gln153His
15	34648801	rs762688120	T	G	1	0.00052	*NUTM1*	missense variant	p.Trp864Cys
15	38795473	rs780796375	T	A	1	0.00052	*RASGRP1*	missense variant & splice region variant	p.Asp527Glu
15	40585156	rs868163732	T	C	1	0.00053	*PLCB2*	missense variant	p.Arg766His
15	40587181	rs759781261	T	C	1	0.00052	*PLCB2*	stop gained	p.Trp621*
15	40595526	rs780036197	T	C	1	0.00052	*PLCB2*	missense variant	p.Arg65Gln
15	40685760	rs752195768	G	A	1	0.00052	*KNSTRN*	missense variant	p.Thr305Ala
15	41826966	chr15:41826966	T	C	1	0.00053	*RPAP1*	missense variant	p.Ala237Thr
15	41829299	chr15:41829299	T	C	1	0.00056	*RPAP1*	missense variant	p.Glu9Lys
15	44089095	chr15:44089095	A	G	1	0.00052	*RP11-296A16.1*	missense variant	p.Ala104Val
15	49509426	chr15:49509426	C	T	1	0.00052	*GALK2*	missense variant	p.Met61Thr
15	50884362	rs750446200	A	G	1	0.00052	*TRPM7*	missense variant	p.Pro1357Leu
15	51520033	chr15:51520033	C	T	1	0.00052	*CYP19A1*	missense variant	p.Ile132Val
15	51676027	chr15:51676027	A	G	1	0.00052	*GLDN*	missense variant	p.Gly160Glu
15	51696919	chr15:51696919	T	G	1	0.00052	*GLDN*	missense variant	p.Val542Leu
15	52181290	rs765884770	T	G	1	0.00052	*TMOD3*	missense variant	p.Lys148Asn
15	52258005	rs200556754	C	T	1	0.00052	*LEO1*	missense variant	p.Gln252Arg
15	55483191	rs747216897	G	C	1	0.00053	*RSL24D1*	missense variant	p.Glu84Gln
15	55647009	rs777721813	T	C	1	0.00052	*PIGB*	missense variant	p.Pro451Ser
15	59139640	rs776220227	C	T	1	0.00052	*FAM63B*	missense variant	p.Tyr505His
15	59445847	chr15:59445847	A	C	1	0.00052	*MYO1E*	missense variant	p.Val1008Leu
15	59455396	chr15:59455396	A	C	1	0.00052	*MYO1E*	stop gained	p.Glu863*
15	59455489	chr15:59455489	G	C	1	0.00052	*MYO1E*	missense variant	p.Asp832His
15	59528836	chr15:59528836	C	T	1	0.00052	*MYO1E*	missense variant	p.Lys123Arg
15	60648141	chr15:60648141	C	T	1	0.00052	*ANXA2*	missense variant	p.Lys187Glu
15	68445937	rs772361559	G	A	1	0.00052	*PIAS1*	missense variant	p.Met282Val
15	69076852	chr15:69076852	G	T	1	0.00052	*ANP32A*	missense variant	p.Lys137Thr
15	74636307	rs773957723	A	G	1	0.00053	*CYP11A1*	missense variant	p.Arg218Cys
15	75656960	rs766243508	T	C	1	0.00054	*MAN2C1*	missense variant	p.Gly157Arg
15	75668202	rs556160377	T	C	1	0.00052	*SIN3A*	missense variant	p.Arg1132Gln
15	83826130	chr15:83826130	C	A	1	0.00052	*HDGFRP3*	missense variant	p.Cys141Gly
16	3073477	rs369375533	G	C	1	0.00056	*HCFC1R1*	missense variant & splice region variant	p.Glu50Asp
16	3076523	chr16:3076523	C	T	1	0.00052	*THOC6*	missense variant	p.Ser140Pro
16	3188362	chr16:3188362	C	A	1	0.00052	*ZNF213*	missense variant	p.Asn136Thr
16	3199011	chr16:3199011	A	G	1	0.00053	*CASP16*	missense variant	p.Gly137Arg
16	3367541	chr16:3367541	A	C	1	0.00052	*ZNF75A*	missense variant	p.Pro188His
16	11137925	chr16:11137925	T	A	1	0.00052	*CLEC16A*	missense variant	p.Tyr604Phe
16	11525057	chr16:11525057	A	C	1	0.00052	*CTD-3088G3.8*	missense variant	p.Asp1777Tyr
16	22128183	chr16:22128183	A	C	1	0.00052	*VWA3A*	missense variant	p.Pro307Thr
16	22268697	rs750035573	A	G	1	0.00052	*EEF2K*	missense variant	p.Gly298Ser
16	22269077	chr16:22269077	T	A	1	0.00055	*EEF2K*	missense variant	p.Asn339Tyr
16	22360729	chr16:22360729	G	A	1	0.00052	*CDR2*	missense variant	p.Ile126Thr
16	27374667	rs372491173	T	C	1	0.00057	*IL4R*	missense variant	p.Pro665Leu
16	27517389	chr16:27517389	A	G	1	0.00053	*GTF3C1*	missense variant	p.Ser534Phe
16	28842289	chr16:28842289	G	C	1	0.00052	*ATXN2L*	missense variant	p.Ser406Cys
16	28890023	chr16:28890023	A	G	1	0.00053	*ATP2A1*	missense variant	p.Glu11Lys
16	28944716	rs781369027	T	G	1	0.00052	*CD19*	missense variant	p.Val241Leu
16	30122719	chr16:30122719	C	T	1	0.00052	*GDPD3*	missense variant	p.Ile233Val
16	32264862	chr16:32264862	C	A	1	0.00055	*TP53TG3D*	missense variant	p.Gln63Pro
16	50745281	chr16:50745281	C	G	1	0.00058	*NOD2*	missense variant	p.Val487Leu
16	50827504	chr16:50827504	G	A	1	0.00052	*CYLD*	missense variant	p.Arg800Gly
16	50830312	chr16:50830312	A	G	1	0.00052	*CYLD*	missense variant	p.Asp922Asn

16	56868349	chr16:56868349	T	C	1	0.00052	*NUP93*	stop gained	p.Arg578*
16	56872886	rs190565265	A	G	1	0.00052	*NUP93*	missense variant	p.Ala681Thr
16	66420908	chr16:66420908	A	G	1	0.00052	*CDH5*	missense variant	p.Ser136Asn
16	68338035	chr16:68338035	C	T	1	0.00052	*SLC7A6OS*	missense variant	p.Gln191Arg
16	68842660	chr16:68842660	A	C	1	0.00052	*CDH1*	missense variant	p.Thr199Lys
16	68857386	chr16:68857386	G	A	1	0.00052	*CDH1*	missense variant	p.Asn674Ser
16	69727203	chr16:69727203	G	T	1	0.00052	*NFAT5*	missense variant	p.Phe1159Val
16	72132587	chr16:72132587	G	T	1	0.00052	*DHX38*	missense variant	p.Ser220Arg
16	72133695	rs758566056	G	A	1	0.00053	*DHX38*	missense variant	p.Tyr342Cys
16	72144861	rs753686955	T	C	1	0.00058	*DHX38*	missense variant	p.Arg1162Cys
16	72170687	chr16:72170687	T	C	1	0.00052	*PMFBP1*	missense variant	p.Ser317Asn
16	81957177	rs781130389	A	G	1	0.00053	*PLCG2*	missense variant	p.Val799Ile
16	81971363	chr16:81971363	G	A	1	0.00052	*PLCG2*	missense variant & splice region variant	p.Asp1018Gly
16	82033159	rs756402487	T	C	1	0.00052	*SDR42E1*	missense variant	p.Ala247Thr
16	82033534	chr16:82033534	C	T	1	0.00052	*SDR42E1*	missense variant	p.Ser122Gly
17	3629460	chr17:3629460	C	G	1	0.00052	*GSG2*	missense variant	p.Trp744Ser
17	3970509	chr17:3970509	T	C	1	0.00052	*ZZEF1*	missense variant	p.Ser1368Asn
17	4074042	rs751428903	T	C	1	0.00052	*ANKFY1*	missense variant	p.Val1127Ile
17	4621619	rs376221990	A	G	1	0.00052	*ARRB2*	missense variant	p.Val250Ile
17	4647969	rs780614708	A	C	1	0.00057	*ZMYND15*	missense variant	p.Pro578His
17	4836257	rs780652188	A	G	1	0.00058	*GP1BA*	missense variant	p.Val120Ile
17	4836714	chr17:4836714	C	T	1	0.00052	*GP1BA*	missense variant	p.Val272Ala
17	4850115	chr17:4850115	A	G	1	0.00052	*PFN1*	missense variant & splice region variant	p.Pro124Ser
17	4858827	chr17:4858827	A	G	1	0.00052	*ENO3*	missense variant	p.Asp265Asn
17	4859893	rs778070292	G	T	1	0.00052	*ENO3*	missense variant	p.Trp365Gly
17	4881889	chr17:4881889	G	C	1	0.00052	*CAMTA2*	missense variant	p.Glu582Gln
17	4904518	rs762688180	T	C	1	0.00052	*KIF1C*	missense variant & splice region variant	p.Thr62Met
17	5337008	rs748497469	G	C	1	0.00052	*C1QBP*	missense variant	p.Cys186Ser
17	7495195	chr17:7495195	A	G	1	0.00055	*FXR2*	missense variant	p.Leu659Phe
17	7559181	chr17:7559181	A	G	1	0.00053	*ATP1B2*	missense variant	p.Val281Met
17	7592927	rs776832907	G	A	1	0.00052	*WRAP53*	missense variant	p.Ile184Val
17	7641314	chr17:7641314	A	G	1	0.00052	*DNAH2*	missense variant	p.Ala402Thr
17	11837306	rs143624582	A	G	1	0.00052	*DNAH9*	missense variant	p.Arg4136Gln
17	19250493	chr17:19250493	G	A	1	0.00052	*B9D1*	missense variant	p.Ser141Pro
17	21216804	rs771434728	A	C	1	0.00057	*MAP2K3*	stop gained & splice region variant	p.Cys305*
17	25973582	chr17:25973582	G	A	1	0.00053	*LGALS9*	missense variant	p.Ile213Val
17	26089860	rs759222198	A	G	1	0.00055	*NOS2*	missense variant	p.His922Tyr
17	26114833	chr17:26114833	A	T	1	0.00052	*NOS2*	missense variant	p.Lys113Ile
17	33591242	chr17:33591242	G	C	1	0.00052	*SLFN5*	stop gained	p.Tyr393*
17	34191275	chr17:34191275	G	A	1	0.00052	*C17orf66*	missense variant	p.Phe181Ser
17	34431333	chr17:34431333	G	T	1	0.00052	*CCL4*	missense variant	p.Met12Arg
17	36454466	chr17:36454466	T	C	1	0.00052	*MRPL45*	missense variant	p.Arg40Cys
17	36628240	chr17:36628240	G	A	1	0.00053	*ARHGAP23*	missense variant	p.Lys704Arg
17	37825912	rs755016927	C	T	1	0.00057	*PNMT*	missense variant	p.Ile78Thr
17	37948943	chr17:37948943	C	T	1	0.00052	*IKZF3*	missense variant	p.His136Arg
17	38251356	chr17:38251356	G	C	1	0.00053	*NR1D1*	missense variant	p.Met422Ile
17	38433379	rs762490491	A	C	1	0.00052	*WIPF2*	missense variant	p.Pro409Thr
17	38787050	chr17:38787050	A	G	1	0.00052	*SMARCE1*	stop gained	p.Gln315*
17	38813616	chr17:38813616	C	T	1	0.00052	*KRT222*	missense variant	p.His216Arg
17	38816383	chr17:38816383	G	A	1	0.00052	*KRT222*	missense variant	p.Ile101Thr
17	40155571	rs782268381	G	T	1	0.00053	*DNAJC7*	missense variant	p.Thr28Pro
17	40257809	rs201235598	T	C	1	0.00056	*DHX58*	missense variant	p.Arg399Gln
17	40257975	chr17:40257975	A	G	1	0.00055	*DHX58*	missense variant	p.His344Tyr
17	40260008	chr17:40260008	G	C	1	0.00055	*DHX58*	missense variant	p.Ser266Thr
17	40270392	chr17:40270392	A	T	1	0.00054	*KAT2A*	missense variant	p.Tyr368Phe
17	40271354	rs782687964	T	C	1	0.00052	*KAT2A*	missense variant	p.Val328Ile
17	40376852	chr17:40376852	T	C	1	0.00052	*STAT5B*	missense variant	p.Arg107His
17	40842136	rs147189145	A	G	1	0.00052	*CNTNAP1*	missense variant	p.Arg589Gln
17	41180612	rs143507179	A	G	1	0.00057	*RND2*	missense variant	p.Arg201Gln
17	41246104	chr17:41246104	A	T	1	0.00052	*BRCA1*	missense variant	p.Ile482Phe
17	43316428	chr17:43316428	C	T	1	0.00052	*FMNL1*	missense variant	p.Leu359Ser
17	45820084	chr17:45820084	C	G	1	0.00057	*TBX21*	missense variant	p.Gln200His
17	45820439	rs768130869	G	A	1	0.00054	*TBX21*	missense variant & splice region variant	p.Asn217Asp
17	45915629	rs758226121	T	G	1	0.00055	*SCRN2*	missense variant	p.Pro376Thr

17	45916930	rs773195495	A	C	1	0.00055	*SCRN2*	missense variant	p.Gly154Cys
17	47018301	chr17:47018301	T	C	1	0.00052	*SNF8*	missense variant	p.Val77Met
17	48245089	chr17:48245089	T	G	1	0.00054	*SGCA*	missense variant	p.Val102Phe
17	48940763	chr17:48940763	A	G	1	0.00052	*TOB1*	missense variant	p.Pro206Ser
17	49156947	chr17:49156947	C	T	1	0.00052	*SPAG9*	missense variant & splice region variant	p.Gln141Arg
17	54912595	chr17:54912595	A	T	1	0.00052	*DGKE*	missense variant	p.Cys147Ser
17	54978936	chr17:54978936	T	C	1	0.00053	*TRIM25*	missense variant	p.Ala311Thr
17	55058579	chr17:55058579	T	C	1	0.00052	*SCPEP1*	missense variant	p.His60Tyr
17	56058153	chr17:56058153	C	T	1	0.00052	*VEZF1*	missense variant	p.Met81Val
17	56232756	chr17:56232756	A	T	1	0.00052	*OR4D1*	missense variant	p.Met81Lys
17	56434758	chr17:56434758	A	C	1	0.00053	*RNF43*	missense variant	p.Lys666Asn
17	58040502	chr17:58040502	G	A	1	0.00052	*RNFT1*	missense variant	p.Leu67Ser
17	61829344	chr17:61829344	A	G	1	0.00052	*RP11-51F16.8*	stop gained	p.Arg38*
17	61895253	chr17:61895253	T	A	1	0.00053	*DDX42*	missense variant	p.Asn771Ile
17	61895456	rs556300636	T	C	1	0.00055	*DDX42*	missense variant	p.Arg839Cys
17	61908463	rs534233529	T	C	1	0.00052	*PSMC5*	stop gained	p.Arg109*
17	64876683	rs138881080	A	G	1	0.00052	*CACNG5*	missense variant	p.Arg98His
17	72540951	rs759032539	G	T	1	0.00052	*CD300C*	missense variant	p.Asp66Ala
17	73231295	rs768393987	G	A	1	0.00052	*NUP85*	missense variant & splice region variant	p.Gln623Arg
17	73234695	chr17:73234695	A	G	1	0.00054	*GGA3*	missense variant	p.Pro74Leu
17	73258891	chr17:73258891	A	C	1	0.00052	*MRPS7*	missense variant	p.Phe123Leu
17	73727019	rs778104417	A	G	1	0.00053	*ITGB4*	missense variant	p.Val356Met
17	73727044	rs370095218	G	A	1	0.00054	*ITGB4*	missense variant & splice region variant	p.Asn364Ser
17	74622810	rs138031739	T	C	1	0.00053	*ST6GALNAC1*	missense variant	p.Gly412Ser
17	76421538	chr17:76421538	T	C	1	0.00053	*DNAH17*	missense variant	p.Glu4367Lys
17	76831448	rs148192093	C	G	1	0.00053	*USP36*	missense variant	p.Thr130Ser
17	76968019	chr17:76968019	C	T	1	0.00052	*LGALS3BP*	missense variant	p.His466Arg
17	76993315	chr17:76993315	C	T	1	0.00053	*CANT1*	missense variant	p.Lys77Glu
18	670767	rs373096647	T	A	1	0.00052	*TYMS*	missense variant	p.Gln211Leu
18	743022	chr18:743022	G	T	1	0.00052	*YES1*	missense variant	p.Gln324Pro
18	2891408	chr18:2891408	A	G	1	0.00052	*EMILIN2*	missense variant	p.Gly428Glu
18	2891449	chr18:2891449	A	C	1	0.00052	*EMILIN2*	missense variant	p.Pro442Thr
18	8406128	chr18:8406128	A	G	1	0.00052	*PTPRM*	missense variant	p.Glu1457Lys
18	9256796	chr18:9256796	T	G	1	0.00052	*ANKRD12*	missense variant	p.Lys1177Asn
18	12686304	rs762367925	C	T	1	0.00052	*CEP76*	missense variant	p.Gln360Arg
18	13885084	chr18:13885084	T	C	1	0.00054	*MC2R*	missense variant	p.Arg145His
18	18559886	rs775538267	C	T	1	0.00053	*ROCK1*	missense variant	p.Glu880Gly
18	48581159	chr18:48581159	G	A	1	0.00052	*SMAD4*	missense variant	p.Ser155Gly
18	54362397	chr18:54362397	G	A	1	0.00052	*WDR7*	missense variant	p.Gln442Arg
18	55335743	chr18:55335743	G	C	1	0.00052	*ATP8B1*	missense variant	p.Lys709Asn
19	3735930	rs565934761	A	G	1	0.00052	*TJP3*	missense variant	p.Arg408His
19	4212678	rs375444482	T	C	1	0.00056	*ANKRD24*	missense variant	p.Arg484Trp
19	6380353	chr19:6380353	A	T	1	0.00052	*GTF2F1*	missense variant	p.Lys498Met
19	6467946	chr19:6467946	C	T	1	0.00053	*DENND1C*	missense variant	p.Thr659Ala
19	6468372	chr19:6468372	C	T	1	0.00056	*DENND1C*	missense variant	p.Glu555Gly
19	6697719	rs771305132	T	C	1	0.00053	*C3*	missense variant	p.Glu843Lys
19	6719341	rs780548430	C	G	1	0.00057	*C3*	missense variant	p.Gln50Glu
19	6735921	chr19:6735921	G	A	1	0.00052	*GPR108*	missense variant & splice region variant	p.Ser97Pro
19	6832126	rs144739934	A	G	1	0.00052	*VAV1*	missense variant	p.Glu475Lys
19	7686101	rs777686042	T	C	1	0.00056	*XAB2*	missense variant	p.Arg567His
19	7689210	rs763248298	A	G	1	0.00059	*XAB2*	missense variant	p.Ser315Leu
19	7695745	chr19:7695745	G	T	1	0.00053	*PET100*	missense variant	p.Phe51Val
19	7712634	chr19:7712634	G	A	1	0.00056	*STXBP2*	missense variant	p.Thr585Ala
19	7743449	rs757755213	C	T	1	0.00052	*C19orf59*	missense variant	p.Met149Thr
19	7810715	rs745378205	G	A	1	0.00054	*CD209*	missense variant	p.Ile146Thr
19	10091356	rs368541624	A	G	1	0.00053	*COL5A3*	stop gained	p.Gln858*
19	10257109	rs770079133	C	G	1	0.00053	*DNMT1*	missense variant	p.Leu938Val
19	10395926	chr19:10395926	T	C	1	0.00053	*ICAM1*	missense variant	p.Thr521Ile
19	10812642	chr19:10812642	G	A	1	0.00054	*QTRT1*	missense variant	p.Gln87Arg
19	11033825	rs750963587	A	G	1	0.00055	*YIPF2*	missense variant	p.Ser188Phe
19	11118683	chr19:11118683	A	G	1	0.00053	*SMARCA4*	missense variant	p.Ala703Thr
19	11332620	chr19:11332620	T	C	1	0.00056	*DOCK6*	missense variant	p.Ala1153Thr
19	12126310	chr19:12126310	T	G	1	0.00052	*ZNF433*	missense variant	p.Pro458Thr
19	12841792	chr19:12841792	A	G	1	0.00053	*C19orf43*	missense variant	p.Arg172Trp
19	12858827	rs774637174	T	C	1	0.00054	*ASNA1*	missense variant	p.Pro319Leu

19	12981975	chr19:12981975	T	G	1	0.00055	*MAST1*	missense variant	p.Glu1084Asp
19	14200056	rs563414303	G	C	1	0.00053	*SAMD1*	missense variant	p.Gly252Ala
19	16268042	rs75384630	A	G	1	0.00054	*HSH2D*	missense variant	p.Cys166Tyr
19	17394286	chr19:17394286	T	C	1	0.00056	*ANKLE1*	missense variant	p.Pro238Leu
19	17892605	chr19:17892605	T	C	1	0.00056	*FCHO1*	missense variant	p.Ala645Val
19	18218419	chr19:18218419	A	G	1	0.00055	*MAST3*	missense variant	p.Arg21Gln
19	18256001	rs761394244	A	G	1	0.00056	*MAST3*	missense variant	p.Gly972Ser
19	18474257	rs771517091	G	C	1	0.00053	*PGPEP1*	missense variant	p.Ala165Gly
19	18965798	chr19:18965798	C	T	1	0.00057	*UPF1*	missense variant	p.Leu470Pro
19	35775938	rs767080313	G	A	1	0.00052	*HAMP*	missense variant	p.Lys83Arg
19	35802832	chr19:35802832	G	C	1	0.00055	*MAG*	missense variant	p.Thr543Arg
19	35832254	chr19:35832254	G	C	1	0.00055	*CD22*	missense variant	p.Arg506Gly
19	36302916	chr19:36302916	A	G	1	0.00057	*PRODH2*	missense variant	p.Ala258Val
19	36332658	chr19:36332658	T	G	1	0.00052	*NPHS1*	missense variant	p.Ala925Asp
19	38980762	rs775643457	A	G	1	0.00055	*RYR1*	missense variant	p.Arg1954His
19	39001174	rs768538971	T	C	1	0.00052	*RYR1*	missense variant	p.Pro2990Leu
19	39307959	rs757032356	T	C	1	0.00057	*ECH1*	missense variant	p.Gly174Arg
19	39439250	rs763629297	T	C	1	0.00054	*CTC-360G5.8*	missense variant	p.Val44Met
19	39884243	rs771903804	G	T	1	0.00052	*MED29*	missense variant	p.Phe130Cys
19	39975837	chr19:39975837	G	C	1	0.00054	*TIMM50*	missense variant	p.Ser110Trp
19	39977097	rs766277051	T	C	1	0.00053	*TIMM50*	missense variant	p.Arg323Trp
19	40097959	rs193269928	C	T	1	0.00052	*LGALS13*	missense variant	p.Ser134Pro
19	41826311	chr19:41826311	A	G	1	0.00055	*CCDC97*	missense variant	p.Glu283Lys
19	42584038	chr19:42584038	T	C	1	0.00058	*ZNF574*	missense variant	p.Pro517Leu
19	49207142	chr19:49207142	A	C	1	0.00052	*FUT2*	missense variant	p.Thr310Asn
19	49376671	chr19:49376671	C	G	1	0.00054	*PPP1R15A*	missense variant	p.Glu61Gln
19	49477932	chr19:49477932	T	A	1	0.00054	*GYS1*	missense variant	p.Ile456Asn
19	49930177	chr19:49930177	A	G	1	0.00052	*GFY*	missense variant	p.Glu164Lys
19	49989350	chr19:49989350	A	G	1	0.00052	*FLT3LG*	missense variant	p.Gly151Asp
19	50004387	chr19:50004387	A	G	1	0.00059	*hsa-mir-150*	missense variant	p.Gly203Arg
19	50138844	chr19:50138844	G	C	1	0.00056	*RRAS*	missense variant	p.Val216Leu
19	50165277	rs368333462	T	C	1	0.00056	*IRF3*	missense variant	p.Gly304Arg
19	50170397	rs751409023	T	C	1	0.00052	*BCL2L12*	missense variant	p.Pro161Ser
19	50804997	rs777220522	A	G	1	0.00056	*MYH14*	missense variant	p.Arg1850Gln
19	50940957	rs750573077	C	G	1	0.00052	*MYBPC2*	missense variant	p.Ser185Thr
19	53208747	chr19:53208747	A	G	1	0.00052	*ZNF611*	missense variant	p.Arg521Cys
19	54299219	rs770386298	T	G	1	0.00052	*NLRP12*	missense variant	p.Gln999Lys
19	54848366	chr19:54848366	C	A	1	0.00058	*LILRA4*	missense variant	p.Val334Gly
19	55019328	chr19:55019328	C	T	1	0.00052	*LAIR2*	missense variant	p.Leu98Pro
19	55420766	rs759022000	T	C	1	0.00054	*NCR1*	missense variant	p.Ala173Val
19	55493915	chr19:55493915	G	C	1	0.00054	*NLRP2*	missense variant	p.Ile283Met
19	55501899	chr19:55501899	C	A	1	0.00052	*NLRP2*	missense variant	p.Asn856Thr
19	56189019	rs762919490	G	C	1	0.00052	*EPN1*	missense variant	p.Leu51Val
19	56249609	rs370177421	G	C	1	0.00052	*NLRP9*	missense variant	p.Trp44Cys
19	56369414	chr19:56369414	G	A	1	0.00052	*NLRP4*	missense variant	p.Ile219Val
19	56549541	chr19:56549541	C	G	1	0.00052	*NLRP5*	missense variant	p.Gln922His
20	1209111	chr20:1209111	G	A	1	0.00052	*RAD21L1*	missense variant	p.His5Arg
20	2636276	chr20:2636276	A	G	1	0.00052	*NOP56*	missense variant	p.Glu265Lys
20	2637178	rs758338406	T	G	1	0.00053	*NOP56*	missense variant	p.Arg381Leu
20	5924282	rs762857318	G	A	1	0.00052	*TRMT6*	missense variant	p.Ile197Thr
20	5996127	chr20:5996127	A	C	1	0.00052	*CRLS1*	missense variant	p.Leu189Ile
20	18474669	chr20:18474669	C	T	1	0.00052	*RBBP9*	missense variant	p.Thr61Ala
20	35128750	chr20:35128750	T	C	1	0.00056	*DLGAP4*	missense variant	p.Arg750Trp
20	36999931	chr20:36999931	A	G	1	0.00052	*LBP*	missense variant	p.Val409Met
20	39796502	chr20:39796502	G	A	1	0.00052	*PLCG1*	missense variant	p.Tyr771Cys
20	39833241	chr20:39833241	G	C	1	0.00052	*ZHX3*	missense variant	p.Asp106His
20	43043264	chr20:43043264	C	T	1	0.00055	*HNF4A*	missense variant	p.Tyr204His
20	44334528	rs770731996	C	T	1	0.00052	*WFDC13*	missense variant	p.Ile89Thr
20	44521413	rs752113422	T	C	1	0.00052	*CTSA*	missense variant	p.Pro183Leu
20	44523364	chr20:44523364	T	G	1	0.00053	*CTSA*	missense variant	p.Val303Leu
20	44523736	chr20:44523736	G	A	1	0.00056	*CTSA*	missense variant	p.Asn369Ser
20	44527063	rs147472210	T	C	1	0.00055	*CTSA*	missense variant	p.Arg491Cys
20	44533657	chr20:44533657	A	G	1	0.00055	*PLTP*	missense variant	p.Ser289Phe
20	44538288	chr20:44538288	T	C	1	0.00053	*PLTP*	missense variant	p.Ala138Thr
20	44669120	chr20:44669120	T	A	1	0.00052	*SLC12A5*	missense variant	p.Met264Leu
20	44698964	chr20:44698964	G	C	1	0.00052	*NCOA5*	missense variant	p.Val84Leu
20	44698973	rs746747724	T	C	1	0.00052	*NCOA5*	missense variant	p.Val81Ile
20	46276064	rs370656650	G	C	1	0.00052	*NCOA3*	missense variant	p.Pro1167Arg
20	46277774	chr20:46277774	C	T	1	0.00052	*NCOA3*	missense variant	p.Leu1191Pro

20	47628572	rs767937591	T	C	1	0.00052	*ARFGEF2*	missense variant	p.Ala1290Val
20	47691971	rs754442648	A	G	1	0.00052	*CSE1L*	missense variant	p.Ala417Thr
20	47736550	chr20:47736550	A	G	1	0.00052	*STAU1*	missense variant	p.Pro361Leu
20	48524826	chr20:48524826	T	C	1	0.00055	*SPATA2*	missense variant	p.Glu68Lys
20	49211907	rs768291837	G	C	1	0.00052	*FAM65C*	missense variant & splice region variant	p.Glu683Asp
20	49236613	chr20:49236613	G	A	1	0.00052	*FAM65C*	missense variant	p.Met60Thr
20	50400814	rs373555761	A	G	1	0.00052	*SALL4*	missense variant	p.Ala1051Val
20	61488954	rs528649129	A	C	1	0.00053	*TCFL5*	missense variant	p.Arg344Leu
20	61538566	rs758731690	C	T	1	0.00052	*DIDO1*	missense variant	p.Lys436Arg
20	61542285	rs768637738	A	G	1	0.00053	*DIDO1*	missense variant	p.Ser227Phe
20	62224435	rs776946146	G	A	1	0.00052	*GMEB2*	missense variant & splice region variant	p.Val207Ala
21	26961170	chr21:26961170	T	G	1	0.00052	*MRPL39*	missense variant	p.Leu316Ile
21	26976136	chr21:26976136	C	T	1	0.00053	*MRPL39*	missense variant	p.Lys131Arg
21	33043893	chr21:33043893	G	C	1	0.00052	*SCAF4*	missense variant	p.Gly1088Ala
21	33063237	rs775209963	A	C	1	0.00052	*SCAF4*	missense variant	p.Lys586Asn
21	33065690	rs753270057	T	C	1	0.00052	*SCAF4*	missense variant	p.Arg477Gln
21	33692919	chr21:33692919	A	G	1	0.00052	*URB1*	missense variant	p.Ala1839Val
21	33711108	chr21:33711108	A	G	1	0.00052	*URB1*	missense variant	p.Pro1473Leu
21	33757904	rs114003733	C	A	1	0.00052	*URB1*	missense variant	p.Cys78Gly
21	34029111	chr21:34029111	T	C	1	0.00052	*SYNJ1*	missense variant	p.Gly933Asp
21	34115608	chr21:34115608	C	T	1	0.00053	*PAXBP1*	missense variant	p.Asn762Ser
21	34793899	rs756119057	G	A	1	0.00052	*IFNGR2*	missense variant	p.Met126Val
21	34883568	chr21:34883568	A	G	1	0.00052	*GART*	stop gained	p.Arg769*
21	34951771	chr21:34951771	T	G	1	0.00052	*DONSON*	stop gained	p.Ser483*
21	35140120	chr21:35140120	G	A	1	0.00052	*ITSN1*	missense variant	p.Lys344Glu
21	35147336	chr21:35147336	C	G	1	0.00052	*ITSN1*	missense variant	p.Arg507Thr
21	40552306	rs751396324	C	T	1	0.00052	*PSMG1*	missense variant	p.Lys100Glu
21	40670370	chr21:40670370	A	G	1	0.00052	*BRWD1*	missense variant	p.Arg113Cys
21	40795356	rs753208196	A	T	1	0.00054	*LCA5L*	missense variant	p.Gln128Leu
21	42749774	chr21:42749774	G	T	1	0.00058	*MX2*	missense variant	p.Leu103Arg
21	42754475	rs764566059	A	G	1	0.00055	*MX2*	missense variant	p.Arg239Gln
21	42809035	rs749472574	C	T	1	0.00052	*MX1*	missense variant	p.Ile131Thr
21	43770070	chr21:43770070	G	T	1	0.00056	*TFF2*	missense variant	p.Asp50Ala
21	43838576	chr21:43838576	C	T	1	0.00052	*UBASH3A*	missense variant	p.Tyr302His
21	43905847	rs778786028	T	G	1	0.00052	*RSPH1*	missense variant	p.Gln145Lys
21	45103166	rs755653178	A	G	1	0.00052	*RRP1B*	missense variant	p.Gly268Asp
21	45479062	chr21:45479062	G	T	1	0.00052	*TRAPPC10*	missense variant	p.Phe253Val
21	45507594	chr21:45507594	T	G	1	0.00053	*TRAPPC10*	missense variant	p.Ala852Ser
21	45655275	rs569961030	C	T	1	0.00055	*ICOSLG*	missense variant	p.Met193Val
21	46226843	rs368636514	A	G	1	0.00055	*SUMO3*	missense variant	p.Arg129Cys
22	18138586	rs773884030	C	T	1	0.00052	*BCL2L13*	missense variant	p.Ser37Pro
22	19506390	rs778665661	T	C	1	0.00053	*CDC45*	missense variant	p.Arg586Trp
22	19707965	chr22:19707965	G	C	1	0.00058	*SEPT5*	missense variant	p.Pro171Arg
22	20457733	chr22:20457733	T	C	1	0.00052	*RIMBP3*	missense variant	p.Arg1190Gln
22	21167698	chr22:21167698	T	G	1	0.00052	*PI4KA*	missense variant	p.Thr318Lys
22	21384134	rs371746371	G	C	1	0.00054	*SLC7A4*	missense variant	p.Val497Leu
22	22312884	chr22:22312884	T	G	1	0.00056	*TOP3B*	missense variant	p.Pro696His
22	24437627	chr22:24437627	T	C	1	0.00052	*CABIN1*	missense variant	p.Pro84Leu
22	24492045	rs745725059	G	A	1	0.00052	*CABIN1*	missense variant & splice region variant	p.Lys1313Arg
22	26895183	rs751102347	C	T	1	0.00052	*TFIP11*	missense variant	p.Thr406Ala
22	30057288	rs753300935	T	C	1	0.00052	*NF2*	missense variant	p.Pro257Leu
22	30189580	rs775679720	T	C	1	0.00058	*ASCC2*	missense variant	p.Val592Met
22	30508498	chr22:30508498	A	C	1	0.00053	*HORMAD2*	missense variant	p.Pro110His
22	30866505	chr22:30866505	A	C	1	0.00052	*SEC14L3*	missense variant	p.Arg40Leu
22	32498054	chr22:32498054	T	C	1	0.00052	*SLC5A1*	missense variant	p.Arg499Cys
22	37865907	chr22:37865907	G	A	1	0.00054	*MFNG*	missense variant	p.Ile71Thr
22	40283748	rs766301195	A	G	1	0.00052	*ENTHD1*	missense variant	p.Ala2Val
22	41660778	chr22:41660778	G	A	1	0.00056	*RANGAP1*	missense variant	p.Phe124Leu
22	42262870	rs752927725	C	T	1	0.00052	*SREBF2*	missense variant	p.Phe42Leu
22	46654517	chr22:46654517	T	G	1	0.00052	*PKDREJ*	missense variant	p.Pro1568His
22	50279232	chr22:50279232	C	T	1	0.00053	*ZBED4*	missense variant	p.Phe641Ser
22	50279355	chr22:50279355	G	A	1	0.00052	*ZBED4*	missense variant	p.Asn682Ser
22	50488649	chr22:50488649	A	G	1	0.00052	*TTLL8*	missense variant	p.His63Tyr
22	50678670	chr22:50678670	T	C	1	0.00054	*TUBGCP6*	missense variant	p.Glu290Lys
22	50682428	chr22:50682428	T	C	1	0.00052	*TUBGCP6*	missense variant	p.Ser154Asn
23	12939607	chrX:12939607	A	T	1	0.00056	*TLR8*	missense variant	p.Ser834Arg

23	30322733	chrX:30322733	C	T	1	0.00056	*NR0B1*	missense variant	p.Asp459Gly
23	38535020	rs201317934	G	A	1	0.00056	*TM4SF2*	missense variant	p.His198Arg
23	41060529	rs773181993	G	A	1	0.00056	*USP9X*	missense variant	p.Asn1607Ser
23	47517190	chrX:47517190	A	C	1	0.00056	*UXT*	missense variant	p.Glu69Asp
23	49032152	chrX:49032152	A	T	1	0.00058	*PRICKLE3*	missense variant	p.His585Leu
23	49050780	chrX:49050780	T	C	1	0.00057	*SYP*	missense variant	p.Gly89Glu
23	70787374	chrX:70787374	A	G	1	0.00056	*OGT*	missense variant	p.Val872Ile
23	77388925	chrX:77388925	G	C	1	0.00056	*TAF9B*	missense variant	p.Val168Leu
23	100537441	chrX:100537441	A	T	1	0.00057	*TAF7L*	missense variant & splice region variant	p.Met180Leu
23	101912859	chrX:101912859	T	G	1	0.00057	*GPRASP1*	missense variant	p.Ala1340Ser
23	101970975	rs775866009	T	C	1	0.00056	*GPRASP2*	missense variant	p.Ala393Val
23	107316048	chrX:107316048	C	T	1	0.00056	*VSIG1*	missense variant	p.Val221Ala
23	107433643	rs200541162	T	G	1	0.00056	*COL4A6*	missense variant	p.Leu470Ile
23	107811862	rs779822354	T	C	1	0.00056	*COL4A5*	missense variant	p.Pro94Ser
23	107976892	chrX:107976892	C	T	1	0.00058	*IRS4*	missense variant	p.Ile895Val
23	114141252	rs201583846	C	T	1	0.00056	*HTR2C*	missense variant	p.Trp186Arg
23	117895129	chrX:117895129	A	C	1	0.00057	*IL13RA1*	missense variant	p.Asn235Lys
23	123515051	rs774434990	A	G	1	0.00056	*TENM1*	missense variant	p.Arg2512Trp
23	129171505	rs368783964	T	C	1	0.00062	*BCORL1*	missense variant	p.Thr1564Met
23	153699965	rs781972979	G	C	1	0.00059	*PLXNA3*	missense variant	p.Thr1835Ser
23	153774301	chrX:153774301	T	C	1	0.00057	*G6PD*	missense variant	p.Asp24Asn
23	153944424	rs147290916	T	C	1	0.00057	*GAB3*	missense variant	p.Val85Met
23	154009930	chrX:154009930	A	G	1	0.00056	*MPP1*	missense variant	p.Thr365Ile

Supplementary Table S12. Clinical data for the five Swedish SLE patients carrying the *MUC5B* rs773068050 missense case-only allele.

	SLE1	SLE2	SLE3	SLE4	SLE5
Gender	Female	Female	Male	Female	Female
Age at diagnosis	30	43	50	27	48
Age at follow-up	34	77	50	33	64
≥4 ACR criteria (Tan et al., 1982; Hochberg et al., 1997)	YES	YES	YES	YES	YES
Number of ACR criteria fulfilled	7	4	7	4	5
ACR criteria:					
1) Malar rash	YES	YES	NO	NO	NO
2) Discoid rash	NO	NO	NO	NO	NO
3) Photosensitivity	YES	NO	NO	NO	YES
4) Oral Ulcers	NO	NO	YES	NO	NO
5) Arthritis	YES	YES	YES	YES	YES
6) Serositis	YES	NO	YES	NO	YES
7) Renal disorder	YES	YES	YES	NO	NO
Class*	IV	III	V	NA	NA
8) Neurologic disorder	NO	NO	NO	NO	NO
9) Hematologic disorder	NO	NO	YES	YES	YES
10) Immunologic disorder	YES	NO	YES	YES	NO
11) Positive ANA	YES	YES	YES	YES	YES
Autoantibodies:					
Anti-DNA	YES	NO	YES	YES	NO
Anti-Sm	NO	NO	YES	NO	NO
Anti-RNP	NO	NO	YES	NA	NA
Anti-SSA	NO	NO	YES	NO	YES
Anti-SSB	NO	NO	YES	NO	NO
Anti-cardiolipin IgG	NO	NO	NO	NO	YES
Anti-beta2-glycoprotein I IgG	YES	NO	NO	NO	YES
Rheumatoid factor (RF)	NO	NO	NO	NA	NA
Lupus anticoagulant (LAC)	NO	NO	NO	NA	NA
SLICC Damage Index (Gladman et al., 1996)	1	5	1	0	3
Lung involvement:					
Interstitial lung disease (ILD) clinical diagnosis	NO	NO	NO	NO	NO
CT-scan performed	NO	NO	YES	NO	NO
Chest x-ray performed	YES, several (lung x-ray)	YES	YES	YES	YES
Other lung disease/symptoms	Relapsing pleuritis	Asthma	Pleuritis previously; breathlessness currently; emphysema (lower lobes); air trappning (upper lobes)	NO	NO

ACR: the American College of Rheumatology
SLICC: The Systemic Lupus Erythematosus International Collaborating Clinics
NA: not available
*Biopsies were classified according to the WHO or the ISN/RPS 2003 classification systems
