# Supplementary material for: Molecular pathways in patients with systemic lupus erythematosus revealed by gene-centred DNA sequencing
Source: Ann Rheum Dis. 2020 Oct 9;80(1):109–17. doi: 10.1136/annrheumdis-2020-218636 (PMC7788061; doi:10.1136/annrheumdis-2020-218636)
Supplement: Supplementary data [file annrheumdis-2020-218636supp003.pdf]

Molecular Pathways in Patients with Systemic Lupus Erythematosus Revealed by Gene Centred DNA Sequencing

Sandling et al.

Contents:

Supplementary Figures S1-S8

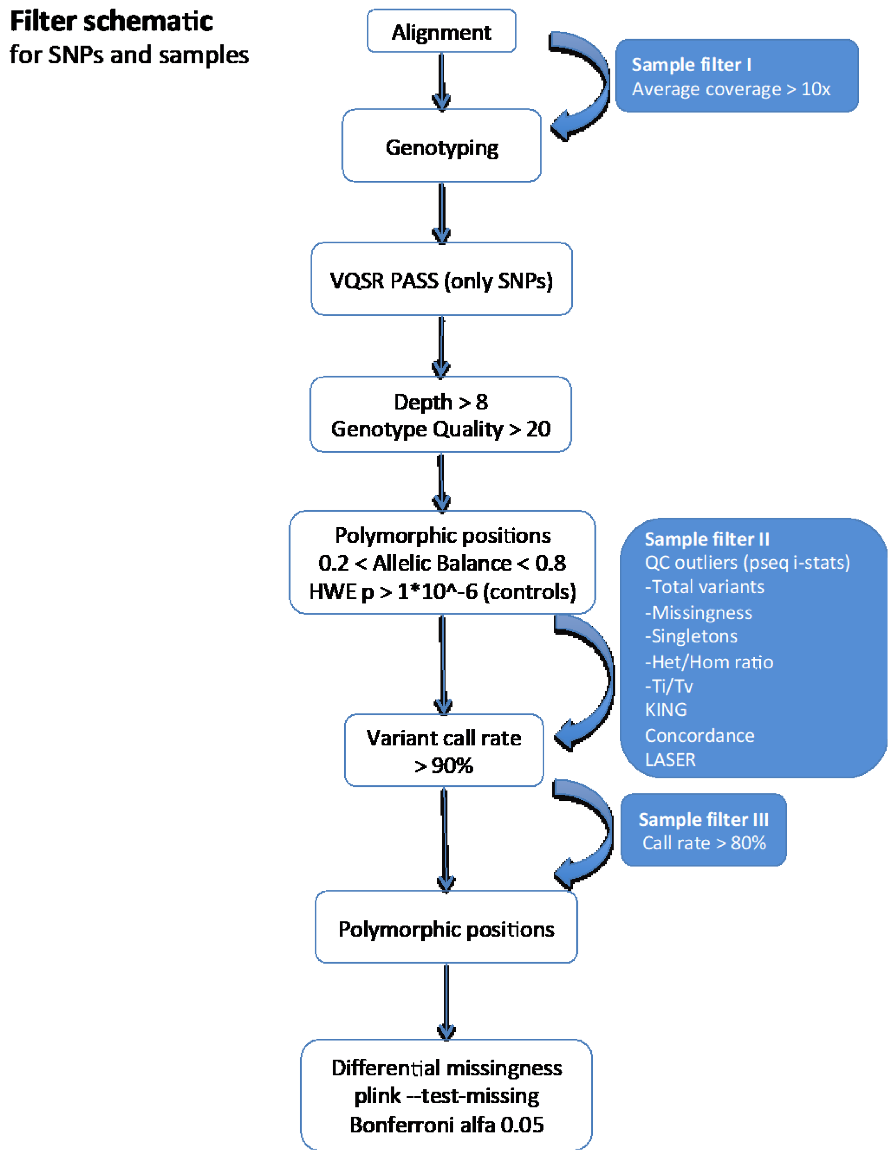

Supplementary Figure S1. Targeted sequencing variant calling and quality control pipeline.

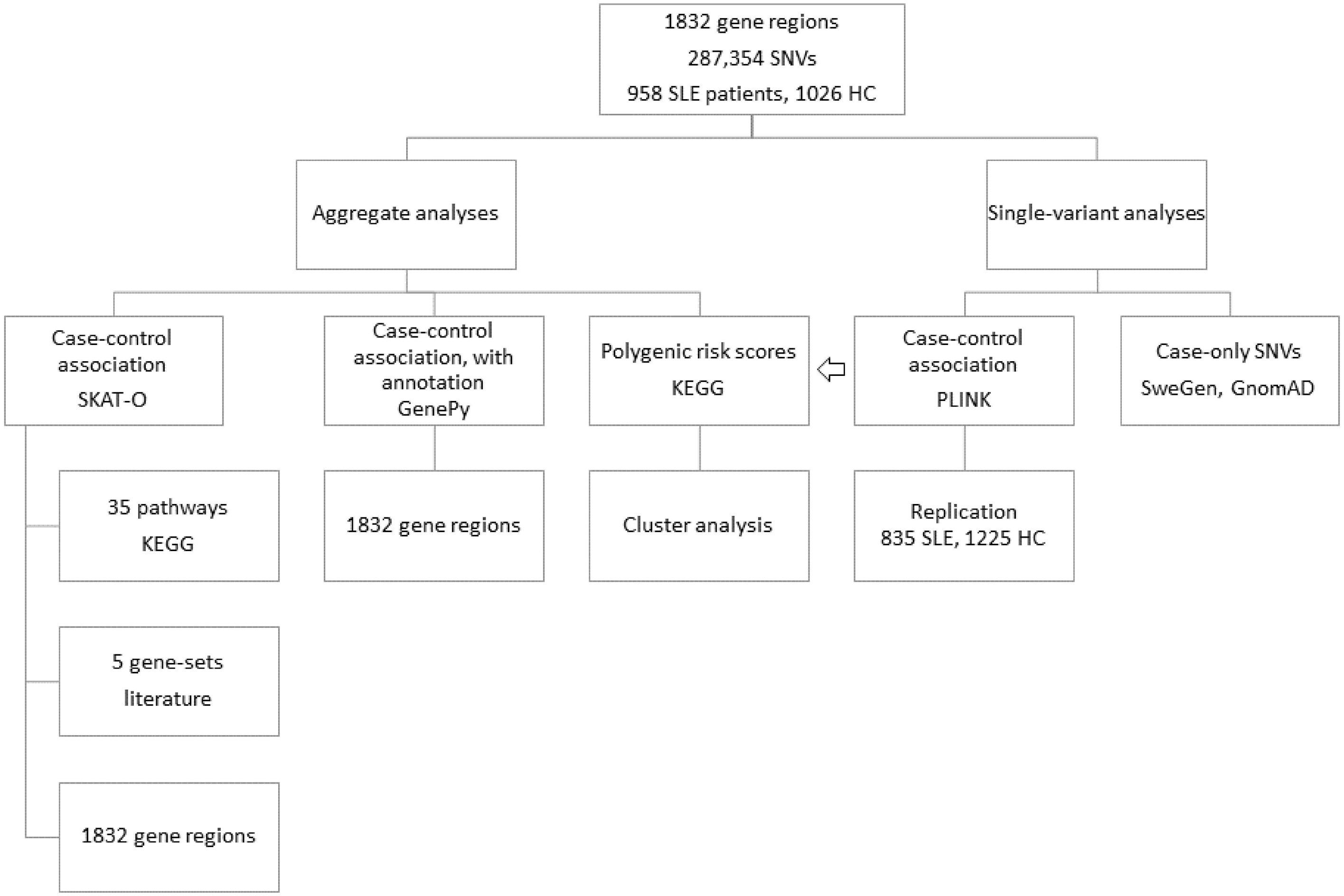

Supplementary Figure S2.Overview of analyses.

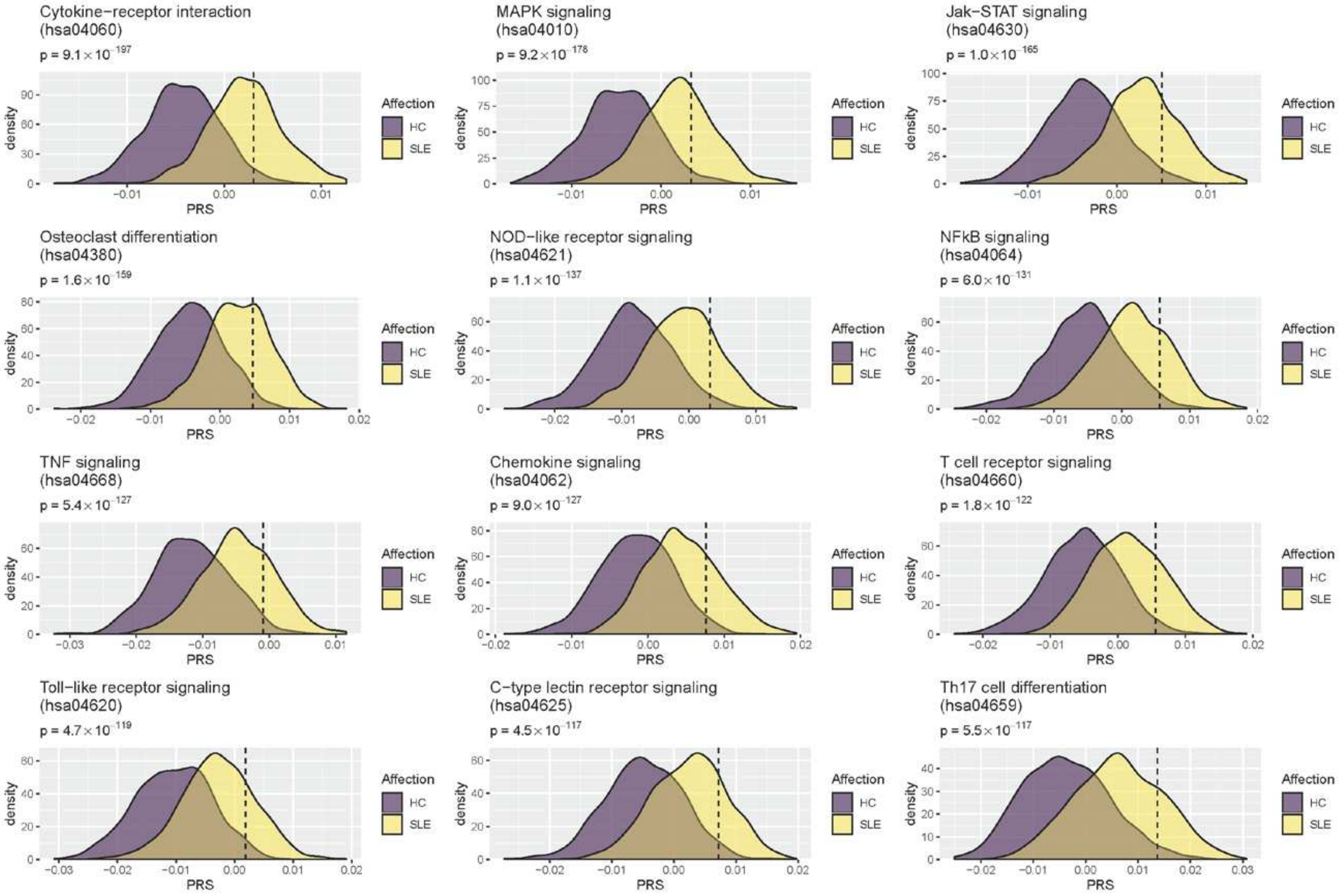
Cytokine−receptor interaction
(hsa04060)
p = 9.1 × 10^-197
MAPK signaling
(hsa04010)
p = 9.2 × 10^-178
Jak−STAT signaling
(hsa04630)
p = 1.0 × 10^-165
Osteoclast differentiation
(hsa04380)
p = 1.6 × 10^-159
NOD−like receptor signaling
(hsa04621)
p = 1.1 × 10^-137
NFkB signaling
(hsa04064)
p = 6.0 × 10^-131
TNF signaling
(hsa04668)
p = 5.4 × 10^-127
Chemokine signaling
(hsa04062)
p = 9.0 × 10^-127
T cell receptor signaling
(hsa04660)
p = 1.8 × 10^-122
Toll−like receptor signaling
(hsa04620)
p = 4.7 × 10^-119
C−type lectin receptor signaling
(hsa04625)
p = 4.5 × 10^-117
Th17 cell differentiation
(hsa04659)
p = 5.5 × 10^-117
density
PRS
Affection
HC
SLE

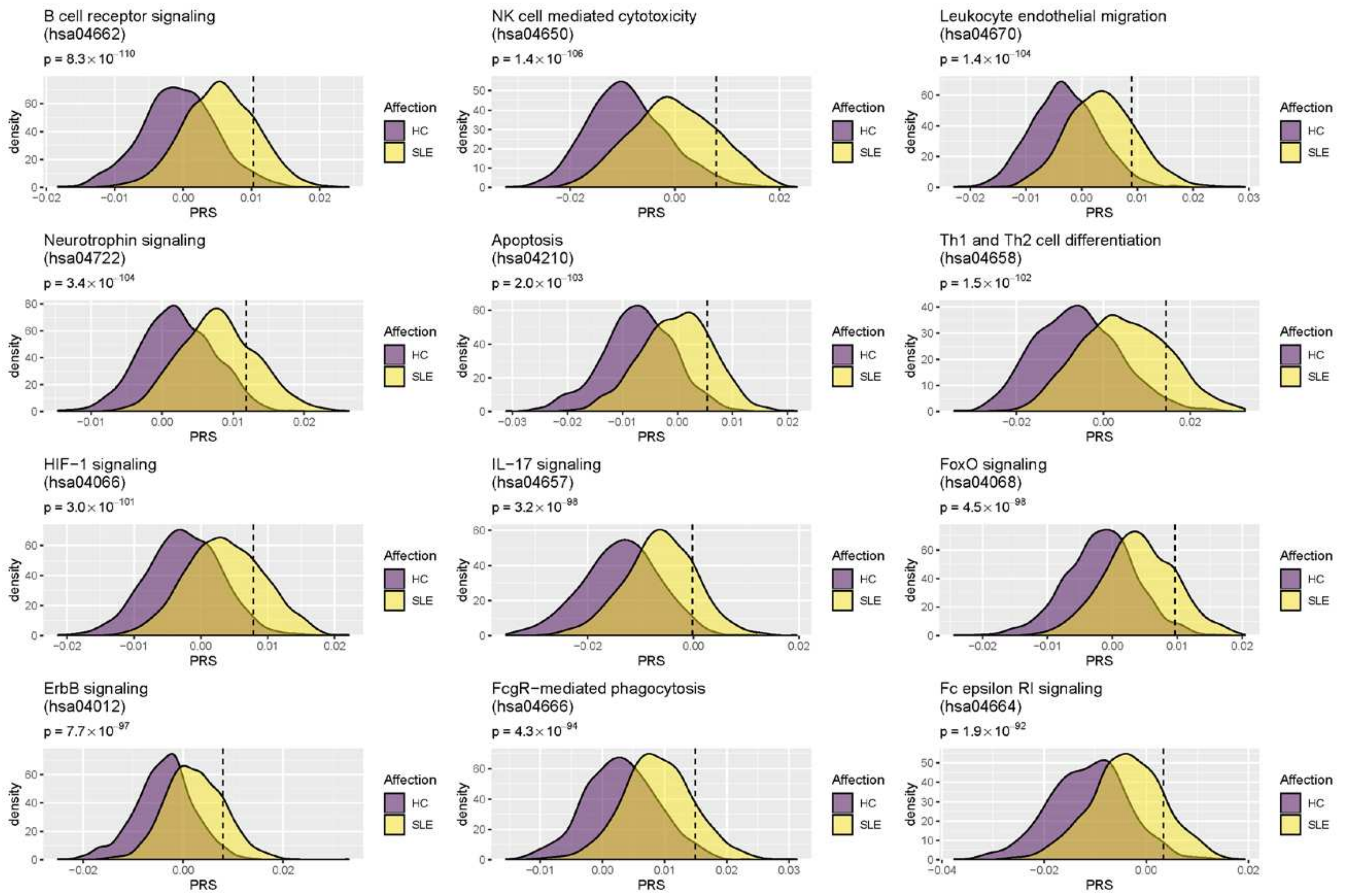
B cell receptor signaling
(hsa04662)
p = 8.3 × 10^-110
NK cell mediated cytotoxicity
(hsa04650)
p = 1.4 × 10^-106
Leukocyte endothelial migration
(hsa04670)
p = 1.4 × 10^-104
Neurotrophin signaling
(hsa04722)
p = 3.4 × 10^-104
Apoptosis
(hsa04210)
p = 2.0 × 10^-103
Th1 and Th2 cell differentiation
(hsa04658)
p = 1.5 × 10^-102
HIF−1 signaling
(hsa04066)
p = 3.0 × 10^-101
IL−17 signaling
(hsa04657)
p = 3.2 × 10^-98
FoxO signaling
(hsa04068)
p = 4.5 × 10^-98
ErbB signaling
(hsa04012)
p = 7.7 × 10^-97
FcgR−mediated phagocytosis
(hsa04666)
p = 4.3 × 10^-94
Fc epsilon RI signaling
(hsa04664)
p = 1.9 × 10^-92
density
PRS
Affection
HC
SLE

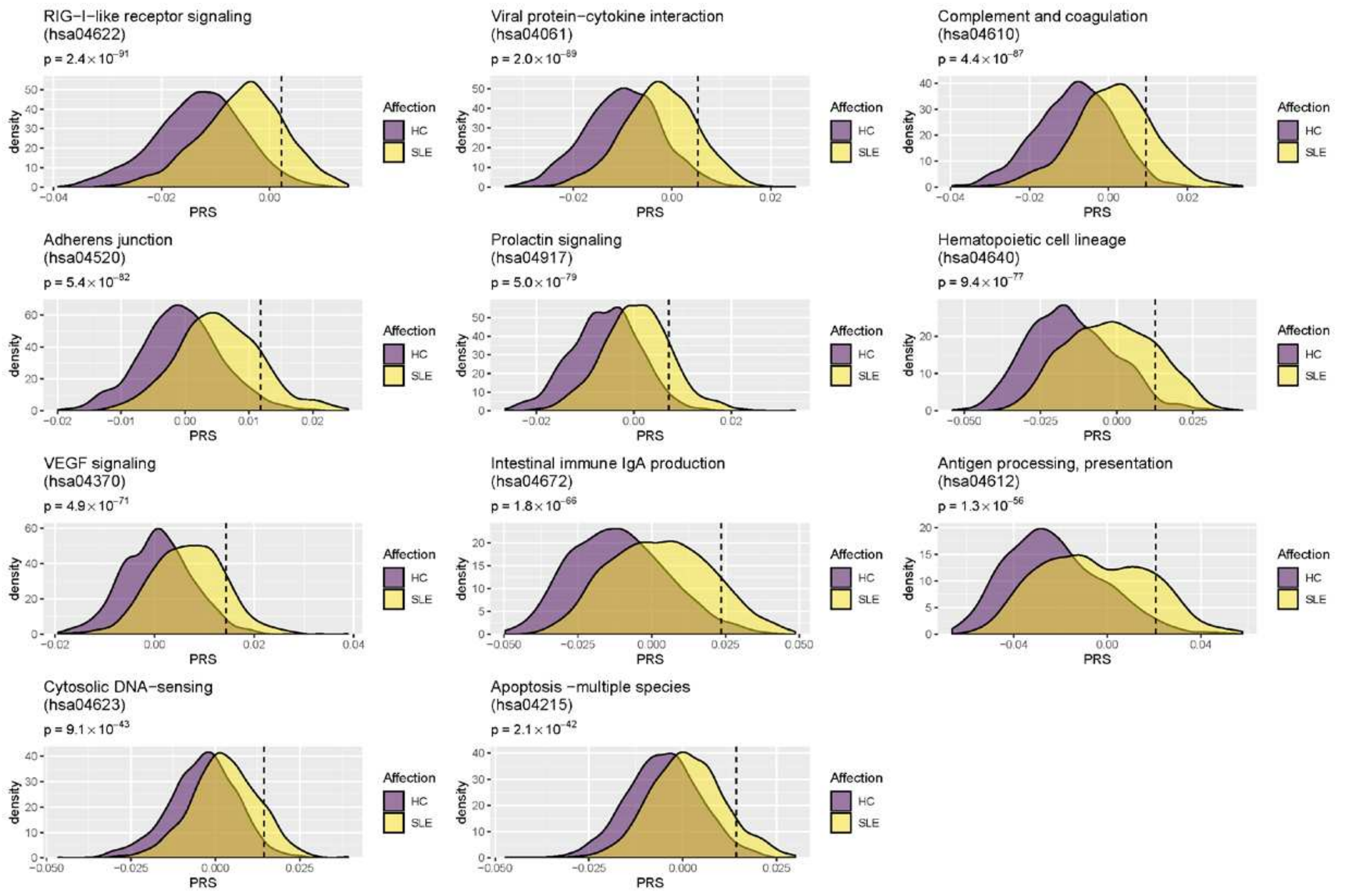

Supplementary Figure S3. Pathway polygenic risk score density plots for SLE patients and controls for each tested KEGG pathway. P-values represent differences in PRS between SLE patients (SLE) and control individuals (HC), uncorrected P-values are presented (Bonferroni corrected threshold P=0.00143). The dashed line indicates the PRS 97.5 percentile in control individuals.

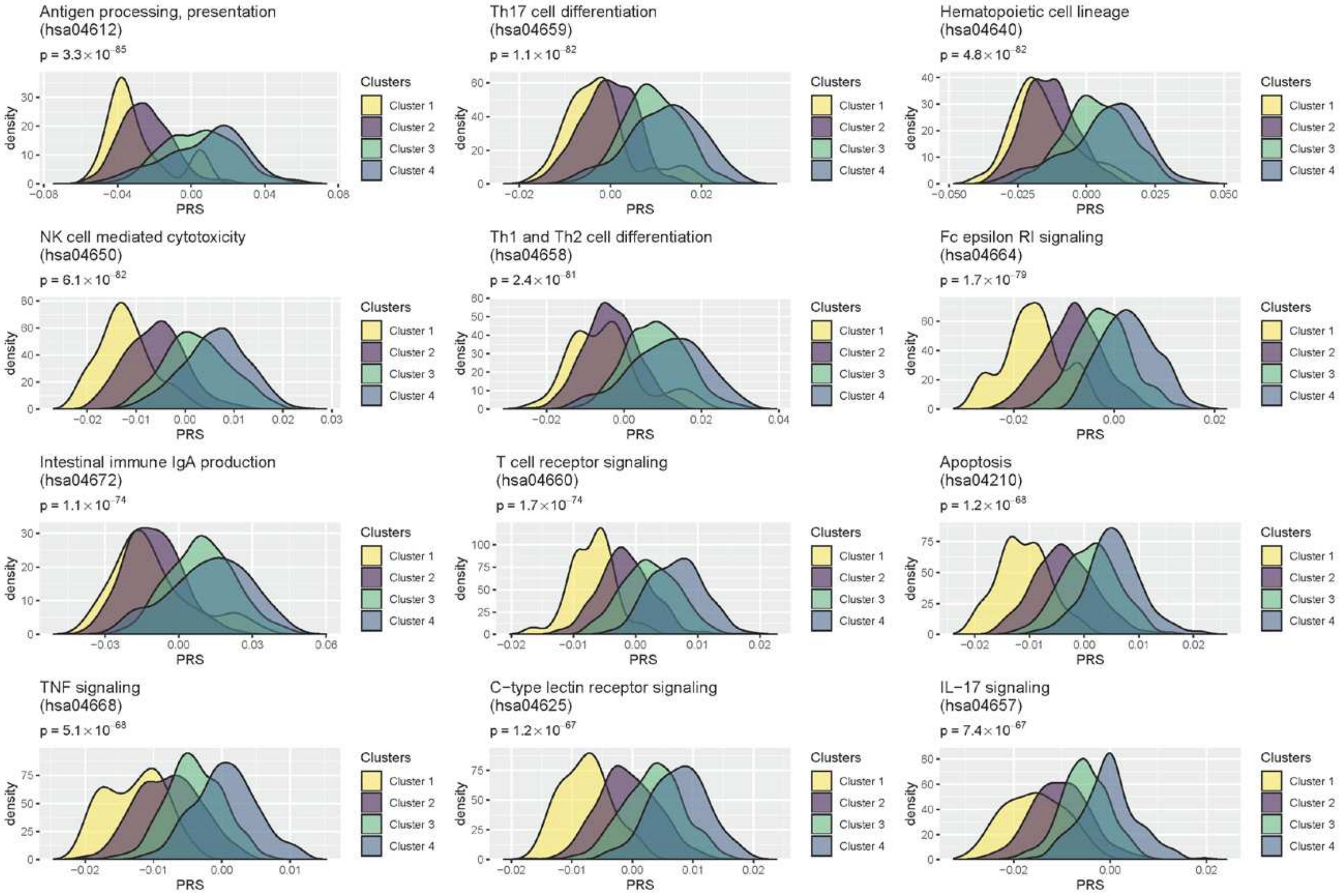
Antigen processing, presentation (hsa04612)
$p = 3.3 \times 10^{-85}$
Th17 cell differentiation (hsa04659)
$p = 1.1 \times 10^{-82}$
Hematopoietic cell lineage (hsa04640)
$p = 4.8 \times 10^{-82}$
NK cell mediated cytotoxicity (hsa04650)
$p = 6.1 \times 10^{-82}$
Th1 and Th2 cell differentiation (hsa04658)
$p = 2.4 \times 10^{-81}$
Fc epsilon RI signaling (hsa04664)
$p = 1.7 \times 10^{-79}$
Intestinal immune IgA production (hsa04672)
$p = 1.1 \times 10^{-74}$
T cell receptor signaling (hsa04660)
$p = 1.7 \times 10^{-74}$
Apoptosis (hsa04210)
$p = 1.2 \times 10^{-68}$
TNF signaling (hsa04668)
$p = 5.1 \times 10^{-68}$
C−type lectin receptor signaling (hsa04625)
$p = 1.2 \times 10^{-67}$
IL−17 signaling (hsa04657)
$p = 7.4 \times 10^{-67}$
density
PRS
Clusters
Cluster 1
Cluster 2
Cluster 3
Cluster 4

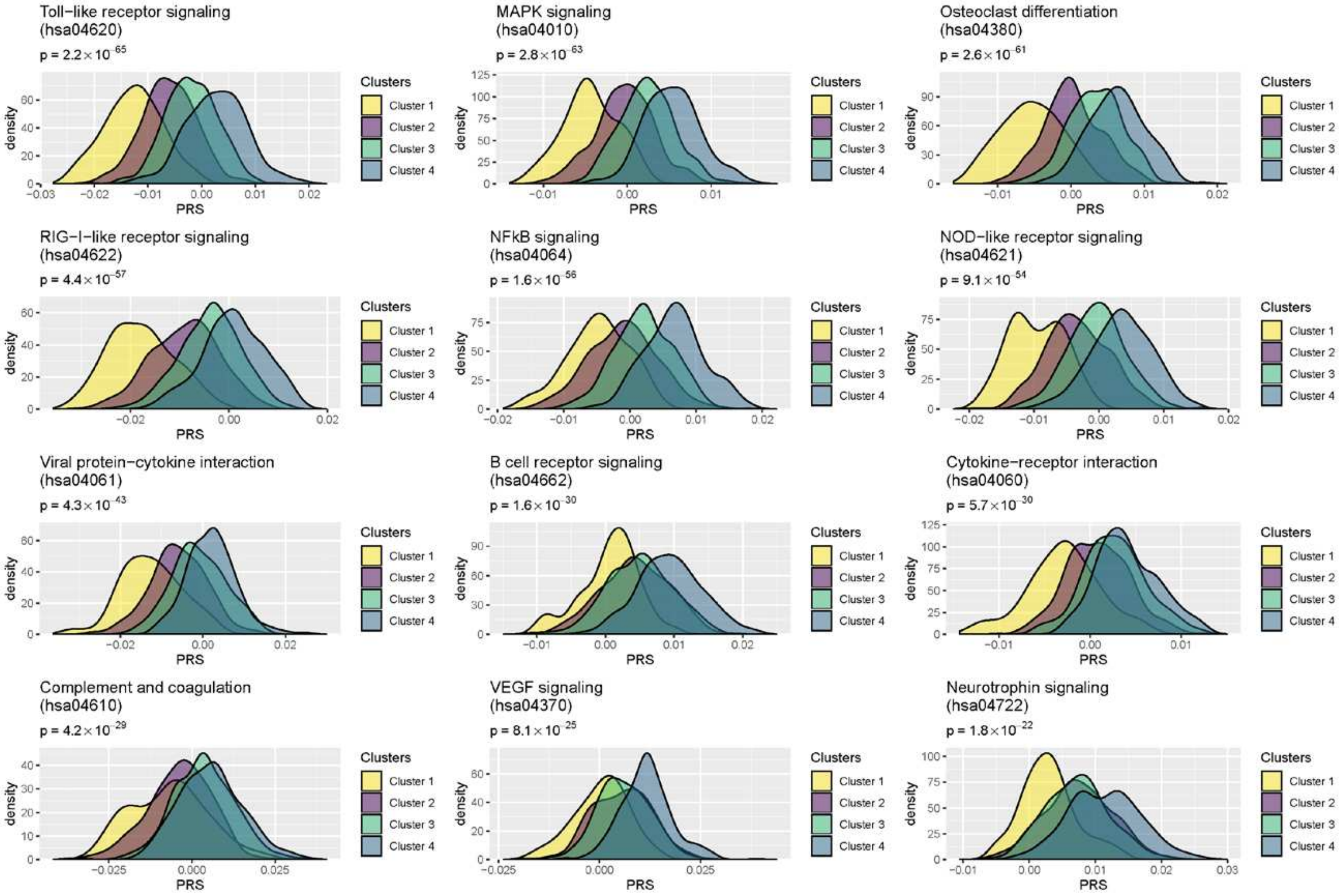
Toll−like receptor signaling
(hsa04620)
p = 2.2 × 10⁻⁶⁵
MAPK signaling
(hsa04010)
p = 2.8 × 10⁻⁶³
Osteoclast differentiation
(hsa04380)
p = 2.6 × 10⁻⁶¹
RIG−I−like receptor signaling
(hsa04622)
p = 4.4 × 10⁻⁵⁷
NFkB signaling
(hsa04064)
p = 1.6 × 10⁻⁵⁶
NOD−like receptor signaling
(hsa04621)
p = 9.1 × 10⁻⁵⁴
Viral protein−cytokine interaction
(hsa04061)
p = 4.3 × 10⁻⁴³
B cell receptor signaling
(hsa04662)
p = 1.6 × 10⁻³⁰
Cytokine−receptor interaction
(hsa04060)
p = 5.7 × 10⁻³⁰
Complement and coagulation
(hsa04610)
p = 4.2 × 10⁻²⁹
VEGF signaling
(hsa04370)
p = 8.1 × 10⁻²⁵
Neurotrophin signaling
(hsa04722)
p = 1.8 × 10⁻²²
Clusters
Cluster 1
Cluster 2
Cluster 3
Cluster 4
density
PRS

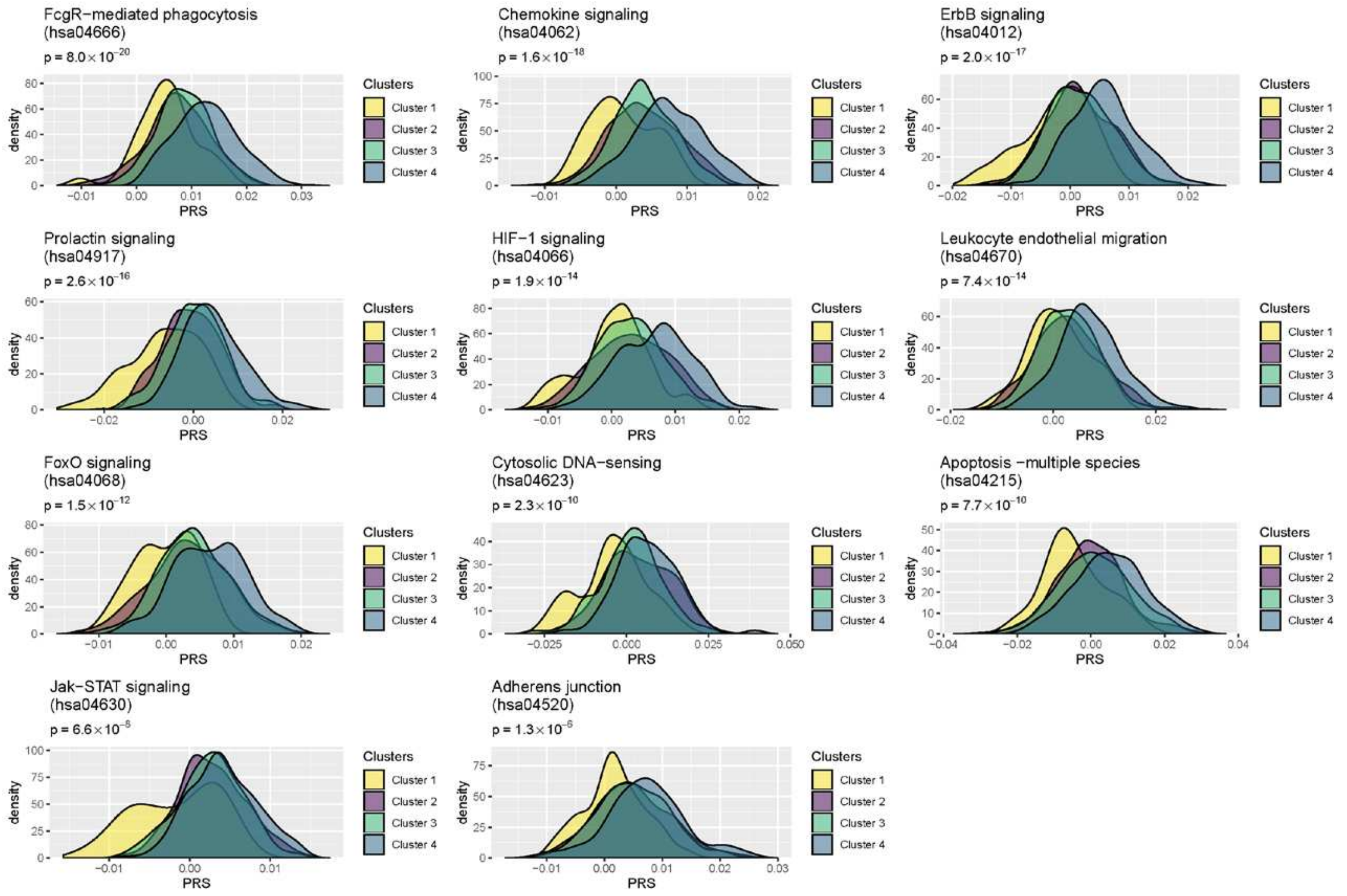

Supplementary Figure S4. Pathway SLE polygenic risk score density plots for the four groups of SLE patients identified for each tested KEGG pathway. P-values represent differences in PRS between clusters of SLE patients, uncorrected P-values are presented (Bonferroni corrected threshold P=0.00143).

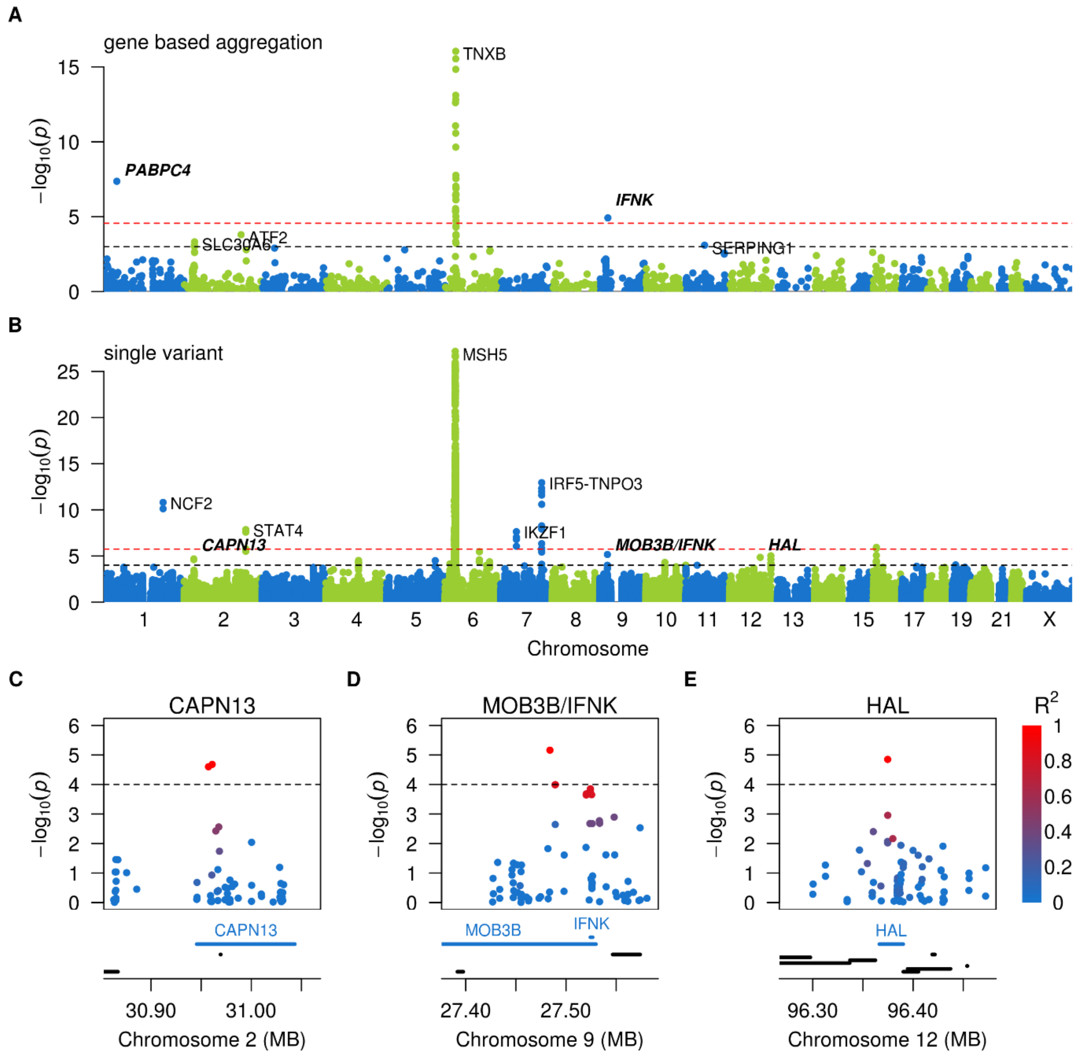

Supplementary Figure S5. Results of SLE case-control association analyses with P-values for association plotted against chromosomal location: a) Gene-based aggregate association testing (SKAT-O) where each point represents a gene region. Gene names are indicated for the top gene regions. The red line represents a Bonferroni corrected significance threshold and the black line FDR 0.05. Novel loci are indicated in bold. b) Single variant association testing where each point represents a SNV. The red line represents a Bonferroni corrected significance threshold and the black line the suggestive significance threshold ($P<1\times10^{-4}$). Novel loci are indicated in bold. c-e) Single variant association result regional association plots for the *CAPN13*, *MOB3B/IFNK* and *HAL* regions respectively. The colour scale indicates linkage disequilibrium (r^2) between SNVs.

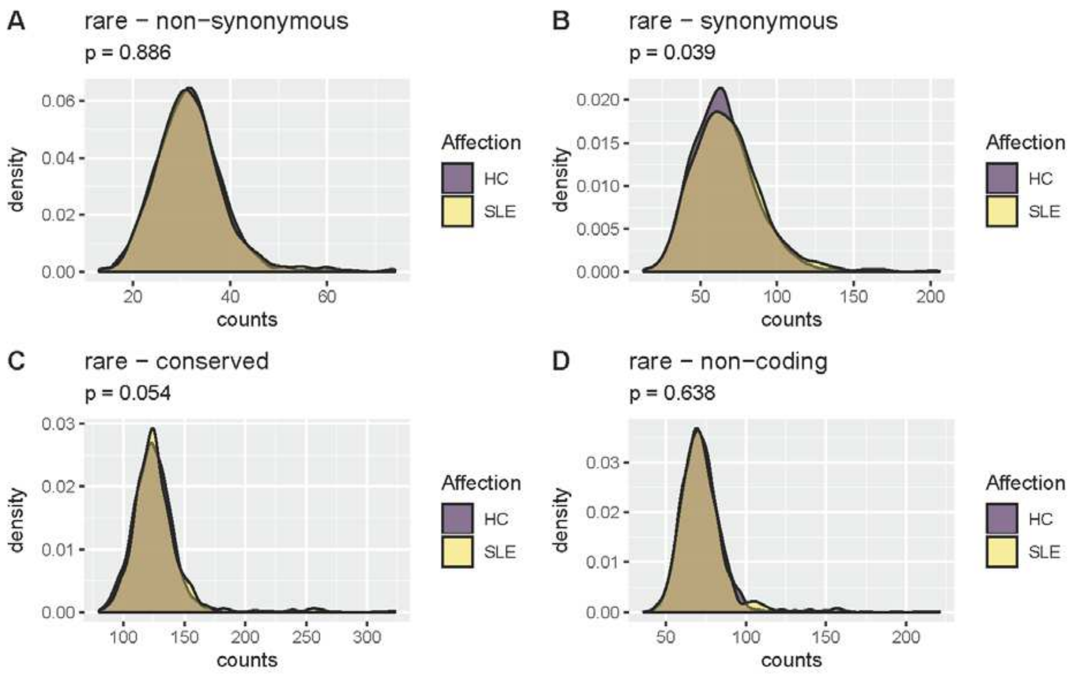

Supplementary Figure S6. Distribution of different classes of SNVs in SLE patients and control individuals: a) rare non-synonymous variants, b) rare synonymous variants, c) rare constrained variants (GERP RS score >2), d) rare non-coding variants (any of the following snpeff annotations: sequence feature, upstream, downstream, intergenic, TF binding site variant). P-values represent difference between SLE patients and control individuals, uncorrected P-values are presented (Bonferroni corrected threshold P=0.0125).

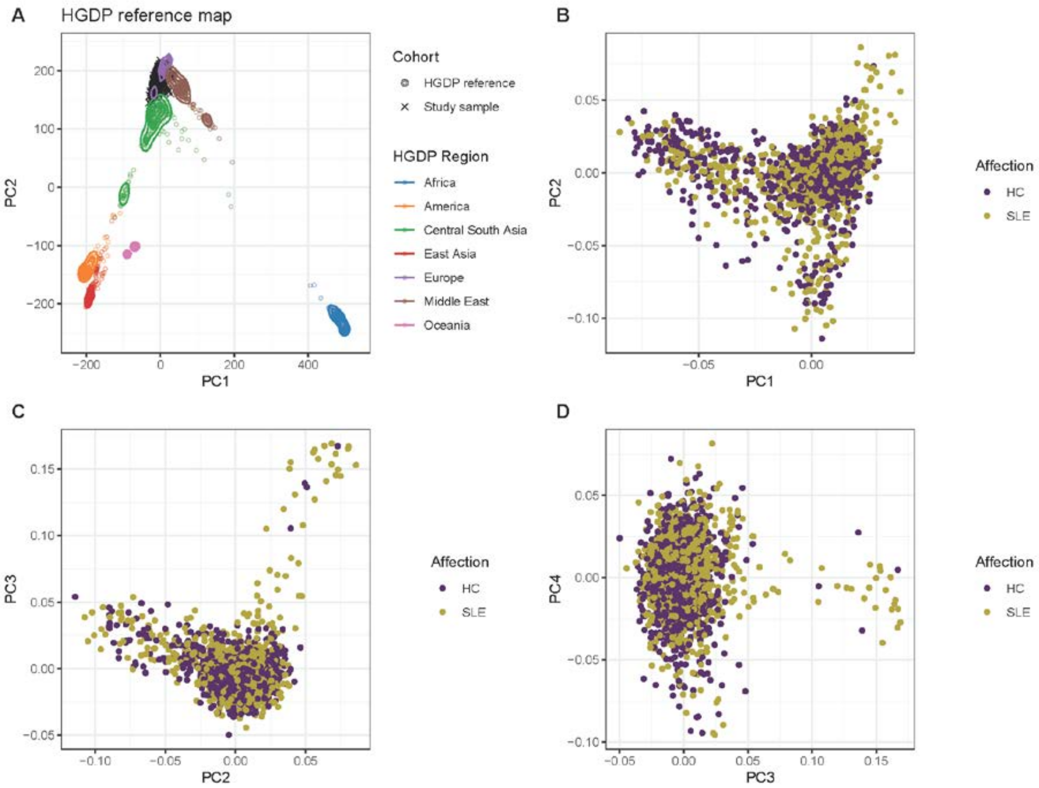

Supplementary Figure S7. Genetic population structure of study individuals. a) Study samples mapped on population reference samples. b) Principal components for population stratification within study, PC1 vs PC2 c) PC2 vs PC3 d) PC3 vs PC4.

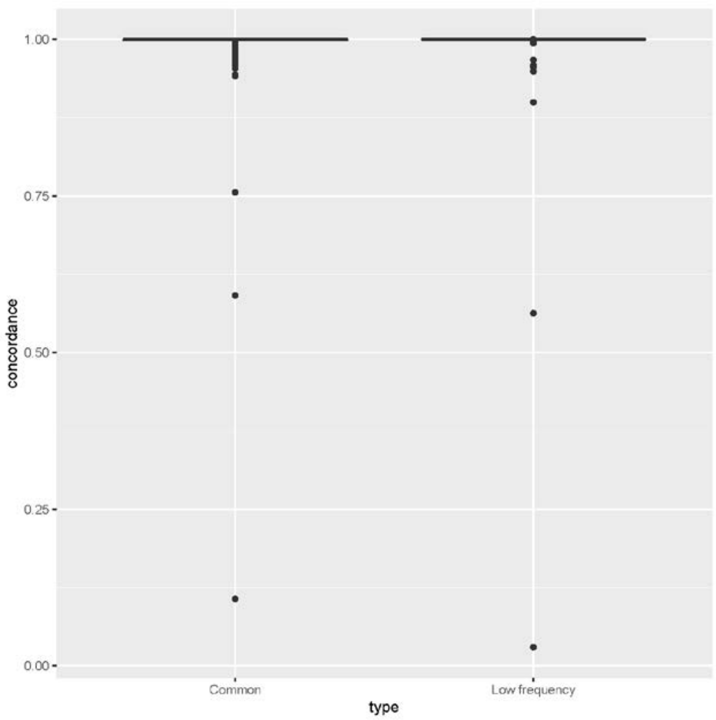

Supplementary Figure S8. SNV genotype average concordance between targeted sequencing and genotyping by a beadchip array (Illumina ImmunoChip). Concordance for two types of SNVs, common SNVs (MAF≥0.05) and low frequency SNVs (MAF<0.05), are displayed.
